# Profiling the Dynamics of a Human Phosphorylome Reveals New Components in HGF/c-Met Signaling

**DOI:** 10.1371/journal.pone.0072671

**Published:** 2013-09-02

**Authors:** Crystal L. Woodard, C. Rory Goodwin, Jun Wan, Shuli Xia, Robert Newman, Jianfei Hu, Jin Zhang, S. Diane Hayward, Jiang Qian, John Laterra, Heng Zhu

**Affiliations:** 1 Department of Pharmacology and Molecular Sciences, Johns Hopkins School of Medicine, Baltimore, Maryland, United States of America; 2 Department of Neuroscience, Johns Hopkins School of Medicine, Baltimore, Maryland, United States of America; 3 Department of Neurology, Kennedy Krieger Institute, Baltimore, Maryland, United States of America; 4 Department of Ophthalmology, Johns Hopkins School of Medicine, Baltimore, Maryland, United States of America; 5 Department of Oncology, Johns Hopkins School of Medicine, Baltimore, Maryland, United States of America; 6 Department of Neurology, Johns Hopkins School of Medicine, Baltimore, Maryland, United States of America; 7 High Throughput Biology Center, Johns Hopkins School of Medicine, Baltimore, Maryland, United States of America; University of Colorado, United States of America

## Abstract

Protein phosphorylation is a dynamic and reversible event that greatly influences cellular function. Identifying the key regulatory elements that determine cellular phenotypes during development and oncogenesis requires the ability to dynamically monitor proteome-wide events. Here, we report the development of a new strategy to monitor dynamic changes of protein phosphorylation in cells and tissues using functional protein microarrays as the readout. To demonstrate this technology's ability to identify condition-dependent phosphorylation events, human protein microarrays were incubated with lysates from cells or tissues under activation or inhibition of c-Met, a receptor tyrosine kinase involved in tissue morphogenesis and malignancy. By comparing the differences between the protein phosphorylation profiles obtained using the protein microarrays, we were able to recover many of the proteins that are known to be specifically activated (i.e., phosphorylated) upon c-Met activation by the hepatocyte growth factor (HGF). Most importantly, we discovered many proteins that were differentially phosphorylated by lysates from cells or tissues when the c-Met pathway was active. Using phosphorylation-specific antibodies, we were able to validate several candidate proteins as new downstream components of the c-Met signaling pathway in cells. We envision that this new approach, like its DNA microarray counterpart, can be further extended toward profiling dynamics of global protein phosphorylation under many different physiological conditions both *in cellulo* and *in vivo* in a high-throughput and cost-effective fashion.

## Introduction

Protein post-translational modification (PTM) is one of the most important molecular mechanisms that regulate almost every aspect of cellular processes in eukaryotes. Common PTMs, such as phosphorylation, ubiquitylation, SUMOylation, acetylation, and glycosylation, alter protein conformation, surface charge, stability, and molecular interactions and thereby, profoundly impact protein function [1]. As one of the most important PTMs, reversible phosphorylation is highly dynamic and plays a dominant role in signaling pathways and many other molecular processes [2]. Up to 40% of the human proteome has been estimated as phosphoproteins modified by approximately 525 annotated protein kinases. Protein phosphorylation is usually tightly controlled and regulated; dysregulation of kinase signaling pathways often results in cancer and other malignancy [3]. Kinase signaling pathways, such as PI3K/Akt, Raf/MEK/ERK, and JAK/Stat, are known to be implicated in tumor formation and progression [4] [5] [6]. However, it is important to note that signaling pathways do not operate in isolation, but in a cooperative manner. Therefore, the ability to capture the collective activity of kinases, through the identification of phosphorylated proteins, under multiple conditions in a high-throughput and cost-effective fashion would greatly facilitate our understanding of the complex signaling networks in cells.

Recently, extensive proteomic approaches, such as 2-D gel- and LC-coupled tandem mass spectrometry analyses (MS/MS), have been developed to profile global phosphorylation events (i.e., phosphorylome) associated with a particular disease [7]. Multiple fractionation techniques are often required due to the multifaceted composition of the samples. Although these MS/MS-based techniques are capable of capturing dynamic changes across different conditions, it is still cumbersome to survey a large number of samples under multiple conditions because of the high-cost and time-consuming nature of the technology. An alternative approach is to use the DNA microarray equivalent, namely antibody microarrays, which utilize immobilized antibodies on a glass surface to determine the concentration of specific phosphopeptides in question [8]. Though powerful, this approach is limited to the availability and quality (e.g., specificity and affinity) of the antibodies.

We and others have demonstrated that functional protein microarrays, composed of individually purified proteins on a glass surface, are a powerful tool for analyzing the biochemical properties of proteins [9]–[16]. In particular, these arrays have served as an ideal platform to identify substrates of purified protein posttranslation modification (PTM) enzymes, such as protein acetyltransferases, kinases, and ubiquitin E3 ligases [19]–[21]. However, assays that include enzymatic co-factors and preserve macromolecular interactions are required to obtain an integrated view of the overall enzymatic activity (i.e., phosphorylome) in a cell [22]–[24]. Here, we describe a new approach to identify condition-dependent phosphorylation events by comparing microarray based phosphorylation profiles obtained with kinase assays using lysates from cells or tissues. This new method allowed us to monitor the dynamic changes in cell and tissue phosphorylomes associated with activation of the hepatocyte growth factor (HGF)/c-Met signaling relevant to malignancy, particularly glioblastoma multiforme. Subsequent *in vivo* validations confirmed the identification of novel downstream phosphoproteins regulated by c-Met and provided new insights into the molecular mechanism of cancer cell proliferation.

## Materials and Methods

### Protein purification and human protein microarrays

Human proteins were purified as 6×-GST-His-fusion proteins from yeast using a high-throughput protein purification protocol described previously [17].

### Tissue culture preparation

U373MG HGF^−^/C-Met^+^ cells were maintained in Dulbecco's Modified Essential Medium low glucose with L-glutamine and sodium pyruvate (DMEM; Mediatech Inc. Inc.) supplemented with 10% fetal bovine serum and 1% penicillin-streptomycin as previously described [25]. All cells were grown at 37°C in a humidified incubator with 5% CO_2_. U373 cells were incubated in the aforementioned media supplemented with low serum (0.1% fetal bovine serum) overnight and treated with 50 ng/µL HGF or PBS control for 30 min. At the end of each experiment, cells were washed and harvested in 1X PBS in preparation for protein extraction for phosphorylation assays and immunoblot analyses.

### Tumor xenografts

Glioma xenografts were generated as previously described [25] from U87MG HGF^+^/C-Met^+^ cell lines originally obtained from the American Type Culture Collection. Cells were grown in MEM with Earle Salts and L-glutamine (MEM 1×; Mediatech, Inc.) supplemented with 10% fetal bovine serum (Gemini Bioproducts, Inc.), 2 mmol/L sodium pyruvate (Mediatech, Inc.), 0.1 mmol/L MEM-nonessential amino acids (Mediatech, Inc.), and penicillin-streptomycin (Mediatech, Inc.) at 37°C in a humidified incubator with 5% CO_2_. Female 6- to 8-week-old mice (National Cancer Institute, Frederick, MD) were anesthetized by i.p. injection of ketamine (100 mg/kg) and xylazine (5 mg/kg). For subcutaneous xenografts, Nu/nu mice received 4×10^6^ cells in 0.05 mL of plain media s.c. in the dorsal flank. When tumors reached ∼200 mm^3^, the mice were randomly divided into groups (*n* = 4 per group) and received the indicated doses of either L2G7 or isotype matched control mAb (5G8) in 0.1 ml PBS i.p., as previously described [26]. Tumor volumes were estimated by measuring two dimensions [length (*a*) and width (*b*)] and calculating volume as *V* = *ab* ^2^/2 [26] [27]. At the end of each experiment, tumors were excised, frozen in liquid nitrogen and protein was extracted for phosphorylation assays and immunoblot analyses. Animals were housed in a pathogen-free facility. The Johns Hopkins University Institutional Animal Care and Use Committee approved all animal protocols used in this study.

### Whole cell lysate preparation

Total protein was extracted at 4°C from HEK293T cells, glioma cells, or glioma xenografts using radioimmunoprecipitation assay (RIPA) buffer (1% Igepal, 0.5% sodium deoxycholate, and 0.1% SDS in PBS) containing fresh 1× protease and 1× phosphatase inhibitor cocktails (Calbiochem). Cell culture and tissue extracts were sonicated on ice and centrifuged at 5,000 RPM at 4°C for 5 min. Supernatants were assayed for protein concentrations by Coomassie protein assay (Pierce) according the manufacturer's recommendations.

### Optimization of phosphorylation assay using whole cell lysates

The human protein microarrays were blocked in 1X TBST with 0.1% BSA (Roche) for 1 hr at RT with gentle shaking. Total protein lysates were then diluted into 250 µL kinase buffer (50 mM Tris-HCl and 25 mM Hepes-KOH at pH 7.5) containing 100 mM NaCl, 10 mM MgCl_2_, 1 mM MnCl_2_, 1 mM DTT, 1 mM EGTA, 2 mM Na_3_VO_4_, 2 mM NaF, 0.01 mM cold ATP, and γ-^32^P-ATP [33.3 nM final concentration]) to specified total concentrations (0.0004–0.8 µg/µL) for optimizing kinase activity on protein microarrays. Each reaction mixture was then incubated on microarrays and cover slipped before being placed in a humidity chamber at 30°C for specified times (10, 30, and 60 min). Each reaction was performed in duplicate. Following the termination of the reaction, the slides were subjected to three-10 min washes in 1X TBS/T [pH 7.5], three-10 min washes in 0.5% SDS, and one quick rinse with distilled water before being spun dry and exposed to X-ray film (Kodak). Control slides were incubated with kinase reaction mixture without kinase and processed in parallel. Exposures were taken for each microarray assayed at 10, 17, and 24 hrs.

### Phosphorylation using U87MG, and U373MG cell lysates

Protein microarrays were blocked in 1X TBS/T with 0.1% BSA (Roche) for 1 hr at RT with gentle shaking. 0.2 µg/µL of total lysate was diluted into kinase buffer. Lysate-buffer mixture was then incubated on arrays and cover slipped before being placed in a humidity chamber at 30°C for 30 min. Assays were performed in triplicate. The slides were subjected to three-10 min washes in 1X TBS/T [pH 7.5], three-10 min washes in 0.5% SDS, and one quick rinse with distilled water before being spun dry and exposed to X-ray film (Kodak). Control slides were incubated with kinase reaction mixture without kinase and processed in parallel. Arrays were developed by autoradiography for a short (10 hr) and long (24 hr) exposure time.

### Protein microarray data acquisition

After lysate assays were conducted on the protein microarrays and exposed to X-ray film, the film was scanned at 4,800 dots per inch. Digitized images in TIFF format were then analyzed using GenePix Pro 6.0 (Molecular Devices) and labeled Phospho-X. Post phosphorylation, each array was subsequently probed with anti-GST antibody, digitized with a GenePix 4000 scanner (MDS AnalyticalTechnologies, CA), analyzed using GenePix Pro 6.0, and labeled Anti-GST-X. Positive and negative control proteins (i.e., BSA and histone proteins) were imprinted on each array as markers to properly align the grids and check for non-specific phosphorylation events. To correct spots skewed by artifacts like dust or specks, the sizes and positions of all spots on the human transcription factor protein microarrays were adjusted and manually checked.

### Background correction and normalization of protein microarrays

The raw signal intensity of each spot was defined as the foreground median intensity divided by its local background median intensity as acquired using the GenePix software. There are over 1000 “empty” or “blank” spots on each microarray contributing to the background noise that may be picked up in the signal intensities. Our analyses showed that the distribution of raw signal intensities of all “empty” or “blank” spots on an array is approximately like a normal distribution with the mean value around 1. Assuming that the raw intensity distributions of these “empty” and “blank” spot (background noises) are the same across all microarrays (under different conditions), we may use a Z-score to set a universal cutoff for all conditions. Hence, the protein signals were standardized such that,
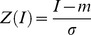
where *Z* is Z-score of each spot, *I* is raw intensity of the spot, *m* and σ are mean value and standard deviation, respectively, of “empty” and “blank” spots on the microarray.

### Elimination of printing bias

Multiple array comparisons were a critical component of the optimization of the dynamic kinase assays performed on the protein microarrays. To ensure the equality of the arrays used to perform the dynamic kinase assays, the signal intensities of each spot compared had to be the same and larger than a certain cutoff on all anti-GST arrays. Taking into consideration the signal intensity of each spot imprinted on the anti-GST X scan of each microarray, only proteins on the anti-GST microarrays that had a *Z* score larger than 3 were taken into account for further analyses. Positive and negative control proteins (i.e., BSA and histones) were excluded from the analyses.

### Identification of positive hits

For both *in vitro* and *in vivo* assays performed of the protein microarrays, a ΔZ score was calculated for each protein representing the Z-score difference between treatment and control experimental arrays. For selected ΔZ cutoff, *Z_0_*, we obtained a number of differentially phosphorylated HGF/c-Met proteins, *H*(*Z_0_*), and calculated corresponding p-values to see whether the number proteins recovered were random occurrences or with a bias. To determine an optimal cutoff, we calculated an enrichment score such that
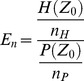
where *P*(*Z_0_*) is number of all differentially phosphorylated proteins under the cutoff *Z_0_*, and *n_H_* and *n_P_* are total numbers of HGF/c-Met proteins and all proteins on the chip, respectively. The final ΔZ cutoffs were then optimized by the maximum enrichment score recovered.

### Network construction of novel protein hits and c-Met signaling components

We constructed the network between novel protein hits and well-known c-Met signaling components by using the Cytoscape 2.8.2 software [28]. The protein-protein interactions (PPIs) used in the network were obtained from the website: http://string-db.org/. Parameters were set to include interactions derived only from experimentation. The known kinase-substrate interactions (KSIs) were compiled based on the data downloaded from two websites, Kinasource (http://kinasource.co.uk/Database/substrates.html) and Phospho.ELM (http://phospho.elm.eu.org/dataset.html) [29].

### Immunoblot analysis

Aliquots of 50 µg/µL total proteins were combined with Laemmli loading buffer containing ß-mercaptoethanol and subjected to SDS-polyacrylamide gel electrophoresis (PAGE) according to the method of Towbin et al. with some modifications [30] [31]. For immunoblot analyses, proteins were electrophoretically transferred to nitrocellulose using a semidry transfer apparatus, the iBlot® Dry Blotting System (Invitrogen) at 20 V for 7 min. Membranes were incubated for 1 hr in 5% milk at room temperature and then overnight with primary antibodies p44/42 MAPK (Erk1/2) (Cell Signaling Technology), phospho-p44/42 MAPK (Erk1/2) at Thr202 and Tyr204 (Cell Signaling Technology), AKT (Cell Signaling Technology), phospho-AKT at Ser473 (Cell Signaling Technology), phospho-eEF2K at Ser366 (Cell Signaling Technology), phospho- MAPK7 at Thr218 and Tyr220 (Cell Signaling Technology), phospho-PKCD at Thr505 (Cell Signaling Technology), phospho-p90RSK at Ser380 (Cell Signaling Technology), and phospho-PKA substrate (Cell Signaling Technology) at 4°C in 5% BSA in Tris-buffered saline (TBS) containing 0.1% Tween 20 (TBS/T). Membranes were then washed three times with TBS/T, incubated with secondary antibody tubulin (Sigma) or rabbit-anti-HRP at 1:5,000 for 1 hr in TBS/T, and washed three times with TBS/T. Proteins were detected and quantified using the Odyssey Infrared Imager (LI-COR Biosciences) or autoradiography.

## Results

### Optimization of phosphorylation using whole cell lysates on a protein microarray

**Figure 1:**
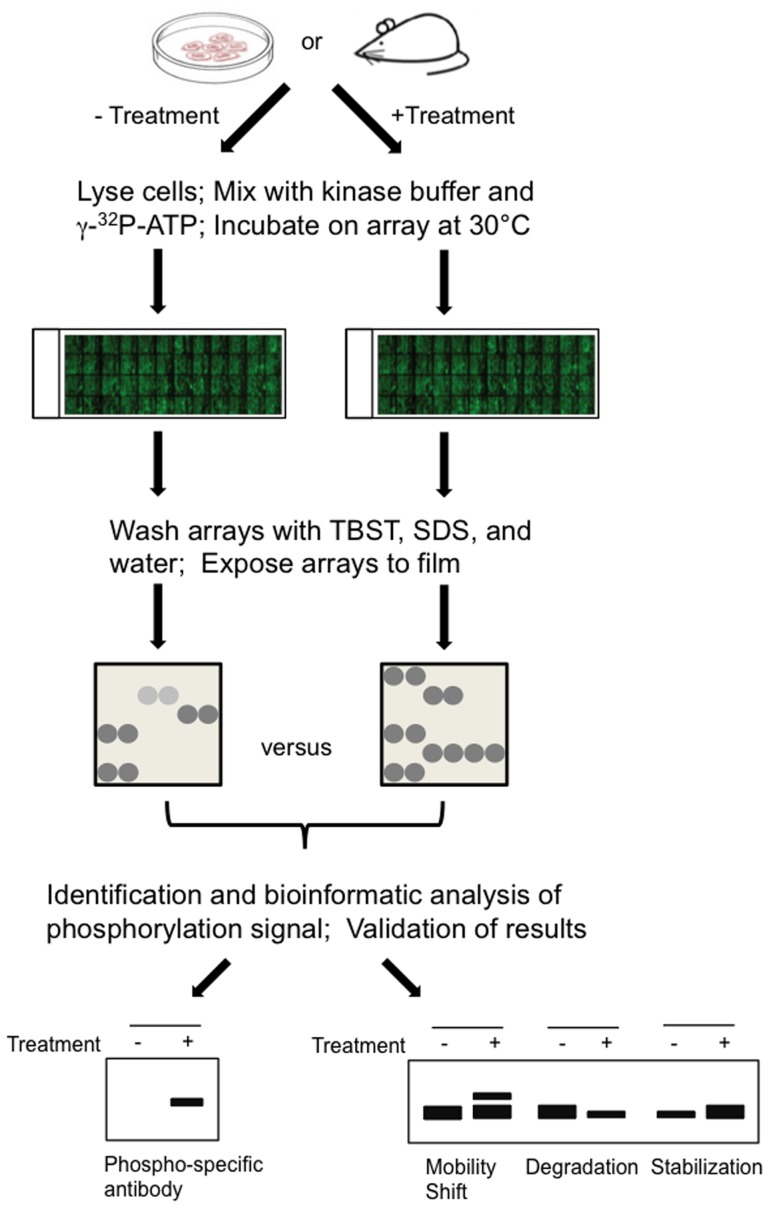
Scheme for profiling human phosphoproteome patterns using protein microarrays as readout. Cells are collected from *in cellulo* or *xenograft* model systems that were untreated or treated with a c-Met agonist or antagonist. Total proteins in lysed cells were then diluted to a kinase reaction buffer and applied to the human protein microarray. After the reaction is completed, the protein microarray is washed under stringent conditions, exposed to X-ray film, and the phosphorylation signals were acquired and analyzed GenePix software. Comparison of phosphorylation signals between different assays results in identification of differentially phosphorylated proteins associated with HGF/c-Met activation. Candidate proteins were then validated via traditional methods.

We used the whole cell lysates as an enzymatic source, in conjunction with previously reported human protein microarrays composed of 2,236 unique human proteins in full-length as potential substrates [17], to profile changes in the phosphorylation activity in cells. This approach would enable us to profile global phosphorylation events and to identify differentially phosphorylated proteins associated with a particular condition (Fig. 1). We first optimized the reaction conditions using glioblastoma (U87M) cells to ensure the platform's applicability to multiple cell types. The U87 cell line is HGF^+^/c-Met^+^, which makes the pathway constitutively active. Therefore, the lysate contains active kinases whose signaling will facilitate phosphorylation of the proteins on our array.

Concentrations of total cell proteins and phosphorylation reaction times were varied to determine the most favorable combination for detecting the largest number of phosphorylated proteins on the protein microarrays without reaching saturation conditions [2] [3]. The whole cell lysates were titrated and diluted to achieve final protein concentrations ranging from 0–0.8 µg/µL and then incubated on protein microarrays in the presence of ^32^P-γ-ATP for three time points (i.e., 10, 30, and 60 min). Reactions were terminated by extensively washing the microarrays with TBS/T and SDS buffers to ensure that the signals detected were a result of covalent phosphorylation. Phosphorylation signals were then acquired by autoradiography and analyzed by the GenePix software (Fig. S1).

**Figure 2:**
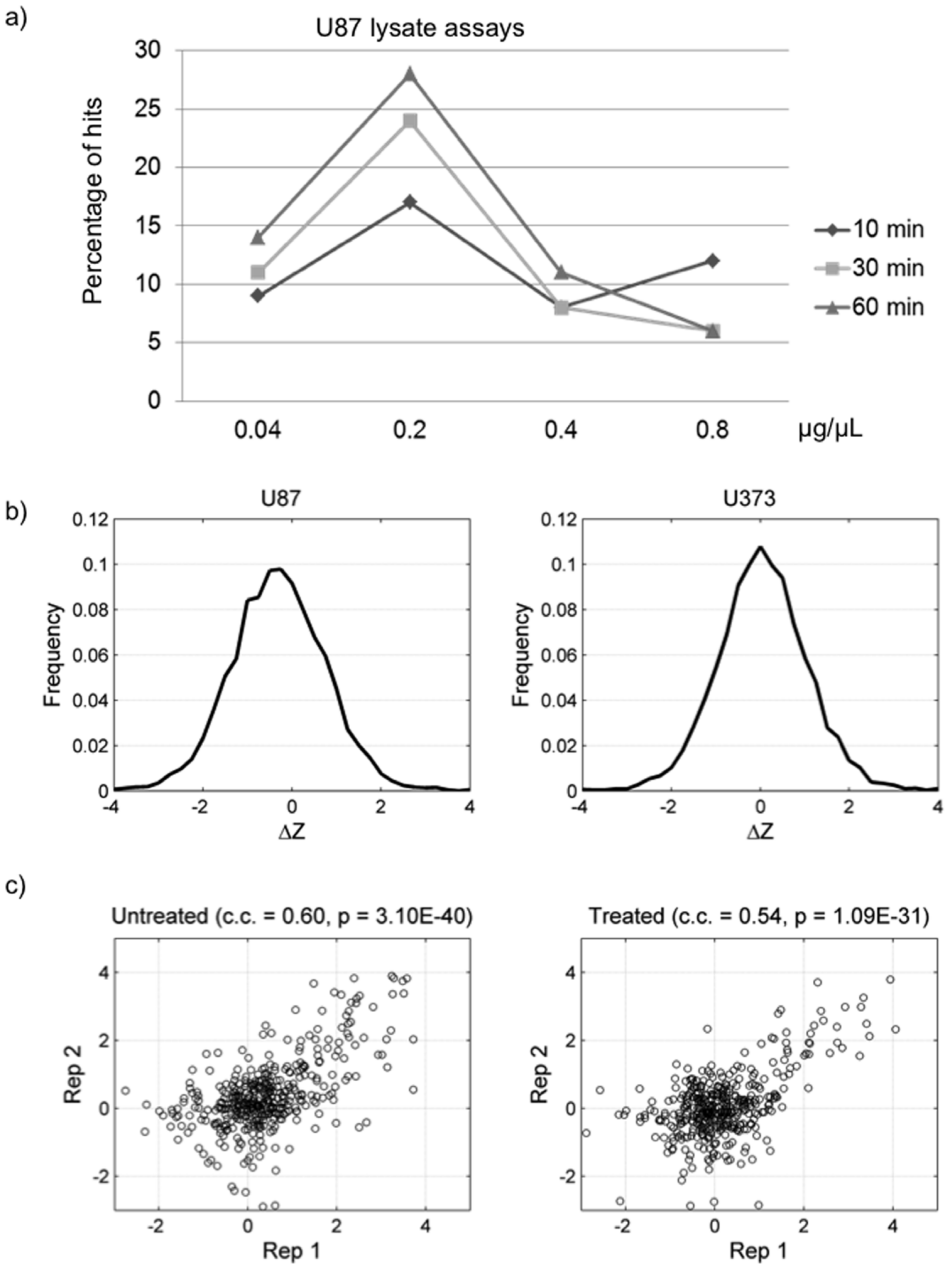
Determination of optimal lysate concentrations and incubation times using U87 cell lysates. (a) The number of positive hits increased with incubation time and peaked at 0.2 µg/µL lysates concentration. (b) Distribution of ΔZ values obtained with lysate phosphorylation reaction using U87 and U373 cells. (c) Reproducibility of protein microarray using cultured U373 HGF^−^/c-Met^+^ cell lysates in the presence or absence of HGF.

Bioinformatics analyses of the above experiments revealed that the number of phosphorylated proteins (hits) in a lysate kinase reaction increased with reaction times using lysate concentrations up to 0.2 µg/µL. The maximum number of phosphorylated proteins was detected at a total protein concentration of 0.2 µg/µL. When a higher concentration of the total proteins was used, a reduction in the phosphorylation signals and number of phosphorylated proteins on the microarrays was observed. This reduction is potentially caused by competition between lysate proteins, increased action of endogenous kinase inhibitors, or inactivation of kinases by the RIPA buffer used to lyse the cells. Since the concentration of 0.2 µg/µL yielded the highest number of hits, we compared the hits obtained from each incubation time at this concentration and observed a similar number of hits at the 30- and 60-min time points (Fig. 2). We therefore, concluded that the best reaction condition is to incubate a reaction mixture containing 0.2 µg/µL lysate proteins on a protein microarray for 30 min at 30°C. All of the following experiments were carried out under these specifications.

### Protein microarray analyses of HGF/c-Met signaling in cultured cells

**Figure 3:**
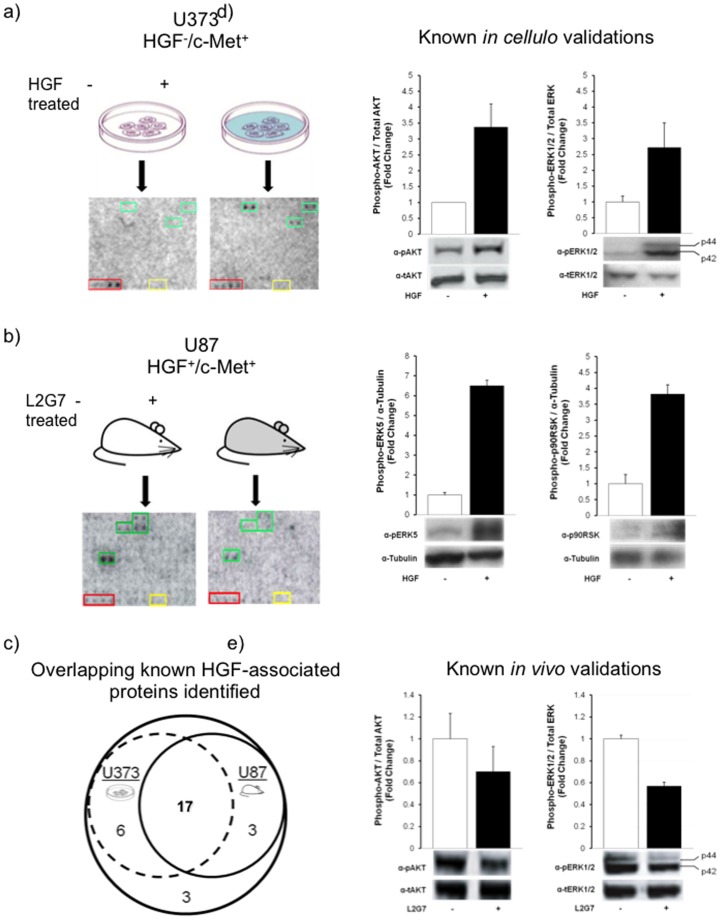
Kinase phosphorylome profiling recovered many of the known c-Met associated signaling proteins. (a,b) Schematic and representative block of the protein microarray after conducting phosphorylation assays using HGF-/Met+ U373 glioma cells and HGF+/Met+ U87 subcutaneous glioma xenografts, respectively. From left to right, top to bottom those proteins are MAPK3, HSPA9, and TSSK3 for U373 and TRAP1, MAPK3, GTF3C2, and NRP2 for U87. Phosphoprotein identified 23 and 20 of the 29 known signaling components in the HGF/c-Met signaling in *in vitro* and *in vivo* model systems, respectively. Proteins were spotted in duplicate. Red box, histones; Yellow box, BSA; Green box, differentially phosphorylated proteins. (c) Venn diagram showing substantial overlap of known hits observed on the microarray between the two model systems. (d,e) Immunoblot analyses confirming the increased phosphorylation of known HGF-associated proteins recovered from the *in vitro* and *in vivo* screens.

To demonstrate the ability of this newly established system to identify condition-specific phosphorylated proteins in cultured cells, we first asked whether the downstream mediators known to be phosphorylated upon activation of HGF/c-Met signaling could be detected using a well-characterized human glioma cell line. It is well established that c-Met stimulation by its ligand, HGF, activates a series of signaling pathways that collectively give rise to invasive growth. These pathways include Ras/Erk, PI3K/Akt, STAT, Notch, and β-catenin [32] [33] [34]. After U373 human glioma cells (c-Met^+^/HGF^−^) were treated with HGF for 30 min to activate c-Met, cell lysates were obtained and diluted to a final concentration of 0.2 µg/µL in a kinase reaction mixture. Next, phosphorylation reactions were performed on a protein microarray to obtain the phosphorylation profiles (Fig. 3a). Treatment with solute alone was used as a negative control.

To identify proteins that were differentially phosphorylated in response to HGF treatment, microarray autoradiographs were first scored using an algorithm designed to measure the relative signal intensity of each spot. *Z*-scores, representing the raw intensity of each spot normalized by the mean value and standard deviation of “empty or blank” spots on the microarray, were generated. Δ*Z* values representing *Z*-score differences between the treated and untreated cells were calculated for each protein. The distribution of Δ*Z* is shown in Figure S2a. A stringent cutoff was selected based on the maximal enrichment for phosphorylation targets known to act downstream of c-Met activation. Proteins with Δ*Z* scores above the cutoff (Δ*Z*>0.44) were defined as differentially phosphorylated in response to c-Met activation. As expected, numerous proteins were found to be differentially phosphorylated upon activation of c-Met signaling. Of the 29 proteins previously recognized to reside in the c-Met pathway and present on our microarray, 23 (79%) were found to be differentially phosphorylated following HGF treatment of U373 cells at a stringent cutoff value (p = 1.3×10^–5^, Fig. 3c and Table S1) [35]. There are several kinases involved in signaling pathways leading to cell adhesion, migration, and survival among the recovered proteins known to be associated with c-Met activation, including multiple isoforms of PKC, MEK, JNK, and AKT [32] [33] [34].

Figure 2b and 2c examine the technical reproducibility of phosphorylation microarray results. Both the untreated U373 lysate as well as HGF-simulated lysate show strong correlation between technical duplicates (correlation coefficients: 0.60 and 0.54) with significant p-values (3.10×10^–40^ and 1.09×10^–31^). Moreover, we performed the phosphorylation reactions in duplicate on the human TF protein microarrays under the optimized condition with U373 lysates (Figure S2). On the basis of analysis of the histograms of signal intensity of the duplicated reactions and with Venn diagram analysis, we confirmed that the lysate phosphorylation reactions were quite reproducible.

To further ensure that these microarray results accurately identified previously reported components of the HGF stimulated c-Met pathway, we performed immunoblot analyses to confirm the expected phosphorylation events of a subset of the known targets, namely pSer473 of AKT, pThr202 and pTyr204 of ERK1/2, pThr218/pTry220 of mitogen-activated protein kinase 7 (MAPK7/ERK5) [29], and pSer380 of 90 kDa ribosomal S6 kinases (p90RSK) [37]. Using antibodies recognizing specific phosphorylated peptides, these proteins were confirmed to be preferentially phosphorylated at the expected residues in our cell lysates after HGF treatment (Fig. 3d). For example, using antibodies against Ser473 in AKT we showed that AKT phosphorylation increased ∼3.4-fold in HGF-treated cells compared to untreated cells, while both phosphorylated forms of ERK as detected by anti-pThr202 and -pTyr204 antibodies showed a ∼2.7-fold increase in HGF stimulated cells. Additionally, upon HGF activation phosphorylation on ERK5 Thr218/Try220 showed the highest increase (∼6.5-fold change). Similarly, a ∼3.5-fold increase of phosphorylation signals was detected at p90RSK Ser380. This is interesting because activation of p90RSK via autophosphorylation at Ser380 is promoted via its upstream kinases Erk1/2, a known c-Met downstream component [39]. These results are consistent with what we obtained from the protein microarray assays, where the phosphorylation of these four proteins increased 1.8 to ∼4.6 fold (linear). Please see Fig. S3a, where we used log scale with base 2.

### Protein microarray analyses of HGF/c-Met signaling using in vivo systems

We next asked if this approach could be used to identify condition-dependent phosphorylation events for *in vivo* samples. Specifically, we asked if our platform was capable of detecting changes in the phosphorylation profile of proteins in glioma xenografts upon inhibition of c-Met signaling using a previously validated neutralizing anti-HGF monoclonal antibody (L2G7) [38]. Mice (N = 4) bearing pre-established subcutaneous U87 glioma xenografts were treated with either L2G7 or the isotype control mAb 5G8 (5 mg/kg, i.p.) every alternate day for three days. Tumors were then harvested, and whole tumor lysates (0.2 µg/µL total proteins) were applied to the protein microarrays using the same protocol as previously described (Fig. 3b).

Comparison of the signal intensities (Δ*Z*<–0.88) in the phosphorylation profiles obtained by incubating with the 5G8- and L2G7-treated lysates on the microarrays led to the identification of proteins, whose phosphorylation was abolished upon neutralizing endogenous HGF by L2G7 in mice (Table S1, column 3). Of the same 29 proteins known to associate with c-Met signaling, 20 (69%) was found to show reduced phosphorylation signals in response to c-Met inhibition by L2G7 treatment (p = 3.0×10^–3^, Fig. 3c). To confirm this result, we performed immunoblot analyses using the xenograft lysates and antibodies against the phosphorylated AKT at Ser473, and phosphorylated ERK1/2 at Thr202 and Tyr204, respectively. As expected, we observed reduction of their phosphorylated signals upon inhibition of the c-Met signaling by L2G7 in all four mice (Fig. 3e). AKT phosphorylation decreased ∼30% and ERK phosphorylation decreased ∼50% compared to control tissues. Again, comparison with the immunoblot analyses indicated that the microarray results follow the same trend observed with traditional methods (Fig. S3b).

The above protein microarray experiments using cell culture and xenograft lysates together identified 26 of the 29 (90%) proteins currently known to be associated with the c-Met signaling. Especially, the 17 of the 26 (65%) proteins that were verified by both model systems. This suggests that our approach displays high fidelity in the system for the signaling pathway examined. (Fig. 3c; overlap). These shared hits include some important downstream signaling components involved in the MEK, AKT, and PKC pathways. Another important kinase recovered by both assays is MAPK8, also known as c-Jun N-terminal kinase 1 (JNK1). This kinase has been shown to initiate events of cell cycle progression or apoptosis, depending on the cell-type and the nature of a given stimuli [39]. Current models suggest that a transient stimulation of JNK, which occurs in the presence of HGF, generally leads to cell proliferation, whereas a persistent activation is conducive to apoptosis [39] [40].

### Characterization of novel signaling components of the HGF/c-Met signaling

**Figure 4:**
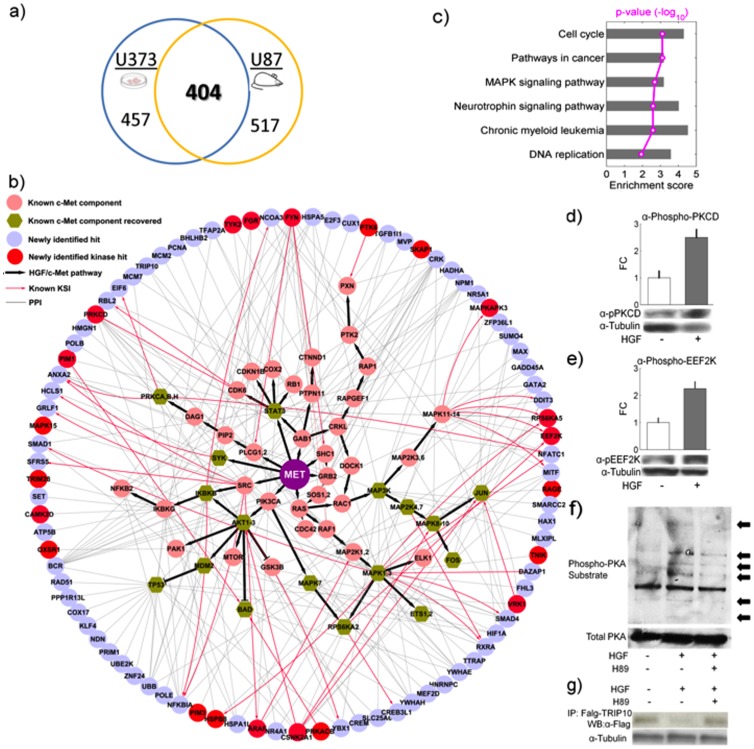
Validation of newly identified signaling components. (a) Venn diagram of number of newly identified phosphoproteins between the *in vitro* and *in vivo* systems (b) A global view of known kinase-substrate and/or protein-protein interaction between 88 newly identified candidates and c-Met signaling components. Official gene symbols were used instead of common protein names to avoid ambiguity. (c) Over-represented pathways for 88 hits in Fig. 4b. Red line denotes p-value (-log_10_). (d-e) Validation of newly identified signaling components associated with c-Met activation in HGF treated U373 HGF^−^/c-Met^+^ cells using antibodies that specifically recognize the phosphorylated forms of PKCD (pThr505), and eEF2K (pSer366), respectively. The activating phosphorylation of these kinases was elevated in comparison to untreated cells. The bar chart represents quantification analyses of the phosphorylation signals from triplicate experiments. (f) Activation of PKA catalytic subunit is also confirmed to be associated with HGF stimulation using antibodies against phosphorylated PKA substrates coupled with H89 treatment. Arrows indicate extra proteins phosphorylated by PKA upon HGF stimulation. (g) A known PKA substrate, TRIP10 was examined to confirm PKA activation. Activation of PKA upon HGF treatment results in decreased amount of TRIP10, which can be fully rescued by H89 pre-treatment in U373 HGF-/c^−^Met^+^ cells, indicating PKA-dependent effects on TRIP10.

Importantly, the ability to profile changes in the human phosphorylome in response to c-Met activation allowed us to identify additional phosphoproteins that were not previously known to be associated with the HGF/c-Met signaling. Indeed, we identified 861 and 921 preferentially phosphorylated proteins associated with c-Met activation in the cell-based and *in vivo* systems, respectively (Table S2). These two datasets also showed substantial overlap: 404 proteins (29.3%, p = 7.0×10^–5^) are shared (Fig. 4a), indicating that these proteins are likely to be influenced by HGF/c-Met signaling in cells and may be potential downstream components involved in c-Met activation by HGF.

To determine whether these newly identified candidates are relevant to the c-Met signaling, we examined the known relationships between these newly identified hits and well established HGF/c-Met associated proteins. We found that 88 of the 404 proteins are connected to the known c-Met signaling components by known protein-protein interactions and/or kinase-substrate relationships (p = 4.3×10^–2^) [2] [3] [31] (Fig. 4b). When comparing our experiments with two recently published independent studies [41] [42], we found 26 and 41 non-HGF proteins that were expressed on our phosphorylation microarrays as well. Among them, 6 and 5 proteins were within the 88 hits verified by our experiments (p = 4.1×10^–4^ and 2.2×10^–2^). This indicates a significant overlap between our results and their findings. Furthermore, bioinformatics analysis showed that these 88 proteins were significantly enriched in several KEGG pathways, including cell cycle (p = 7.83×10^–4^ after FDR multiple-test correction), neurotrophin signaling pathway (p = 2.57×10^–3^), chronic myeloid leukemia (p = 2.57×10^–3^), and DNA replication (p = 1.16×10^–2^). The proteins in this dataset are also highly enriched in cancer-related pathways (p = 7.83×10^–4^), such as the MAPK signaling pathway (p = 2.08×10^–3^) (Fig. 4c and Table S3). Interestingly, 22 of the 88 proteins themselves are kinases (red nodes in Fig. 4b) compared to 196 kinases of 2207 non-HGF proteins on our microarray (p = 3.72×10^–6^). On the basis of our study and previous reports, it is likely that activation of c-Met signaling by HGF may trigger a series of parallel kinase cascades downstream.

To determine whether any novel signaling pathways are activated in response to c-Met activation, we decided to examine phosphorylation status of these 22 kinases at specific residues that are known to modulate their activities. Limited by the availability, we applied commercially available antibodies that each recognizes pSer366 of elongation factor-2 kinase (eEF2K) [36] and pThr505 of protein kinase C-delta (PKCD) [44] in HGF-treated U373 cells (HGF^−^/c-Met^+^). Without exception, the tested kinases are substantially phosphorylated at the expected activation sites 30 min after HGF treatment (Fig. 4d–e). Intriguingly, we also observed that in the same time frame PKCdelta (PKCD) was activated via Thr505 phosphorylation (Fig. 4d), which is known to further enhance the translation elongation activity of eEF1A, another important elongation factor involved in recruiting acyl-tRNA to ribosomal A site [45]. Consistent with this observation, we observed at least 2-fold increase of phosphorylation signals at eEF2K Ser366 (Fig. 4e), a site directly phosphorylated by its upstream kinase p90RSK to inhibit eEF2K's kinase activity [43].

Another interesting kinase predicted by our microarray results to act downstream of HGF/c-Met stimulation is the protein kinase A catalytic subunit-beta (PRKACB). To determine whether the activity of PKA is enhanced in response to c-Met activation, we examined PKA-dependent phosphorylation events in cells using an antibody that recognizes phosphorylated PKA substrates [46] [47]. We observed that PKA-dependent phosphorylation was substantially enhanced upon HGF treatment, while the total protein level of PKA was unchanged (Fig. 4f). Moreover, pre-treating the cells with a PKA inhibitor H89 diminished the PKA phosphorylation signals induced by HGF, confirming that the observed phosphorylation events were PKA-dependent. Because a known PKA substrate, thyroid hormone receptor interactor 10 (TRIP10), was found differentially phosphorylated by lysates from HGF-treated cells, we further tested the effect of PKA phosphorylation on TRIP10 upon HGF treatment in cells. We found that the protein level of FLAG-tagged TRIP10 was substantially reduced upon HGF treatment in the U373 glioma cells (Fig. 4g). This result was further supported by the observation that TRIP10 protein level was rescued via a pretreatment of the cells with H89 (Fig. 4g). Because TRIP10 has been shown to play a role in cell survival/apoptosis pathways [48] [49], our results suggest that upon HGF/c-Met activation the destabilization of TRIP10 via PKA activity might serve as a means to promote cell survival.

### Network analyses of recovered phosphorylation signaling

**Figure 5:**
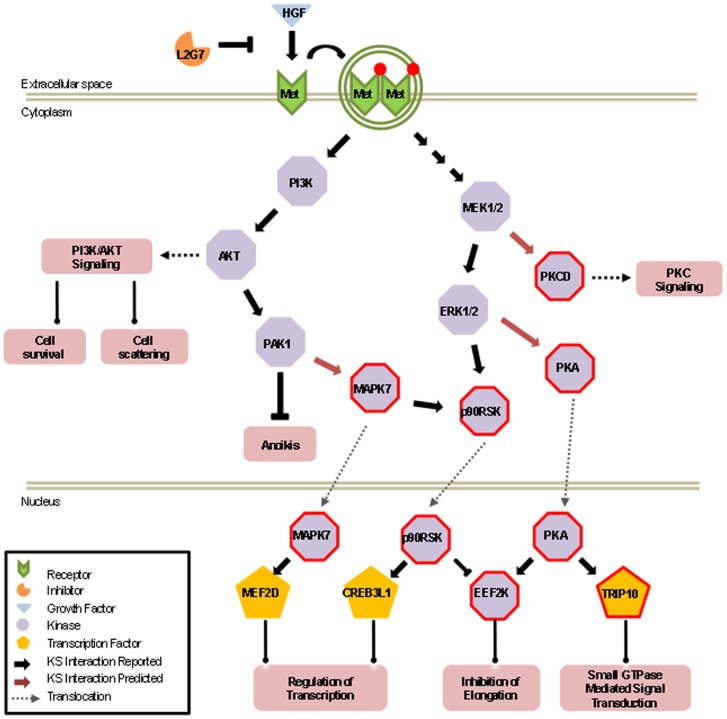
Expanded view of newly identified downstream events of HGF/c-Met phosphorylation networks in one potential cascade. All of the proteins were identified as hits on both the *in cellulo* and *in vivo* lysates phosphorylation assays. Red border indicates newly identified signaling components that have been validated to be hyper-phosphorylated downstream of HGF/c-Met signaling.

The above characterization of novel components in the HGF/c-Met signaling pathways allowed us to further extend downstream signaling events in the HGF/c-Met pathways (Fig. 5). For example, the MAPKKs (MAP2K2 and MAP2K7) and PI3K, which are previously known to be associated with c-Met signaling, play a critical role in the regulation of brain endothelial cell migration by activating the extracellular signal regulated kinases, ERK1/2, JNKs and AKT1-3 [50] [51], respectively. These downstream kinases, which were also identified by our method, can now be linked to activating three previously unassociated kinases, PKA, eEF2K, and PKCD. Because previous studies have shown that eEF2K phosphorylates one of the elongation factors, eEF2, to inhibit its activity in protein synthesis [52], our new data begins to explain the underlying mechanism of elevated protein synthesis in HGF-treated cells, in which the activated Mek/Erk signaling is likely to be further relayed via a double inhibitory p90RSK – | eEF2K – | eEF2 cascade (Fig. 5). Therefore, such an unbiased global profiling of condition-dependent phosphorylation events has the potential of connecting an upstream signaling event to downstream signaling cascades. Since activation of these kinases leads to the cellular proliferation, angiogenesis, and anti-apoptotic effects, these results now provide new insights into how HGF may affect tumor growth and angiogenesis.

## Discussion

High-throughput methodologies, such as SAGE, DNA/oligo microarrays, yeast two-hybrid, and RNA-seq that measure abundance and/or dynamic changes of entire mRNA populations in cells or tissues, have had profound impacts on modern biology. However, our ability to monitor dynamic changes in protein products is currently insufficient. For example, the 2D-gel electrophoresis is a traditional approach to profiling protein expression in a cell or tissue; however, it had only limited success, mainly due to its limitation in resolving different proteins (e.g., <4,000) and detection of PTMs. Without using the time-consuming MS/MS approach, the identity of those separated protein spots is not readily known. More importantly, it does not provide any information about the global activity of kinases. One challenging aspect is the fact that the abundance and/or dynamic changes of a given protein often involve various types of protein PTMs, such as phosphorylation, ubiquitylation, SUMOylation, acetylation, and glycosylation. These PTMs can dictate protein activity, function, fate, and subcellular localization. A high-throughput, unbiased, and cost-effective method that allows for global measurement of different types of PTMs in a given cell or tissue could have a huge impact on the study of protein dynamics. Our microarray based approach would specifically work to meet this need.

We view this method as a parallel to the DNA microarray technology. The latter uses *in vitro* hybridization on a microarray to measure mRNA abundance in cell extracts, whereas ours uses *in vitro* phosphorylation of specific substrates on a protein microarray to profile kinase activity in cell lysates. Also similar to DNA microarrays, this new method can be further exploited by profiling phosphorylomes at multiple time points and with different cell/tissue types, thus, providing a dynamic global picture of the rapid changes in kinase activity that occur both *in cellulo* and *in vivo*. This method is also potentially amenable to the measurement of other PTM enzyme families, such as ubiquitin and SUMO E3 ligases, acetyltransferases, and proteases. Further, the method is capable of examining different sample types (cell extracts, tissue lysates, biological fluids), developmental stages, and physiological states (normal versus diseased or treated versus untreated cells).

We and others have demonstrated that novel biological functions and pathways can be discovered from the use of functional protein microarrays when coupled with sophisticated bioinformatics analyses and in-depth *in vivo* characterization. This often occurs when a covalent PTM reaction is studied under highly simplified *in vitro* reaction conditions and testing a single PTM enzyme at a time. The successful examples include identification of substrates of protein kinases [2] [3] [19] [20] [53], acetyltransferases [12] [18] [54], and ubiquitin E3 ligases [17] [55]. However, these approaches risk the physiologically relevant environment, such as the context of protein complexes, adapter proteins, inhibitors, and subcellular compartmentalization. Also, such methods are not amenable to the characterization of global PTM activity. The use of cell/tissue lysates for profiling kinase activity not only allowed us to examine the dynamic changes in kinase activity as a whole, but also preserved the physiologically relevant microenvironments (i.e., in the context of protein complexes) for the kinases to execute their activity on the functional protein microarrays. Furthermore, the relevant adapters and/or required scaffold proteins are also readily available in these reactions. Indeed, using HGF/c-Met activation in cultured cells and xenograft tissues as a proof-of-principle, we were able to reproducibly recover differentially phosphorylated proteins that are known to be phosphorylated upon activation of the HGF/c-Met signaling pathway. The fidelity of the method was validated by the recovery of a high percentage of known downstream targets associated with HGF-induced cell growth, cell cycle progression, and cell survival, including AKT and MAPK. More importantly, the discovery and confirmation of novel components downstream of the HGF/c-Met signaling pathway demonstrates the usefulness of this method. Our findings underscore the complexity of the signaling events that mediate the phenotypes associated with HGF and tumorigenesis. The ability to monitor the phosphorylation of these proteins simultaneously should provide a valuable technology for network analyses and drug target discovery.

Although the use of cell lysates to discover kinase-substrate relationships has been reported previously [56], our approach allows for a more comprehensive survey of a given proteome. We are aware that this high-throughput approach has its own shortcomings, including loss of compartmentalization and competition from endogenous proteins for the kinases. To enhance the sensitivity of this method, phosphatase and protease inhibitors were added into the lysates upon lysis. While the addition of the former would theoretically increase the auto- and trans-phosphorylation activity of the kinases, such arbitrary effects can be cancelled out when condition-dependent phosphorylation events are selected. Furthermore, additional bioinformatics analyses, as exemplified in this study, helps to exclude obvious false positives. The fact that all the tested novel candidates were confirmed in cell-based assays suggests that this approach is likely to identify differentially phosphorylated proteins *in vivo*. Although the antibody arrays currently used are able to reasonably identify phosphorylated kinases in a mixture, it is not our intention to predict upstream kinases of these condition-dependent phosphorylated proteins in this study. Rather we have elucidated the global phosphorylated proteins in our selected system and envision that we will be able to use this technique to predict the corresponding upstream kinases once a more comprehensive and reliable network of human kinase-substrate relationships is available.

## Supporting Information

Figure S1 **Examples of lysate phosphorylation reactions on a human protein microarray.** (a) Representative human transcription factor protein microarray incubated with lysate. (b) Selected block of each protein microarray at different lysate concentrations and incubation times. Shown are microarrays treated with U87 cell lysate at 30°C in order to optimize reaction conditions.
(TIF)

Figure S2 **Lysate phosphorylation reaction with U373 cells performed on the human TF microarray at a final concentration of 0.25 μg/μL of total lysate proteins.** (a) Histograms of signal intensity (foreground/background) of the duplicated assays. (b) Venn Diagram of the overlapping hits identified in the duplicated assays.
(TIF)

Figure S3 **Comparison of microarray-based techniques to traditional immunoblot analysis.** (a) U373 HGF^−^/c-Met^+^ cell lysates. (b) U87 HGF^+^/c-Met^+^ cell lysates.
(TIF)

Table S1 **List of known HGF/c-Met signaling components that are available on our protein microarray.** Compiled from http://www.sabiosciences.com/pathway.php?sn=HGF and Franco, M., Muratori, C., Corso, S., Tenaglia, E., Bertotti, A., Capparuccia, L., Trusolino, L., Comoglio, P.M. and Tamagnone, L. (2010). The tetraspanin CD151 is required for Met-dependent signaling and tumor cell growth. *J Biol Chem*, **285**, 38756–38764.
(DOC)

Table S2 **List of all proteins identifed by either U373 and/or U87 lysate phosphorylation arrays excluding HGF/c-Met signaling components.**
(DOC)

Table S3 **List of 88 protein hits in pathway analysis (** **Figs. 4b and c** **).** The KEGG pathways were listed here only if their enrichment scores were larger than 3 and p-values based on hypergeometric model were less than 0.05 after false discovery rate (FDR) multiple-test correction.
(DOC)
