# Supplementary figures and images for: Profiling the Dynamics of a Human Phosphorylome Reveals New Components in HGF/c-Met Signaling
Source: PLoS One. 2013 Sep 2;8(9):e72671. doi: 10.1371/journal.pone.0072671 (PMC3759380; doi:10.1371/journal.pone.0072671)

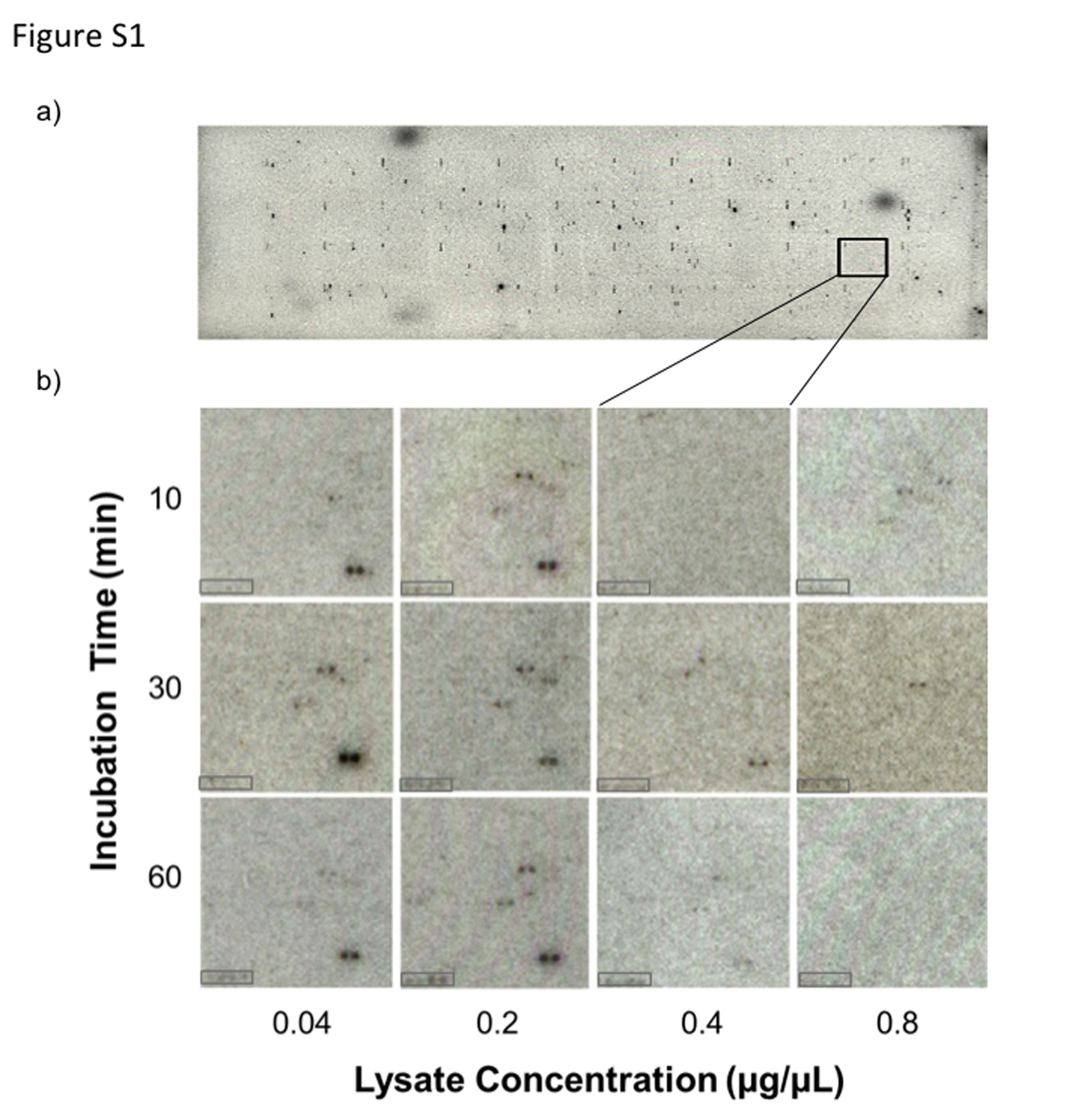

Supplement: Figure S1 — Examples of lysate phosphorylation reactions on a human protein microarray. (a) Representative human transcription factor protein microarray incubated with lysate. (b) Selected block of each protein microarray at different lysate concentrations and incubation times. Shown are microarrays treated with U87 cell lysate at 30°C in order to optimize reaction conditions. (TIF) [file pone.0072671.s001.tif]

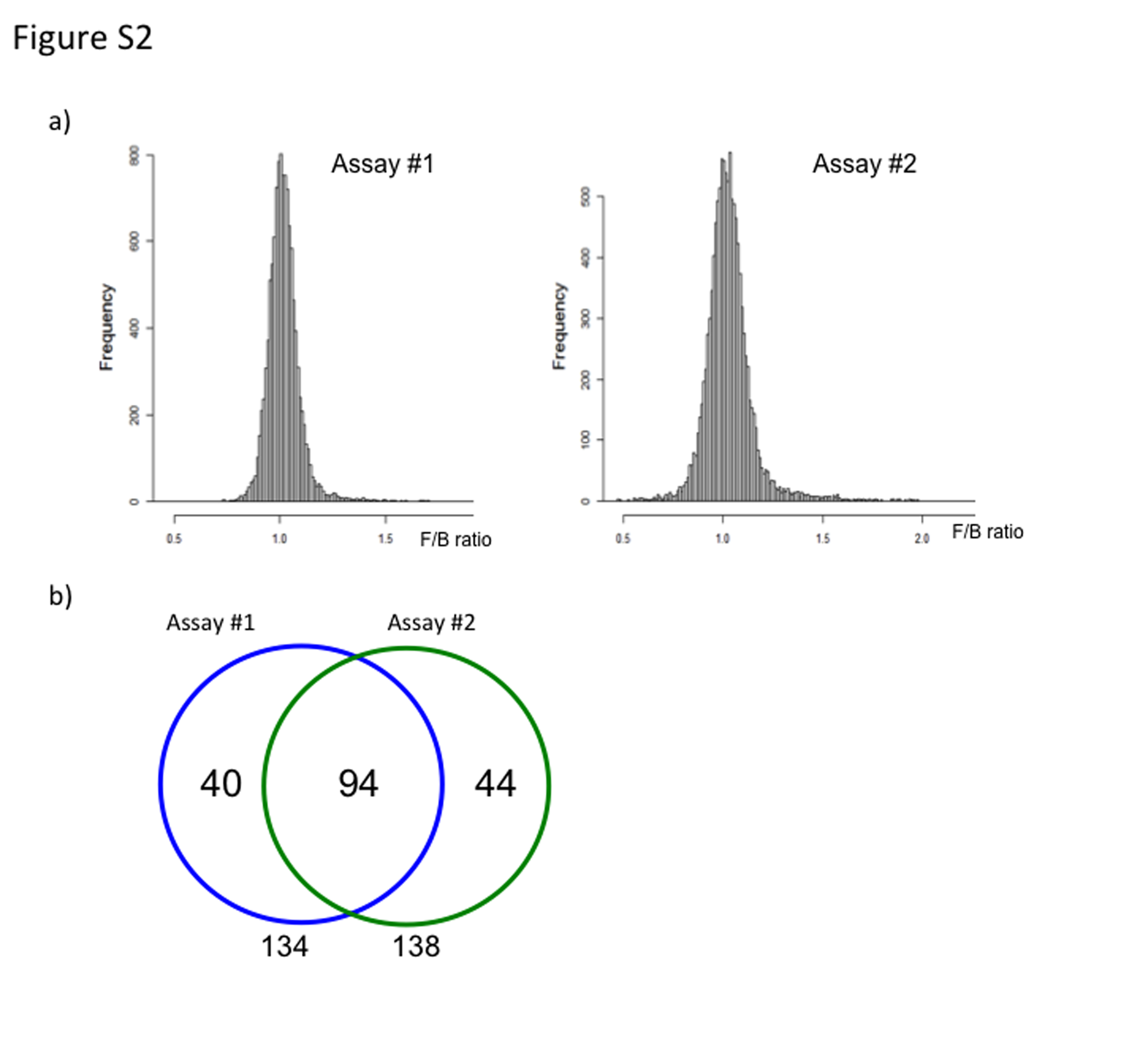

Supplement: Figure S2 — Lysate phosphorylation reaction with U373 cells performed on the human TF microarray at a final concentration of 0.25 μg/μL of total lysate proteins. (a) Histograms of signal intensity (foreground/background) of the duplicated assays. (b) Venn Diagram of the overlapping hits identified in the duplicated assays. (TIF) [file pone.0072671.s002.tif]

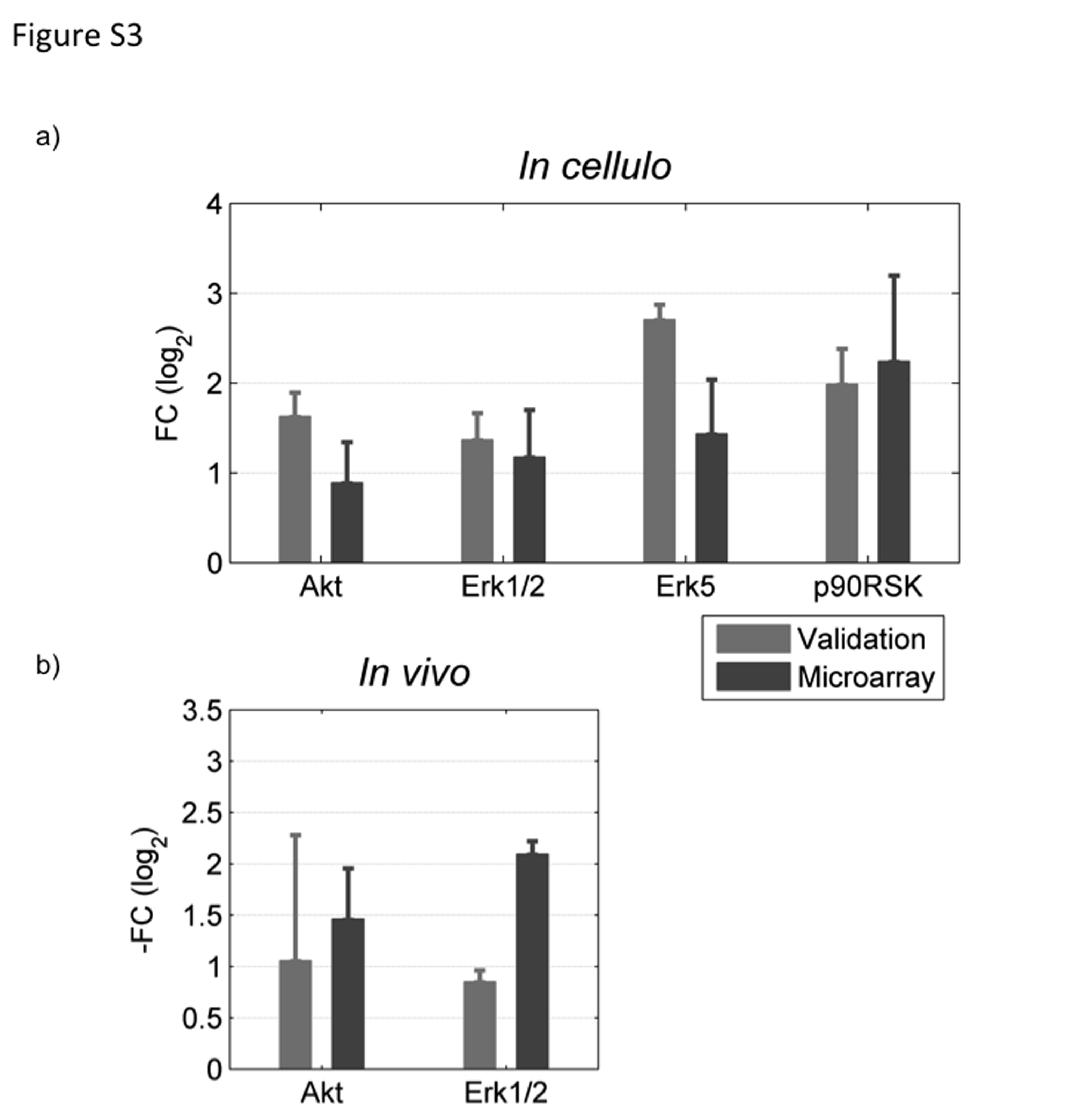

Supplement: Figure S3 — Comparison of microarray-based techniques to traditional immunoblot analysis. (a) U373 HGF−/c-Met+ cell lysates. (b) U87 HGF+/c-Met+ cell lysates. (TIF) [file pone.0072671.s003.tif]
