# Supplementary material for: Profiling the Dynamics of a Human Phosphorylome Reveals New Components in HGF/c-Met Signaling
Source: PLoS One. 2013 Sep 2;8(9):e72671. doi: 10.1371/journal.pone.0072671 (PMC3759380; doi:10.1371/journal.pone.0072671)
Supplement: Table S1 — List of known HGF/c-Met signaling components that are available on our protein microarray. Compiled from http://www.sabiosciences.com/pathway.php?sn=HGF and Franco, M., Muratori, C., Corso, S., Tenaglia, E., Bertotti, A., Capparuccia, L., Trusolino, L., Comoglio, P.M. and Tamagnone, L. (2010). The tetraspanin CD151 is required for Met-dependent signaling and tumor cell growth. J Biol Chem, 285, 38756–38764. (DOC) [file pone.0072671.s004.doc]

**Supplemental Table 1 | List of known HGF/c-Met signaling components that are available on our protein microarray.** Compiled from <http://www.sabiosciences.com/pathway.php?sn=HGF> and Franco, M., Muratori, C., Corso, S., Tenaglia, E., Bertotti, A., Capparuccia, L., Trusolino, L., Comoglio, P.M. and Tamagnone, L. (2010). The tetraspanin CD151 is required for Met-dependent signaling and tumor cell growth. *J Biol Chem*, **285**, 38756-38764.

| **Pathway Names** | **Gene Symbol** | **U87** | | | | **U373** | | | |
| --- | --- | --- | --- | --- | --- | --- | --- | --- | --- |
|  |  | Untreated | Treated | ΔZ | hit | Untreated | Treated | ΔZ | hit |
| Akt | AKT1 | 2.33 | 0.65 | -1.68 | Y | -1.07 | -0.36 | 0.70 | Y |
|  | AKT2 | 0.90 | -0.63 | -1.53 | Y | -0.61 | 0.06 | 0.67 | Y |
|  | AKT3 | 0.92 | -0.27 | -1.18 | Y | -0.61 | 0.68 | 1.30 | Y |
| Bad | BAD | 0.86 | -0.30 | -1.16 | Y | -0.65 | 0.73 | 1.38 | Y |
| c-Fos | FOS | 0.39 | -1.10 | -1.50 | Y | -1.22 | -0.48 | 0.74 | Y |
|  | FOSB | 1.41 | 0.41 | -1.01 | Y | -0.31 | 1.24 | 1.56 | Y |
| ERK1/2 | MAPK3 | 1.02 | -1.07 | -2.10 | Y | 0.00 | 1.18 | 1.18 | Y |
|  | MAPK1 | 1.12 | 0.43 | -0.69 | - | 0.74 | 0.12 | -0.62 | - |
| ERK5 | MAPK7 | 2.94 | 0.40 | -2.54 | Y | 0.53 | 1.97 | 1.43 | Y |
| Ets | ETS1 | 0.06 | -1.18 | -1.24 | Y | 1.44 | 1.89 | 0.45 | Y |
|  | ETS2 | 2.35 | 0.41 | -1.94 | Y | 0.36 | 0.93 | 0.56 | Y |
| IKK | IKBKB | 0.94 | -0.57 | -1.51 | Y | 1.18 | 1.83 | 0.65 | Y |
| JNK | MAPK8 | 2.70 | 0.49 | -2.21 | Y | -0.24 | 1.59 | 1.83 | Y |
|  | MAPK9 | 1.61 | -0.16 | -1.78 | Y | 0.43 | 2.27 | 1.84 | Y |
|  | MAPK10 | 2.21 | 0.71 | -1.50 | Y | 0.17 | 1.14 | 0.97 | Y |
| MDM2 | MDM2 | 0.73 | -0.70 | -1.43 | Y | -0.09 | 0.49 | 0.58 | Y |
| MEK4/7 | MAP2K4 | 0.89 | 0.27 | -0.62 | - | 0.27 | 0.28 | 0.02 | - |
|  | MAP2K7 | 0.49 | -0.48 | -0.97 | Y | -0.53 | 0.84 | 1.37 | Y |
| MEKK | MAP3K3 | 0.00 | -0.05 | -0.05 | - | -0.38 | -0.86 | -0.48 | - |
|  | MAP3K5 | -0.28 | 1.10 | 1.38 | - | -0.63 | 1.09 | 1.72 | Y |
|  | MAP3K7 | 0.76 | -0.32 | -1.08 | Y | -0.57 | -0.75 | -0.18 | - |
|  | MAP3K8 | 0.17 | -0.94 | -1.12 | Y | 0.82 | 0.99 | 0.18 | - |
|  | MAP3K11 | 0.00 | 1.07 | 1.06 | - | 1.30 | 0.51 | -0.79 | - |
|  | MAP3K13 | 2.36 | 0.51 | -1.85 | Y | 0.36 | -0.14 | -0.50 | - |
|  | MAP3K14 | -1.20 | 0.15 | 1.35 | - | 0.11 | -0.49 | -0.60 | - |
| p53 | TP53 | 1.71 | -0.42 | -2.13 | Y | -0.28 | 0.29 | 0.57 | Y |
| p90RSK | RPS6KA2 | 2.31 | -0.30 | -2.62 | Y | 0.04 | 2.29 | 2.24 | Y |
| PKC α,β,γ | PRKCA | 0.50 | 0.81 | 0.31 | - | 0.06 | 0.53 | 0.46 | Y |
|  | PRKCB | 0.25 | -0.54 | -0.79 | - | -0.04 | 0.44 | 0.48 | Y |
|  | PRKCH | 0.98 | -1.03 | -2.01 | Y | -0.48 | 0.13 | 0.61 | Y |
| STAT3 | STAT3 | 1.90 | 0.29 | -1.61 | Y | 0.07 | 0.65 | 0.58 | Y |
| c-Jun | JUN | -0.12 | -1.02 | -0.90 | Y | -0.54 | -0.60 | -0.06 | - |
|  | JUNB | -0.25 | 1.12 | 1.37 | - | 0.23 | 1.45 | 1.22 | Y |
| SYK | SYK | -0.26 | -1.17 | -0.90 | Y | -0.55 | 0.79 | 1.34 | Y |
| CDK6 | CDK6 | 1.10 | -0.66 | -1.76 | Y | 0.30 | -0.54 | -0.84 | - |
| CrkL | CRKL | -1.66 | 0.23 | 1.89 | - | -0.44 | 1.15 | 1.59 | Y |
| Elk1 | ELK1 | 2.02 | 0.56 | -1.46 | Y | 0.49 | 0.18 | -0.31 | - |
| FAK | PTK2 | 0.08 | 1.83 | 1.76 | - | 1.25 | 2.26 | 1.01 | Y |
| GSK3β | GSK3B | 1.02 | 0.36 | -0.66 | - | 1.44 | 0.25 | -1.19 | - |
| MEK1/2 | MAP2K1 | 0.28 | 0.31 | 0.03 | - | 1.02 | -0.83 | -1.85 | - |
|  | MAP2K2 | 1.80 | 1.51 | -0.29 | - | -0.61 | 1.37 | 1.98 | Y |
| MEK3/6 | MAP2K3 | 0.26 | 0.02 | -0.24 | - | 0.93 | 0.13 | -0.80 | - |
|  | MAP2K6 | -1.30 | -0.69 | 0.60 | - | -0.85 | -0.48 | 0.37 | - |
| PAK1 | PAK1 | 1.41 | 0.52 | -0.89 | Y | 0.42 | 0.59 | 0.18 | - |
| p38α,β,γ,δ | MAPK14 | 0.28 | -0.02 | -0.30 | - | -0.27 | -0.06 | 0.21 | - |
|  | MAPK11 | -0.31 | 0.44 | 0.76 | - | 0.19 | -0.19 | -0.38 | - |
|  | MAPK12 | -0.14 | 0.97 | 1.10 | - | -0.24 | 2.70 | 2.95 | Y |
|  | MAPK13 | 0.62 | 1.06 | 0.44 | - | -0.91 | -0.34 | 0.57 | Y |
| PI3K | PIK3C3 | -0.62 | -0.91 | -0.29 | - | -1.33 | 0.15 | 1.48 | Y |
|  | PIK3R4 | -0.52 | -1.10 | -0.57 | - | -0.70 | 0.25 | 0.95 | Y |
| Raf1 | RAF1 | 0.98 | 0.50 | -0.48 | - | -0.05 | -0.50 | -0.45 | - |
| Src | SRC | 0.72 | 0.88 | 0.17 | - | 1.75 | 3.97 | 2.22 | Y |
