# Supplementary material for: Profiling the Dynamics of a Human Phosphorylome Reveals New Components in HGF/c-Met Signaling
Source: PLoS One. 2013 Sep 2;8(9):e72671. doi: 10.1371/journal.pone.0072671 (PMC3759380; doi:10.1371/journal.pone.0072671)
Supplement: Table S2 — List of all proteins identifed by either U373 and/or U87 lysate phosphorylation arrays excluding HGF/c-Met signaling components. (DOC) [file pone.0072671.s005.doc]

**Supplemental Table 2 | List of all proteins identifed by either U373 and/or U87 lysate phosphorylation arrays excluding HGF/c-Met signaling components.**

|  | **U87** | | | | **U373** | | | |
| --- | --- | --- | --- | --- | --- | --- | --- | --- |
| **Gene Symbol** | Untreated | Treated | ΔZ | hit | Untreated | Treated | ΔZ | hit |
| CSNK1G2 | 3.30 | 0.15 | -3.15 | Y | 0.87 | 7.18 | 6.32 | Y |
| SSRP1 | 4.55 | 0.80 | -3.75 | Y | 0.08 | 3.89 | 3.81 | Y |
| WHSC1 | 1.35 | -0.37 | -1.72 | Y | -0.72 | 2.86 | 3.58 | Y |
| SRPK2 | 4.51 | 1.78 | -2.73 | Y | 2.23 | 5.72 | 3.49 | Y |
| TTBK2 | 3.57 | 1.71 | -1.87 | Y | -0.23 | 3.16 | 3.38 | Y |
| SFRS6 | 2.48 | 0.82 | -1.66 | Y | 0.68 | 3.81 | 3.14 | Y |
| RBM42 | 1.25 | -0.52 | -1.77 | Y | -0.72 | 2.39 | 3.10 | Y |
| SMARCC2 | 2.24 | 0.64 | -1.60 | Y | -0.86 | 2.20 | 3.05 | Y |
| PHF20L1 | 1.75 | -0.48 | -2.23 | Y | -0.55 | 2.46 | 3.02 | Y |
| CELF5 | 1.77 | -0.39 | -2.16 | Y | -0.53 | 2.33 | 2.86 | Y |
| MAGEA9 | 1.93 | -0.89 | -2.82 | Y | -0.71 | 2.14 | 2.85 | Y |
| NFATC1 | 4.20 | 2.76 | -1.44 | Y | 0.87 | 3.71 | 2.84 | Y |
| KIAA0907 | 1.82 | 0.60 | -1.22 | Y | -0.31 | 2.49 | 2.80 | Y |
| IRAK4 | 5.06 | 3.30 | -1.76 | Y | 2.74 | 5.53 | 2.79 | Y |
| MAPKAPK3 | 1.84 | 0.91 | -0.93 | Y | -0.59 | 2.17 | 2.77 | Y |
| SCAPER | 2.33 | 0.11 | -2.22 | Y | -0.77 | 1.99 | 2.77 | Y |
| GTF2A2 | 2.59 | 0.18 | -2.42 | Y | -0.87 | 1.86 | 2.73 | Y |
| SRPK1 | 3.48 | 1.65 | -1.84 | Y | -0.83 | 1.86 | 2.69 | Y |
| E2F3 | 0.83 | -0.42 | -1.25 | Y | -0.66 | 2.02 | 2.68 | Y |
| FARS2 | 1.07 | -0.64 | -1.71 | Y | -1.08 | 1.58 | 2.66 | Y |
| UBXN1 | 1.03 | -0.27 | -1.29 | Y | -0.90 | 1.75 | 2.65 | Y |
| PPIL4 | 1.79 | -0.64 | -2.43 | Y | -0.17 | 2.43 | 2.60 | Y |
| ZDHHC12 | 0.75 | -0.36 | -1.11 | Y | -0.98 | 1.61 | 2.59 | Y |
| PHACTR3 | 1.86 | -0.39 | -2.25 | Y | -0.57 | 1.97 | 2.54 | Y |
| DNAJB2 | 1.60 | 0.39 | -1.21 | Y | -0.88 | 1.57 | 2.45 | Y |
| ZNF483 | 2.88 | 0.25 | -2.63 | Y | -1.03 | 1.36 | 2.39 | Y |
| CREM | 2.22 | 0.49 | -1.74 | Y | -0.72 | 1.65 | 2.37 | Y |
| BCL11A | 1.31 | 0.29 | -1.02 | Y | -0.38 | 1.96 | 2.34 | Y |
| TRIP10 | 1.15 | -0.19 | -1.34 | Y | -1.17 | 1.17 | 2.34 | Y |
| PKNOX2 | 2.25 | -0.65 | -2.91 | Y | 0.37 | 2.69 | 2.33 | Y |
| NBPF15 | 1.84 | -0.82 | -2.66 | Y | -0.39 | 1.90 | 2.29 | Y |
| ZNF24 | 0.16 | -1.21 | -1.37 | Y | -1.18 | 1.09 | 2.27 | Y |
| MYF6 | 1.04 | -0.69 | -1.72 | Y | -1.16 | 1.09 | 2.25 | Y |
| EIF4A2 | 1.42 | 0.07 | -1.36 | Y | -0.93 | 1.32 | 2.24 | Y |
| RNF13 | 1.26 | -0.76 | -2.02 | Y | -1.49 | 0.75 | 2.24 | Y |
| RAD51L3 | 0.53 | -0.77 | -1.30 | Y | -0.97 | 1.25 | 2.22 | Y |
| PTCD1 | 1.42 | 0.16 | -1.27 | Y | -0.66 | 1.55 | 2.21 | Y |
| EIF6 | 0.62 | -0.76 | -1.38 | Y | -0.62 | 1.58 | 2.20 | Y |
| CCNT1 | 1.08 | 0.20 | -0.88 | Y | -0.47 | 1.72 | 2.19 | Y |
| TERF1 | 3.77 | 0.65 | -3.12 | Y | 1.05 | 3.23 | 2.18 | Y |
| SAFB2 | 2.11 | -0.93 | -3.05 | Y | 0.26 | 2.43 | 2.17 | Y |
| RXRA | 0.57 | -0.45 | -1.02 | Y | -0.84 | 1.32 | 2.16 | Y |
| GTPBP1 | 2.92 | -0.61 | -3.54 | Y | -0.05 | 2.10 | 2.15 | Y |
| LOC653349 | 1.31 | 0.19 | -1.11 | Y | -0.83 | 1.30 | 2.13 | Y |
| ACADVL | 2.58 | -0.85 | -3.43 | Y | -1.16 | 0.97 | 2.12 | Y |
| DENR | 2.26 | 0.70 | -1.56 | Y | -0.14 | 1.97 | 2.11 | Y |
| OXSR1 | 2.36 | 0.52 | -1.85 | Y | -0.06 | 2.05 | 2.11 | Y |
| SHMT2 | 1.43 | 0.45 | -0.98 | Y | -0.10 | 1.97 | 2.08 | Y |
| ATP5B | 0.80 | -0.37 | -1.17 | Y | 0.19 | 2.23 | 2.04 | Y |
| MITF | 1.00 | -1.28 | -2.28 | Y | -1.04 | 1.00 | 2.04 | Y |
| DDX11 | 0.88 | -0.47 | -1.35 | Y | -0.78 | 1.24 | 2.02 | Y |
| TLE4 | 1.80 | 0.41 | -1.39 | Y | -0.64 | 1.36 | 2.00 | Y |
| RPS6KL1 | 1.78 | 0.13 | -1.65 | Y | -0.04 | 1.89 | 1.94 | Y |
| PRKACB | 1.94 | 0.59 | -1.35 | Y | 0.71 | 2.63 | 1.93 | Y |
| RIMS3 | 1.52 | 0.00 | -1.52 | Y | -0.04 | 1.87 | 1.91 | Y |
| SMAD1 | 1.12 | -0.02 | -1.13 | Y | -0.48 | 1.40 | 1.88 | Y |
| HOXC4 | 2.64 | 1.07 | -1.57 | Y | -0.74 | 1.12 | 1.87 | Y |
| LIG3 | 0.72 | -0.83 | -1.55 | Y | -0.03 | 1.84 | 1.87 | Y |
| TRAIP | 1.07 | -0.07 | -1.14 | Y | -0.52 | 1.34 | 1.86 | Y |
| TLE2 | 0.47 | -0.85 | -1.32 | Y | 0.38 | 2.24 | 1.86 | Y |
| SFRS4 | 2.12 | 0.21 | -1.91 | Y | -0.17 | 1.69 | 1.85 | Y |
| CAMKK2 | 4.09 | 2.47 | -1.63 | Y | -0.20 | 1.65 | 1.85 | Y |
| PHKG2 | 2.21 | 0.98 | -1.23 | Y | 2.84 | 4.68 | 1.84 | Y |
| ZNF44 | 1.59 | -0.19 | -1.78 | Y | -0.81 | 1.02 | 1.83 | Y |
| MTIF3 | 1.48 | 0.47 | -1.01 | Y | -0.73 | 1.08 | 1.81 | Y |
| KIF12 | 1.91 | -0.17 | -2.08 | Y | -0.39 | 1.42 | 1.81 | Y |
| TBC1D2 | 1.30 | -0.37 | -1.66 | Y | -0.99 | 0.82 | 1.81 | Y |
| NR4A1 | 1.34 | -0.62 | -1.95 | Y | -0.53 | 1.26 | 1.79 | Y |
| LZTFL1 | 2.25 | 0.52 | -1.73 | Y | -0.65 | 1.13 | 1.79 | Y |
| STRADA | 0.80 | -0.76 | -1.56 | Y | -0.42 | 1.36 | 1.78 | Y |
| DDIT3 | 1.86 | 0.67 | -1.19 | Y | -0.44 | 1.34 | 1.78 | Y |
| PRKCD | 1.18 | -0.33 | -1.51 | Y | -0.54 | 1.24 | 1.78 | Y |
| ERCC6L | 0.74 | -0.16 | -0.90 | Y | -0.67 | 1.08 | 1.75 | Y |
| NME1 | 5.93 | 4.71 | -1.22 | Y | 0.54 | 2.25 | 1.72 | Y |
| ACTL6A | 0.89 | -0.50 | -1.38 | Y | -1.09 | 0.62 | 1.71 | Y |
| TRIM36 | 0.85 | -0.32 | -1.17 | Y | -0.62 | 1.07 | 1.69 | Y |
| TMEM65 | 0.45 | -0.50 | -0.95 | Y | -0.82 | 0.88 | 1.69 | Y |
| HNRNPC | 2.08 | 0.09 | -2.00 | Y | -1.34 | 0.35 | 1.69 | Y |
| SSX3 | 0.98 | -0.65 | -1.62 | Y | -0.92 | 0.77 | 1.69 | Y |
| BCKDK | 2.26 | -0.51 | -2.77 | Y | -0.93 | 0.75 | 1.69 | Y |
| STYK1 | 3.31 | 0.08 | -3.24 | Y | 0.32 | 2.00 | 1.68 | Y |
| NRBP1 | 1.79 | -0.27 | -2.06 | Y | -0.28 | 1.39 | 1.67 | Y |
| MTX2 | 0.22 | -1.14 | -1.36 | Y | -0.63 | 1.03 | 1.66 | Y |
| NDUFS6 | 0.20 | -1.40 | -1.60 | Y | -0.69 | 0.96 | 1.65 | Y |
| EED | 1.16 | -0.47 | -1.62 | Y | -0.27 | 1.39 | 1.65 | Y |
| PKMYT1 | 2.20 | 0.83 | -1.37 | Y | 0.43 | 2.08 | 1.65 | Y |
| HSPA5 | 1.04 | -0.27 | -1.31 | Y | 1.27 | 2.92 | 1.65 | Y |
| BDH1 | 1.36 | -0.43 | -1.80 | Y | 0.31 | 1.96 | 1.65 | Y |
| BATF3 | 1.47 | 0.09 | -1.38 | Y | 0.04 | 1.68 | 1.64 | Y |
| HIF1A | 1.80 | 0.56 | -1.24 | Y | -0.72 | 0.91 | 1.63 | Y |
| ZNF498 | 2.58 | 0.19 | -2.39 | Y | -0.45 | 1.16 | 1.62 | Y |
| ZBTB46 | 1.02 | -0.22 | -1.24 | Y | -0.50 | 1.12 | 1.61 | Y |
| TULP4 | 0.55 | -0.93 | -1.48 | Y | -0.19 | 1.42 | 1.61 | Y |
| CDK16 | 2.35 | 0.57 | -1.78 | Y | -0.53 | 1.08 | 1.61 | Y |
| NR1H2 | 1.79 | 0.21 | -1.57 | Y | 0.12 | 1.72 | 1.60 | Y |
| EEF2K | 3.05 | -0.15 | -3.20 | Y | -0.88 | 0.71 | 1.59 | Y |
| TTRAP | 1.62 | 0.16 | -1.46 | Y | -1.28 | 0.31 | 1.59 | Y |
| QKI | 1.01 | -0.32 | -1.33 | Y | -0.57 | 1.02 | 1.58 | Y |
| YEATS4 | 1.23 | -0.15 | -1.37 | Y | -0.46 | 1.11 | 1.57 | Y |
| RAGE | 1.33 | -0.81 | -2.15 | Y | -0.76 | 0.81 | 1.57 | Y |
| BTF3 | 1.42 | 0.24 | -1.18 | Y | -0.63 | 0.93 | 1.57 | Y |
| BZW1 | 0.88 | -0.20 | -1.08 | Y | -0.48 | 1.08 | 1.56 | Y |
| ASCC1 | 0.90 | -0.42 | -1.32 | Y | -0.31 | 1.25 | 1.56 | Y |
| DHX8 | 1.86 | -0.43 | -2.29 | Y | 0.44 | 1.99 | 1.56 | Y |
| OBFC1 | 1.27 | -0.40 | -1.67 | Y | -0.29 | 1.25 | 1.54 | Y |
| UBB | 0.57 | -0.93 | -1.51 | Y | -0.90 | 0.63 | 1.53 | Y |
| TUFM | 1.97 | -0.12 | -2.10 | Y | -0.63 | 0.90 | 1.53 | Y |
| SARNP | 0.73 | -0.57 | -1.29 | Y | -0.78 | 0.76 | 1.53 | Y |
| MAPK15 | 0.55 | -0.71 | -1.27 | Y | -0.44 | 1.07 | 1.51 | Y |
| SMCR7 | 0.82 | -0.25 | -1.07 | Y | -0.38 | 1.13 | 1.51 | Y |
| SMPX | 1.67 | 0.17 | -1.50 | Y | -0.43 | 1.08 | 1.50 | Y |
| LUZP2 | 2.58 | -0.31 | -2.89 | Y | -0.65 | 0.85 | 1.50 | Y |
| ODC1 | 0.93 | -0.33 | -1.27 | Y | -0.56 | 0.94 | 1.50 | Y |
| HSPA1L | 1.64 | -0.62 | -2.26 | Y | -1.35 | 0.14 | 1.49 | Y |
| HDAC7 | 0.93 | -0.24 | -1.17 | Y | -0.66 | 0.80 | 1.46 | Y |
| ARC | 1.84 | 0.21 | -1.63 | Y | -1.06 | 0.40 | 1.46 | Y |
| MAX | 2.66 | 0.62 | -2.04 | Y | 0.59 | 2.05 | 1.46 | Y |
| NME2 | 2.36 | 0.58 | -1.78 | Y | 1.92 | 3.36 | 1.44 | Y |
| ZNF706 | 2.13 | 0.64 | -1.49 | Y | 0.50 | 1.94 | 1.44 | Y |
| SAP30BP | 1.83 | 0.09 | -1.74 | Y | 0.22 | 1.66 | 1.44 | Y |
| NR5A1 | 0.54 | -0.37 | -0.91 | Y | -0.70 | 0.74 | 1.43 | Y |
| YBX1 | 1.01 | -0.58 | -1.59 | Y | -1.32 | 0.11 | 1.43 | Y |
| TFAP2A | 1.33 | -0.60 | -1.92 | Y | 0.00 | 1.43 | 1.43 | Y |
| MRPL2 | 0.57 | -0.79 | -1.36 | Y | -1.38 | 0.04 | 1.42 | Y |
| NRBF2 | 1.56 | 0.41 | -1.16 | Y | -0.67 | 0.74 | 1.41 | Y |
| MEOX1 | 1.30 | -0.67 | -1.98 | Y | -1.20 | 0.20 | 1.40 | Y |
| PLP2 | 1.61 | 0.72 | -0.89 | Y | -0.66 | 0.73 | 1.40 | Y |
| MAGEB6 | 1.86 | -0.24 | -2.10 | Y | -0.55 | 0.84 | 1.39 | Y |
| DUS3L | 0.57 | -0.61 | -1.19 | Y | -0.08 | 1.29 | 1.37 | Y |
| CDC40 | 1.19 | 0.20 | -0.99 | Y | -0.70 | 0.68 | 1.37 | Y |
| SKAP1 | 1.84 | 0.84 | -1.00 | Y | 0.62 | 1.99 | 1.36 | Y |
| DGCR8 | 1.76 | 0.29 | -1.47 | Y | -0.08 | 1.29 | 1.36 | Y |
| GFM2 | 2.11 | -1.10 | -3.21 | Y | -0.26 | 1.09 | 1.35 | Y |
| NAP1L3 | 0.51 | -0.76 | -1.26 | Y | 0.60 | 1.94 | 1.35 | Y |
| PRDM9 | 1.23 | -0.39 | -1.63 | Y | -0.47 | 0.86 | 1.34 | Y |
| LARP4 | -0.11 | -1.06 | -0.95 | Y | 0.18 | 1.51 | 1.33 | Y |
| P4HB | 0.66 | -0.84 | -1.50 | Y | 0.12 | 1.45 | 1.33 | Y |
| UQCRFS1 | 0.29 | -1.37 | -1.65 | Y | 0.29 | 1.62 | 1.33 | Y |
| RMI1 | 1.19 | 0.22 | -0.97 | Y | -0.44 | 0.88 | 1.33 | Y |
| TCEAL6 | 1.64 | -0.42 | -2.06 | Y | -0.57 | 0.75 | 1.32 | Y |
| POU3F3 | 1.01 | -0.59 | -1.60 | Y | -0.19 | 1.13 | 1.32 | Y |
| NDN | 2.57 | 0.11 | -2.46 | Y | -0.45 | 0.86 | 1.32 | Y |
| BAMBI | 1.07 | -0.40 | -1.47 | Y | -0.56 | 0.74 | 1.30 | Y |
| COX17 | 1.06 | -0.45 | -1.51 | Y | -0.02 | 1.28 | 1.30 | Y |
| NUDT21 | 1.11 | 0.14 | -0.97 | Y | -0.88 | 0.42 | 1.29 | Y |
| TGIF1 | 0.96 | -0.44 | -1.40 | Y | 0.37 | 1.66 | 1.29 | Y |
| OPA3 | 0.87 | -0.14 | -1.01 | Y | -0.57 | 0.72 | 1.29 | Y |
| HNRNPD | 1.53 | 0.06 | -1.47 | Y | -0.42 | 0.87 | 1.29 | Y |
| TLE3 | 1.43 | -1.27 | -2.70 | Y | -0.33 | 0.96 | 1.29 | Y |
| KCNIP2 | 1.70 | 0.32 | -1.38 | Y | -0.79 | 0.49 | 1.28 | Y |
| TOE1 | 1.90 | -0.24 | -2.14 | Y | -0.15 | 1.13 | 1.28 | Y |
| RPS6KA5 | 0.80 | -0.97 | -1.77 | Y | -0.28 | 0.99 | 1.27 | Y |
| ZNF165 | 1.26 | -0.19 | -1.45 | Y | -0.36 | 0.91 | 1.27 | Y |
| FGR | 2.62 | -0.02 | -2.64 | Y | 1.50 | 2.76 | 1.26 | Y |
| CSAD | 1.56 | -0.08 | -1.64 | Y | 0.11 | 1.37 | 1.26 | Y |
| HPCAL1 | 1.43 | 0.43 | -1.00 | Y | -0.47 | 0.78 | 1.26 | Y |
| VRK1 | 2.32 | 0.58 | -1.74 | Y | 0.13 | 1.39 | 1.25 | Y |
| GSPT1 | 2.94 | 0.83 | -2.11 | Y | 0.01 | 1.26 | 1.25 | Y |
| PTRH2 | 1.13 | -0.86 | -1.99 | Y | -1.13 | 0.11 | 1.24 | Y |
| GABPB1 | 1.95 | -0.77 | -2.72 | Y | -1.08 | 0.15 | 1.24 | Y |
| ZRANB2 | 1.80 | -0.18 | -1.98 | Y | -0.69 | 0.54 | 1.23 | Y |
| MUT | 0.34 | -1.59 | -1.93 | Y | -0.65 | 0.58 | 1.23 | Y |
| KBTBD7 | 4.35 | 0.95 | -3.41 | Y | -0.59 | 0.63 | 1.22 | Y |
| MCM2 | 1.02 | -0.36 | -1.38 | Y | -0.94 | 0.27 | 1.21 | Y |
| SLC25A6 | 1.14 | 0.08 | -1.06 | Y | -1.02 | 0.19 | 1.21 | Y |
| GATA2 | 1.02 | -0.16 | -1.18 | Y | -0.96 | 0.24 | 1.21 | Y |
| POLB | 1.46 | 0.34 | -1.12 | Y | -0.79 | 0.41 | 1.20 | Y |
| PQBP1 | 1.99 | 1.05 | -0.95 | Y | -0.46 | 0.75 | 1.20 | Y |
| POLE | 0.48 | -0.89 | -1.38 | Y | -0.95 | 0.25 | 1.20 | Y |
| NFKBIA | 2.09 | -0.67 | -2.76 | Y | 0.47 | 1.67 | 1.20 | Y |
| HNRNPUL1 | 1.81 | -0.97 | -2.78 | Y | -0.07 | 1.12 | 1.19 | Y |
| MCM7 | 0.04 | -0.85 | -0.89 | Y | 0.49 | 1.68 | 1.19 | Y |
| CREB3L1 | 0.71 | -0.73 | -1.45 | Y | -0.43 | 0.76 | 1.19 | Y |
| ZNF410 | 1.64 | -0.59 | -2.23 | Y | -0.55 | 0.63 | 1.18 | Y |
| OLIG2 | 1.17 | 0.19 | -0.98 | Y | -0.79 | 0.37 | 1.16 | Y |
| AIM2 | 0.69 | -0.34 | -1.03 | Y | -0.43 | 0.73 | 1.16 | Y |
| ATPIF1 | 0.92 | -0.43 | -1.35 | Y | 0.15 | 1.31 | 1.16 | Y |
| RCOR | 1.37 | -0.77 | -2.14 | Y | 0.09 | 1.23 | 1.14 | Y |
| PDCD2 | 0.61 | -0.79 | -1.40 | Y | -0.21 | 0.93 | 1.14 | Y |
| TBX5 | 0.44 | -0.66 | -1.10 | Y | -0.58 | 0.56 | 1.14 | Y |
| BHLHB2 | 0.73 | -0.75 | -1.48 | Y | -0.40 | 0.74 | 1.14 | Y |
| DHX29 | 0.98 | -0.04 | -1.02 | Y | -0.94 | 0.20 | 1.14 | Y |
| ELAVL2 | 1.78 | -0.31 | -2.09 | Y | -0.14 | 0.99 | 1.14 | Y |
| SF1 | 0.66 | -0.37 | -1.04 | Y | 0.81 | 1.94 | 1.13 | Y |
| UBE2V1 | 1.21 | -0.79 | -2.00 | Y | -0.34 | 0.80 | 1.13 | Y |
| APOH | 0.41 | -1.49 | -1.90 | Y | -0.89 | 0.24 | 1.13 | Y |
| BRD7 | 1.84 | -0.78 | -2.63 | Y | -0.81 | 0.32 | 1.12 | Y |
| ERI3 | 2.24 | -0.28 | -2.52 | Y | -0.21 | 0.92 | 1.12 | Y |
| CASQ2 | 2.37 | 1.00 | -1.37 | Y | -0.12 | 0.98 | 1.11 | Y |
| SP140L | 1.76 | 0.83 | -0.93 | Y | -0.96 | 0.15 | 1.11 | Y |
| EIF2C2 | 0.74 | -0.28 | -1.01 | Y | -0.67 | 0.43 | 1.10 | Y |
| HADHA | 1.47 | 0.33 | -1.14 | Y | -0.32 | 0.77 | 1.10 | Y |
| ARNTL | 0.77 | -0.31 | -1.08 | Y | -0.39 | 0.71 | 1.10 | Y |
| CUX1 | 1.37 | 0.45 | -0.92 | Y | 0.13 | 1.22 | 1.09 | Y |
| UBE2K | 1.63 | -0.95 | -2.58 | Y | -0.74 | 0.35 | 1.09 | Y |
| ESRRG | 2.47 | -0.33 | -2.80 | Y | 0.03 | 1.12 | 1.09 | Y |
| GADD45A | 2.17 | -0.15 | -2.33 | Y | -0.41 | 0.67 | 1.07 | Y |
| ZNF764 | 0.87 | -0.29 | -1.16 | Y | -0.45 | 0.62 | 1.07 | Y |
| RAB14 | 0.27 | -0.88 | -1.15 | Y | -0.76 | 0.30 | 1.06 | Y |
| ZKSCAN5 | 1.57 | -0.40 | -1.97 | Y | 0.21 | 1.27 | 1.06 | Y |
| LSM4 | 0.62 | -1.38 | -2.00 | Y | -0.49 | 0.57 | 1.06 | Y |
| HNRPLL | 0.61 | -0.65 | -1.26 | Y | -0.69 | 0.36 | 1.06 | Y |
| HMGN1 | 1.66 | -0.57 | -2.23 | Y | 0.69 | 1.74 | 1.04 | Y |
| ZNF207 | 0.24 | -0.84 | -1.07 | Y | -0.39 | 0.65 | 1.04 | Y |
| NXF3 | 0.40 | -0.67 | -1.07 | Y | -0.42 | 0.62 | 1.04 | Y |
| SNAI3 | 0.81 | -0.58 | -1.39 | Y | -0.60 | 0.44 | 1.04 | Y |
| CRX | 1.52 | 0.52 | -1.00 | Y | -0.45 | 0.58 | 1.03 | Y |
| PSMA1 | 1.41 | -0.20 | -1.61 | Y | 0.10 | 1.12 | 1.03 | Y |
| ARL6IP4 | 1.66 | -0.20 | -1.86 | Y | -0.30 | 0.73 | 1.03 | Y |
| PCNA | 1.33 | 0.32 | -1.01 | Y | -0.83 | 0.20 | 1.03 | Y |
| RNMTL1 | 0.62 | -0.67 | -1.29 | Y | -0.64 | 0.38 | 1.02 | Y |
| VPS24 | 3.47 | -0.52 | -3.99 | Y | -0.75 | 0.28 | 1.02 | Y |
| SSBP1 | 2.20 | -0.65 | -2.85 | Y | -1.03 | -0.01 | 1.02 | Y |
| RBL2 | 1.59 | -0.11 | -1.70 | Y | -1.33 | -0.31 | 1.02 | Y |
| PRC | 0.85 | -0.87 | -1.72 | Y | 0.11 | 1.12 | 1.01 | Y |
| ZNF137 | 0.79 | -0.62 | -1.41 | Y | -0.68 | 0.33 | 1.01 | Y |
| C14orf140 | 0.66 | -0.97 | -1.63 | Y | -0.55 | 0.46 | 1.01 | Y |
| CCNL1 | 1.16 | -0.84 | -2.00 | Y | -0.19 | 0.81 | 1.01 | Y |
| CHD2 | 0.88 | -0.44 | -1.32 | Y | -0.43 | 0.57 | 1.00 | Y |
| UNG | 2.20 | -0.39 | -2.59 | Y | -0.42 | 0.58 | 1.00 | Y |
| GCC1 | 0.12 | -1.53 | -1.66 | Y | -0.14 | 0.87 | 1.00 | Y |
| LACTB2 | 0.99 | -0.12 | -1.10 | Y | -0.35 | 0.65 | 1.00 | Y |
| C13orf15 | 0.85 | -0.04 | -0.90 | Y | -0.18 | 0.81 | 0.99 | Y |
| CC2D1A | 1.13 | -0.57 | -1.70 | Y | -0.52 | 0.47 | 0.98 | Y |
| TACO1 | 1.19 | 0.11 | -1.08 | Y | -0.08 | 0.90 | 0.98 | Y |
| EIF4B | 2.02 | -0.16 | -2.18 | Y | -0.70 | 0.28 | 0.98 | Y |
| KCNIP4 | 0.36 | -0.85 | -1.22 | Y | -0.83 | 0.14 | 0.97 | Y |
| POLR3E | 0.46 | -0.59 | -1.05 | Y | -1.31 | -0.34 | 0.97 | Y |
| VAMP1 | 2.34 | -0.21 | -2.55 | Y | -0.95 | 0.02 | 0.97 | Y |
| PPP1R13L | 1.09 | -0.23 | -1.32 | Y | -1.07 | -0.10 | 0.97 | Y |
| FHL3 | 1.40 | -0.62 | -2.02 | Y | -0.75 | 0.21 | 0.97 | Y |
| MLXIPL | 0.44 | -0.68 | -1.12 | Y | -0.73 | 0.24 | 0.97 | Y |
| SIRT6 | 1.24 | -0.01 | -1.24 | Y | -1.33 | -0.37 | 0.96 | Y |
| SART3 | 4.71 | -0.02 | -4.73 | Y | 2.28 | 3.24 | 0.96 | Y |
| THOC3 | 2.10 | 0.09 | -2.01 | Y | 0.32 | 1.27 | 0.95 | Y |
| GTPBP3 | 0.82 | -0.29 | -1.10 | Y | -0.56 | 0.39 | 0.95 | Y |
| ZNF428 | 4.51 | 1.10 | -3.42 | Y | -0.86 | 0.09 | 0.94 | Y |
| ATP5C1 | 0.79 | -0.18 | -0.96 | Y | -0.05 | 0.89 | 0.94 | Y |
| ZNF85 | 0.73 | -0.16 | -0.89 | Y | -0.80 | 0.14 | 0.94 | Y |
| CBX3 | 1.24 | 0.16 | -1.07 | Y | -0.34 | 0.60 | 0.94 | Y |
| DAZAP1 | 0.92 | -0.87 | -1.79 | Y | 0.09 | 1.03 | 0.93 | Y |
| TMEM38A | 0.74 | -0.65 | -1.39 | Y | -0.67 | 0.26 | 0.93 | Y |
| RHOXF2 | 1.33 | -0.80 | -2.14 | Y | 0.06 | 0.99 | 0.93 | Y |
| GATAD1 | 0.09 | -1.09 | -1.18 | Y | -0.82 | 0.11 | 0.93 | Y |
| SUMO4 | 1.65 | -0.85 | -2.50 | Y | -0.67 | 0.25 | 0.92 | Y |
| CPT2 | 2.42 | 1.04 | -1.38 | Y | -0.03 | 0.89 | 0.92 | Y |
| ZNF471 | 0.63 | -0.76 | -1.39 | Y | -0.85 | 0.07 | 0.91 | Y |
| XAB2 | 1.33 | -0.29 | -1.62 | Y | -0.50 | 0.40 | 0.91 | Y |
| ATP5J | 1.22 | 0.06 | -1.16 | Y | -0.23 | 0.67 | 0.90 | Y |
| SLC4A1AP | 3.29 | 0.05 | -3.24 | Y | 0.31 | 1.22 | 0.90 | Y |
| ZNF394 | 1.09 | 0.07 | -1.03 | Y | -0.25 | 0.65 | 0.90 | Y |
| NHLH2 | 1.02 | -0.36 | -1.38 | Y | 0.57 | 1.46 | 0.89 | Y |
| CNOT3 | 2.05 | 0.97 | -1.08 | Y | 0.46 | 1.35 | 0.89 | Y |
| ZFP36L1 | 3.07 | -0.64 | -3.71 | Y | 0.89 | 1.77 | 0.88 | Y |
| CSTF2 | 1.16 | 0.02 | -1.14 | Y | -0.16 | 0.73 | 0.88 | Y |
| ZNF331 | 1.51 | -0.23 | -1.74 | Y | 0.01 | 0.89 | 0.88 | Y |
| IRF6 | 1.14 | -0.04 | -1.18 | Y | -0.77 | 0.11 | 0.88 | Y |
| SFRS15 | 0.99 | 0.05 | -0.94 | Y | -0.37 | 0.50 | 0.87 | Y |
| ZBTB43 | 1.56 | -0.80 | -2.36 | Y | 0.08 | 0.95 | 0.87 | Y |
| MADH9 | 1.71 | -0.37 | -2.08 | Y | -0.37 | 0.50 | 0.87 | Y |
| NXT1 | 0.78 | -0.35 | -1.12 | Y | -0.47 | 0.40 | 0.87 | Y |
| SMARCA1 | 1.49 | -1.43 | -2.92 | Y | -0.52 | 0.35 | 0.86 | Y |
| EP400 | -0.12 | -1.56 | -1.45 | Y | -0.83 | 0.03 | 0.86 | Y |
| TRIM31 | 1.05 | -0.16 | -1.21 | Y | -0.64 | 0.22 | 0.86 | Y |
| RNF146 | 0.46 | -1.30 | -1.76 | Y | -0.68 | 0.18 | 0.86 | Y |
| UCP2 | 1.72 | -0.94 | -2.66 | Y | 0.58 | 1.43 | 0.86 | Y |
| NCBP1 | 2.02 | -0.59 | -2.60 | Y | -0.22 | 0.63 | 0.85 | Y |
| PDK3 | 3.03 | 0.55 | -2.48 | Y | -0.33 | 0.52 | 0.85 | Y |
| ANXA11 | 1.83 | -0.62 | -2.45 | Y | -0.44 | 0.40 | 0.84 | Y |
| ZNF333 | 1.25 | 0.05 | -1.20 | Y | 0.14 | 0.98 | 0.84 | Y |
| IRX2 | 0.89 | -0.06 | -0.95 | Y | -0.46 | 0.38 | 0.84 | Y |
| TAF12 | 0.85 | -0.12 | -0.97 | Y | 0.66 | 1.49 | 0.84 | Y |
| MVP | 0.13 | -0.76 | -0.89 | Y | 0.07 | 0.91 | 0.84 | Y |
| RBPJ | 1.08 | -0.28 | -1.36 | Y | 0.30 | 1.13 | 0.84 | Y |
| GRLF1 | 0.79 | -0.48 | -1.27 | Y | -0.45 | 0.38 | 0.84 | Y |
| ZNF280A | 0.36 | -0.53 | -0.89 | Y | -0.38 | 0.45 | 0.83 | Y |
| ZNF323 | 0.85 | -0.22 | -1.06 | Y | 0.88 | 1.71 | 0.83 | Y |
| NRIP2 | 1.72 | 0.39 | -1.33 | Y | -0.91 | -0.08 | 0.82 | Y |
| FYN | 1.60 | 0.70 | -0.89 | Y | 0.89 | 1.72 | 0.82 | Y |
| PRIM1 | 0.27 | -0.70 | -0.97 | Y | -0.61 | 0.21 | 0.82 | Y |
| TTF2 | 1.86 | -0.52 | -2.38 | Y | -0.29 | 0.53 | 0.82 | Y |
| MRPS31 | 0.93 | -0.50 | -1.43 | Y | -0.67 | 0.15 | 0.82 | Y |
| HSPB8 | 0.79 | -0.58 | -1.37 | Y | -1.16 | -0.35 | 0.82 | Y |
| BOLA3 | 0.73 | -0.41 | -1.13 | Y | 0.19 | 1.01 | 0.82 | Y |
| NDUFV1 | 0.75 | -1.18 | -1.93 | Y | -1.11 | -0.30 | 0.81 | Y |
| CAMK2D | 2.00 | -0.06 | -2.07 | Y | 0.15 | 0.96 | 0.81 | Y |
| NDUFA4 | 0.49 | -0.44 | -0.93 | Y | -0.92 | -0.11 | 0.81 | Y |
| RAN | 0.89 | -0.23 | -1.13 | Y | 0.99 | 1.78 | 0.79 | Y |
| C13orf10 | 2.59 | -0.76 | -3.35 | Y | -0.45 | 0.34 | 0.79 | Y |
| VDAC3 | 1.09 | -0.49 | -1.58 | Y | -0.66 | 0.13 | 0.79 | Y |
| DFFB | 0.85 | -0.38 | -1.23 | Y | -0.78 | 0.00 | 0.79 | Y |
| SLC13A1 | 0.27 | -0.83 | -1.10 | Y | -0.98 | -0.20 | 0.79 | Y |
| E2F7 | 1.08 | -0.50 | -1.58 | Y | 0.99 | 1.77 | 0.78 | Y |
| ZCCHC12 | 1.95 | 0.07 | -1.88 | Y | -0.48 | 0.30 | 0.77 | Y |
| SMAD4 | 1.06 | -0.60 | -1.67 | Y | -0.03 | 0.74 | 0.77 | Y |
| HIC2 | 1.85 | 0.42 | -1.43 | Y | -0.55 | 0.22 | 0.76 | Y |
| NPM1 | 1.18 | -0.09 | -1.28 | Y | -0.62 | 0.15 | 0.76 | Y |
| DHODH | 1.38 | -0.37 | -1.75 | Y | -0.38 | 0.38 | 0.76 | Y |
| PRR15L | 1.76 | -0.23 | -1.99 | Y | -1.26 | -0.50 | 0.75 | Y |
| DTX2 | 1.35 | 0.39 | -0.95 | Y | -0.67 | 0.09 | 0.75 | Y |
| SF3B3 | 1.06 | -0.19 | -1.25 | Y | -0.36 | 0.39 | 0.75 | Y |
| GAS7 | 1.38 | -0.21 | -1.59 | Y | 0.10 | 0.84 | 0.74 | Y |
| SET | 0.30 | -0.92 | -1.22 | Y | -0.72 | 0.01 | 0.73 | Y |
| PPIB | 0.94 | -0.50 | -1.44 | Y | 0.13 | 0.86 | 0.73 | Y |
| RBM19 | 1.32 | -0.50 | -1.82 | Y | -0.13 | 0.60 | 0.73 | Y |
| ZNF597 | 0.97 | -0.34 | -1.31 | Y | -0.63 | 0.09 | 0.73 | Y |
| ASPH | 0.46 | -0.70 | -1.16 | Y | -1.08 | -0.36 | 0.72 | Y |
| BCR | 2.31 | -0.21 | -2.52 | Y | 0.02 | 0.72 | 0.71 | Y |
| ZNF226 | 1.65 | 0.68 | -0.97 | Y | 0.22 | 0.93 | 0.71 | Y |
| RAB10 | 1.54 | 0.11 | -1.43 | Y | -0.39 | 0.32 | 0.71 | Y |
| SECISBP2 | 2.04 | -0.77 | -2.80 | Y | -1.02 | -0.31 | 0.70 | Y |
| RPS1KL1 | 1.42 | 0.03 | -1.39 | Y | -0.48 | 0.23 | 0.70 | Y |
| HCLS1 | 0.30 | -0.65 | -0.95 | Y | -0.30 | 0.40 | 0.70 | Y |
| CASS4 | 1.40 | -0.23 | -1.63 | Y | -0.59 | 0.10 | 0.70 | Y |
| MBTPS2 | 0.70 | -0.82 | -1.52 | Y | -0.53 | 0.16 | 0.70 | Y |
| TYK2 | 0.73 | -0.69 | -1.42 | Y | -0.62 | 0.08 | 0.70 | Y |
| NDUFS4 | 2.33 | 0.04 | -2.28 | Y | -0.32 | 0.38 | 0.70 | Y |
| TRIM28 | 0.38 | -0.65 | -1.03 | Y | 0.67 | 1.35 | 0.69 | Y |
| ARAF | 1.58 | -0.34 | -1.92 | Y | -0.95 | -0.26 | 0.69 | Y |
| EIF3F | 1.26 | 0.16 | -1.10 | Y | -0.49 | 0.19 | 0.68 | Y |
| ELAVL3 | 1.60 | -0.41 | -2.02 | Y | -0.90 | -0.22 | 0.68 | Y |
| RUNX1T1 | 1.44 | 0.38 | -1.06 | Y | -0.22 | 0.45 | 0.68 | Y |
| PRDM10 | 0.80 | -0.30 | -1.10 | Y | -1.42 | -0.75 | 0.67 | Y |
| VAMP3 | 0.97 | -0.58 | -1.55 | Y | -0.76 | -0.09 | 0.67 | Y |
| TFE3 | 1.42 | 0.41 | -1.00 | Y | -0.71 | -0.05 | 0.67 | Y |
| SFRS5 | 0.57 | -1.06 | -1.64 | Y | -0.28 | 0.39 | 0.67 | Y |
| APITD1 | 0.98 | -0.07 | -1.05 | Y | -0.13 | 0.53 | 0.66 | Y |
| IKZF1 | 2.03 | -0.30 | -2.33 | Y | -0.32 | 0.33 | 0.66 | Y |
| HEXIM1 | 2.40 | -0.14 | -2.54 | Y | 0.39 | 1.04 | 0.65 | Y |
| SNRNP70 | 2.11 | 0.18 | -1.93 | Y | -0.18 | 0.47 | 0.65 | Y |
| AKAP8 | 1.71 | -0.80 | -2.51 | Y | -0.18 | 0.46 | 0.64 | Y |
| EIF4H | 2.26 | -1.23 | -3.49 | Y | 0.09 | 0.73 | 0.64 | Y |
| ING3 | 1.57 | -0.02 | -1.59 | Y | -0.11 | 0.53 | 0.63 | Y |
| MEF2D | 1.75 | 0.38 | -1.37 | Y | 0.15 | 0.77 | 0.62 | Y |
| GFER | 1.55 | 0.03 | -1.52 | Y | -0.74 | -0.13 | 0.62 | Y |
| SOX9 | 1.56 | 0.18 | -1.38 | Y | -0.06 | 0.55 | 0.62 | Y |
| NCOA3 | 2.14 | 0.64 | -1.49 | Y | 0.02 | 0.63 | 0.61 | Y |
| GDI2 | 2.13 | 0.61 | -1.52 | Y | 0.34 | 0.95 | 0.61 | Y |
| FBXO3 | 1.22 | -0.47 | -1.70 | Y | -0.40 | 0.20 | 0.61 | Y |
| RIPK3 | 0.41 | -0.53 | -0.94 | Y | -0.10 | 0.50 | 0.60 | Y |
| ERMP1 | 0.61 | -0.82 | -1.43 | Y | -0.19 | 0.40 | 0.59 | Y |
| HOXD8 | 1.88 | -0.18 | -2.05 | Y | -0.06 | 0.53 | 0.59 | Y |
| ATXN3 | 0.68 | -0.51 | -1.19 | Y | -0.68 | -0.09 | 0.59 | Y |
| HAX1 | 1.14 | -0.62 | -1.76 | Y | -0.11 | 0.47 | 0.59 | Y |
| ETV7 | 2.00 | -0.87 | -2.87 | Y | 0.96 | 1.55 | 0.59 | Y |
| YWHAE | 1.21 | -0.31 | -1.52 | Y | -0.04 | 0.54 | 0.58 | Y |
| HDGFRP2 | 1.59 | 0.70 | -0.89 | Y | 0.12 | 0.70 | 0.58 | Y |
| ANXA2 | 2.63 | 1.60 | -1.03 | Y | -0.25 | 0.33 | 0.58 | Y |
| EVX1 | 1.52 | -0.39 | -1.92 | Y | -0.13 | 0.45 | 0.57 | Y |
| MAOB | 0.44 | -0.61 | -1.05 | Y | 1.00 | 1.57 | 0.57 | Y |
| CRK | 0.66 | -0.36 | -1.02 | Y | -0.22 | 0.35 | 0.57 | Y |
| LMX1B | 3.16 | 2.18 | -0.99 | Y | -0.04 | 0.53 | 0.57 | Y |
| NXPH3 | 1.05 | -0.78 | -1.83 | Y | -0.35 | 0.22 | 0.57 | Y |
| OAS1 | 0.98 | -0.18 | -1.16 | Y | -0.25 | 0.32 | 0.57 | Y |
| R3HDM2 | 1.98 | 0.67 | -1.31 | Y | 0.75 | 1.32 | 0.57 | Y |
| BLOC1S1 | 1.06 | -0.34 | -1.40 | Y | -0.77 | -0.20 | 0.56 | Y |
| YWHAH | 2.82 | -0.09 | -2.91 | Y | 0.73 | 1.30 | 0.56 | Y |
| CSNK2A1 | 0.67 | -1.37 | -2.04 | Y | 0.06 | 0.63 | 0.56 | Y |
| NDUFS1 | 0.74 | -1.13 | -1.87 | Y | -0.32 | 0.25 | 0.56 | Y |
| TERF2 | 0.93 | -0.95 | -1.88 | Y | 0.01 | 0.57 | 0.56 | Y |
| LDB1 | 2.05 | -0.31 | -2.36 | Y | -0.65 | -0.09 | 0.56 | Y |
| TGFB1I1 | 1.21 | 0.33 | -0.88 | Y | -0.07 | 0.49 | 0.56 | Y |
| KLF4 | 1.87 | 0.25 | -1.62 | Y | 0.60 | 1.16 | 0.56 | Y |
| SFRS11 | 3.13 | 0.99 | -2.13 | Y | -0.47 | 0.08 | 0.56 | Y |
| MRPS28 | 0.81 | -0.54 | -1.36 | Y | -0.50 | 0.05 | 0.56 | Y |
| MAGED1 | 1.49 | -0.80 | -2.29 | Y | -0.67 | -0.11 | 0.55 | Y |
| BCS1L | 0.87 | -0.98 | -1.86 | Y | -0.37 | 0.18 | 0.55 | Y |
| PRDX5 | 0.42 | -0.57 | -0.99 | Y | -0.33 | 0.21 | 0.55 | Y |
| LIG1 | 1.79 | -0.04 | -1.83 | Y | -0.71 | -0.16 | 0.55 | Y |
| PIM1 | 0.87 | -0.09 | -0.97 | Y | 0.05 | 0.60 | 0.55 | Y |
| DAPK2 | 3.20 | 0.41 | -2.79 | Y | -0.30 | 0.24 | 0.54 | Y |
| PIM3 | 1.65 | 0.13 | -1.53 | Y | 0.30 | 0.83 | 0.54 | Y |
| ZNF280D | 1.65 | -0.86 | -2.51 | Y | 0.64 | 1.17 | 0.53 | Y |
| MRPS14 | 0.68 | -0.21 | -0.89 | Y | 0.74 | 1.27 | 0.53 | Y |
| MBD1 | 1.47 | 0.53 | -0.94 | Y | -0.20 | 0.33 | 0.53 | Y |
| EIF3I | 1.67 | 0.37 | -1.30 | Y | -0.98 | -0.45 | 0.53 | Y |
| ORC1L | 0.33 | -0.69 | -1.01 | Y | -0.99 | -0.47 | 0.52 | Y |
| CPSF4 | 1.49 | 0.20 | -1.29 | Y | 0.02 | 0.54 | 0.52 | Y |
| SDHAF2 | 0.01 | -1.02 | -1.03 | Y | 0.50 | 1.02 | 0.52 | Y |
| MCCC2 | 0.52 | -0.61 | -1.12 | Y | 0.18 | 0.70 | 0.52 | Y |
| LOC649946 | 1.82 | -0.78 | -2.59 | Y | -1.46 | -0.95 | 0.51 | Y |
| ACADS | 0.41 | -1.41 | -1.82 | Y | 0.47 | 0.99 | 0.51 | Y |
| IRF3 | 1.59 | 0.02 | -1.57 | Y | 0.16 | 0.66 | 0.50 | Y |
| GRHL1 | 1.13 | -0.52 | -1.65 | Y | 0.23 | 0.73 | 0.50 | Y |
| VDAC2 | 0.60 | -0.89 | -1.49 | Y | 0.81 | 1.31 | 0.50 | Y |
| NARS | 1.34 | -0.51 | -1.86 | Y | -0.23 | 0.27 | 0.50 | Y |
| ZMAT4 | 1.62 | -0.11 | -1.73 | Y | 0.42 | 0.92 | 0.50 | Y |
| TNIK | 1.84 | -0.15 | -1.98 | Y | -0.33 | 0.17 | 0.50 | Y |
| RAD51 | 1.31 | 0.33 | -0.98 | Y | -0.45 | 0.04 | 0.49 | Y |
| SPATS2 | 0.63 | -1.07 | -1.70 | Y | -0.31 | 0.18 | 0.49 | Y |
| EBF1 | 1.56 | 0.18 | -1.38 | Y | -0.05 | 0.43 | 0.47 | Y |
| CRIPT | 0.12 | -0.79 | -0.91 | Y | -0.14 | 0.34 | 0.47 | Y |
| PAXIP1 | 1.56 | -0.01 | -1.57 | Y | -0.78 | -0.31 | 0.47 | Y |
| NCALD | 0.83 | -0.35 | -1.18 | Y | -0.44 | 0.02 | 0.47 | Y |
| RXRG | 0.73 | -0.39 | -1.12 | Y | 0.02 | 0.49 | 0.47 | Y |
| SF3A3 | 0.31 | -0.69 | -1.00 | Y | -0.01 | 0.45 | 0.47 | Y |
| MRPL1 | 2.04 | 0.16 | -1.89 | Y | -0.50 | -0.04 | 0.46 | Y |
| STARD3 | 0.81 | -0.22 | -1.03 | Y | -0.04 | 0.42 | 0.46 | Y |
| MECP2 | 2.88 | 1.72 | -1.16 | Y | 0.98 | 1.44 | 0.46 | Y |
| C19orf43 | 0.21 | -0.70 | -0.91 | Y | -0.16 | 0.30 | 0.45 | Y |
| RNF181 | 1.76 | -0.31 | -2.07 | Y | -0.58 | -0.13 | 0.45 | Y |
| PTK6 | 2.28 | 1.17 | -1.11 | Y | 0.01 | 0.46 | 0.45 | Y |
| DECR1 | 1.34 | -0.22 | -1.56 | Y | -0.53 | -0.08 | 0.45 | Y |
| NFU1 | 0.79 | -0.75 | -1.54 | Y | -0.45 | -0.01 | 0.44 | Y |
| LDB3 | 0.90 | -0.15 | -1.05 | Y | -0.96 | -0.53 | 0.43 | - |
| KAT5 | 1.70 | -0.72 | -2.42 | Y | -0.31 | 0.11 | 0.43 | - |
| SPRYD4 | 1.80 | 0.05 | -1.75 | Y | -0.04 | 0.39 | 0.43 | - |
| RBM9 | 0.95 | -0.29 | -1.24 | Y | -1.36 | -0.93 | 0.43 | - |
| NDUFA3 | 1.06 | -0.77 | -1.83 | Y | 0.15 | 0.57 | 0.42 | - |
| THRA | 1.44 | 0.42 | -1.03 | Y | -0.40 | 0.02 | 0.42 | - |
| TAF5L | -0.25 | -1.24 | -0.99 | Y | -0.30 | 0.11 | 0.41 | - |
| NEK10 | 1.56 | 0.05 | -1.51 | Y | 0.54 | 0.95 | 0.41 | - |
| TAF9 | 1.21 | -0.29 | -1.50 | Y | -0.84 | -0.43 | 0.41 | - |
| N6AMT1 | -0.06 | -1.04 | -0.98 | Y | 0.00 | 0.41 | 0.41 | - |
| ERBB3 | 0.34 | -0.86 | -1.20 | Y | 1.18 | 1.59 | 0.41 | - |
| METTL3 | 1.95 | 0.19 | -1.76 | Y | 0.05 | 0.45 | 0.40 | - |
| KIAA0368 | 1.37 | -0.71 | -2.08 | Y | -0.85 | -0.45 | 0.40 | - |
| SLC27A2 | 0.57 | -0.65 | -1.23 | Y | -0.50 | -0.10 | 0.40 | - |
| RBMS3 | 0.95 | -1.27 | -2.23 | Y | -0.41 | -0.01 | 0.40 | - |
| NACC2 | 2.32 | -0.14 | -2.46 | Y | -0.34 | 0.06 | 0.40 | - |
| THOC1 | 1.27 | 0.10 | -1.17 | Y | -0.37 | 0.02 | 0.40 | - |
| MTERFD2 | 1.09 | 0.17 | -0.92 | Y | -0.48 | -0.09 | 0.40 | - |
| SIRT1 | 2.34 | -0.30 | -2.65 | Y | 1.21 | 1.60 | 0.39 | - |
| HEMK1 | 1.05 | -0.38 | -1.43 | Y | -0.37 | 0.02 | 0.39 | - |
| SUV39H1 | 1.13 | -0.73 | -1.86 | Y | -0.15 | 0.24 | 0.39 | - |
| TAF1 | 0.88 | -1.01 | -1.90 | Y | -0.64 | -0.25 | 0.39 | - |
| ATG3 | 0.27 | -1.08 | -1.35 | Y | -0.37 | 0.02 | 0.39 | - |
| ING1 | 1.23 | -0.46 | -1.69 | Y | 1.06 | 1.45 | 0.39 | - |
| BNIP3L | 2.61 | 1.68 | -0.93 | Y | 0.59 | 0.98 | 0.39 | - |
| CELF6 | 1.75 | 0.35 | -1.41 | Y | -0.81 | -0.43 | 0.39 | - |
| RNF115 | 1.66 | 0.00 | -1.66 | Y | 0.50 | 0.88 | 0.38 | - |
| ZNRD1 | 0.96 | -0.20 | -1.16 | Y | -0.17 | 0.21 | 0.38 | - |
| SGK2 | 0.89 | -0.76 | -1.66 | Y | 0.17 | 0.55 | 0.38 | - |
| PFDN5 | 1.40 | 0.26 | -1.14 | Y | -0.28 | 0.10 | 0.38 | - |
| NRF1 | 1.68 | 0.06 | -1.62 | Y | -0.30 | 0.08 | 0.37 | - |
| ZNF10 | 1.55 | 0.25 | -1.30 | Y | -0.29 | 0.08 | 0.37 | - |
| SEC14L2 | 0.45 | -0.75 | -1.20 | Y | -0.27 | 0.10 | 0.37 | - |
| CSTF2T | 1.60 | 0.10 | -1.50 | Y | -0.85 | -0.48 | 0.37 | - |
| STUB1 | 0.84 | -0.19 | -1.03 | Y | -0.17 | 0.20 | 0.37 | - |
| UTP6 | 1.09 | -0.47 | -1.56 | Y | -0.27 | 0.09 | 0.37 | - |
| FXC1 | 1.24 | -0.98 | -2.22 | Y | -0.69 | -0.32 | 0.36 | - |
| ITGB1 | 1.96 | -1.14 | -3.09 | Y | 0.19 | 0.55 | 0.36 | - |
| FOSL1 | 0.97 | -0.23 | -1.20 | Y | -0.69 | -0.32 | 0.36 | - |
| DRAM1 | 1.15 | 0.01 | -1.13 | Y | -0.51 | -0.15 | 0.36 | - |
| FXYD1 | -0.09 | -1.13 | -1.04 | Y | -0.06 | 0.29 | 0.35 | - |
| ZNF302 | 1.43 | -0.29 | -1.72 | Y | -0.53 | -0.18 | 0.35 | - |
| RBM46 | 0.72 | -0.42 | -1.14 | Y | 0.85 | 1.19 | 0.34 | - |
| RABL3 | 1.12 | -0.52 | -1.64 | Y | -0.92 | -0.58 | 0.34 | - |
| MECR | 0.82 | -0.59 | -1.41 | Y | -0.48 | -0.14 | 0.34 | - |
| LHX6 | 0.82 | -0.12 | -0.93 | Y | -0.37 | -0.04 | 0.33 | - |
| ZBED2 | 1.37 | -0.66 | -2.03 | Y | 0.56 | 0.89 | 0.33 | - |
| CDK19 | 0.43 | -0.53 | -0.96 | Y | 0.18 | 0.51 | 0.33 | - |
| PRMT1 | 1.07 | -0.55 | -1.61 | Y | -0.92 | -0.59 | 0.33 | - |
| BRD4 | 1.35 | -0.28 | -1.63 | Y | 0.14 | 0.46 | 0.32 | - |
| NUDC | 1.36 | -0.15 | -1.50 | Y | -0.62 | -0.30 | 0.32 | - |
| PAGE4 | 0.52 | -0.40 | -0.93 | Y | -0.53 | -0.21 | 0.32 | - |
| MAST4 | 0.62 | -0.29 | -0.92 | Y | 0.14 | 0.45 | 0.31 | - |
| TOMM34 | 2.02 | 0.37 | -1.65 | Y | 0.04 | 0.34 | 0.30 | - |
| CANX | 0.55 | -0.40 | -0.95 | Y | 0.51 | 0.81 | 0.30 | - |
| AIG1 | 0.69 | -0.50 | -1.19 | Y | -0.43 | -0.13 | 0.30 | - |
| RAD51C | 1.99 | -0.34 | -2.33 | Y | -1.03 | -0.74 | 0.30 | - |
| MSN | 1.23 | -0.47 | -1.70 | Y | -0.29 | 0.00 | 0.29 | - |
| PPP4C | 0.39 | -0.54 | -0.93 | Y | -0.37 | -0.08 | 0.29 | - |
| FHL1 | 2.32 | 0.24 | -2.08 | Y | -0.03 | 0.25 | 0.28 | - |
| LUC7L3 | 0.97 | 0.06 | -0.92 | Y | 0.06 | 0.34 | 0.28 | - |
| C9orf156 | 2.17 | 0.48 | -1.69 | Y | 0.42 | 0.70 | 0.28 | - |
| DGUOK | 1.00 | -0.99 | -1.99 | Y | 0.10 | 0.37 | 0.27 | - |
| EIF2B1 | 0.38 | -0.62 | -1.00 | Y | -0.35 | -0.08 | 0.27 | - |
| E2F6 | 2.29 | -0.62 | -2.91 | Y | 0.40 | 0.67 | 0.27 | - |
| MDH2 | 1.23 | -0.71 | -1.94 | Y | -0.08 | 0.18 | 0.26 | - |
| CYCS | 0.35 | -0.56 | -0.90 | Y | -0.66 | -0.40 | 0.26 | - |
| BCL2L11 | 1.86 | -0.99 | -2.85 | Y | 0.26 | 0.52 | 0.26 | - |
| FEZF2 | 1.16 | -0.41 | -1.57 | Y | -0.35 | -0.09 | 0.26 | - |
| C21orf7 | 0.72 | -0.91 | -1.63 | Y | -0.31 | -0.06 | 0.25 | - |
| BOLL | 1.09 | -0.29 | -1.38 | Y | 0.49 | 0.73 | 0.24 | - |
| SERBP1 | 2.20 | -1.00 | -3.20 | Y | 0.49 | 0.74 | 0.24 | - |
| HADH | 0.82 | -0.17 | -0.99 | Y | -0.04 | 0.20 | 0.24 | - |
| TK1 | 1.21 | 0.13 | -1.07 | Y | -0.20 | 0.05 | 0.24 | - |
| ZNF215 | 0.04 | -0.95 | -0.99 | Y | 0.34 | 0.58 | 0.24 | - |
| FECH | 0.10 | -1.26 | -1.36 | Y | -0.70 | -0.46 | 0.24 | - |
| PRKAA1 | 0.83 | -0.45 | -1.27 | Y | -0.44 | -0.20 | 0.24 | - |
| CDK9 | 1.25 | -0.78 | -2.03 | Y | 0.20 | 0.44 | 0.24 | - |
| TSC22D3 | 0.52 | -0.49 | -1.01 | Y | -0.21 | 0.02 | 0.23 | - |
| ZNF461 | 0.53 | -0.38 | -0.91 | Y | -0.62 | -0.39 | 0.23 | - |
| RAD51L1 | 0.98 | -0.62 | -1.60 | Y | -0.64 | -0.41 | 0.23 | - |
| MEF2C | 1.07 | -0.07 | -1.14 | Y | -0.71 | -0.48 | 0.23 | - |
| PPP1R8 | 1.70 | -0.83 | -2.53 | Y | -0.49 | -0.27 | 0.22 | - |
| RAD23A | 0.70 | -0.71 | -1.41 | Y | -0.22 | 0.00 | 0.22 | - |
| ZNF560 | 1.08 | -0.64 | -1.72 | Y | -0.81 | -0.59 | 0.22 | - |
| TAF1B | 1.16 | 0.25 | -0.91 | Y | -0.15 | 0.06 | 0.21 | - |
| TRIM21 | 1.58 | -0.24 | -1.81 | Y | -0.01 | 0.20 | 0.21 | - |
| CS | 0.20 | -0.72 | -0.93 | Y | 0.24 | 0.45 | 0.21 | - |
| UHMK1 | 0.67 | -0.41 | -1.08 | Y | 0.02 | 0.22 | 0.21 | - |
| FHL2 | 1.28 | -0.47 | -1.75 | Y | -0.65 | -0.45 | 0.20 | - |
| ZNF830 | 3.84 | 0.24 | -3.59 | Y | -0.12 | 0.08 | 0.20 | - |
| FAM117A | 0.96 | -0.58 | -1.54 | Y | -0.16 | 0.03 | 0.19 | - |
| THAP4 | 0.97 | -0.46 | -1.43 | Y | -0.16 | 0.03 | 0.19 | - |
| LRFN3 | 1.18 | -0.56 | -1.74 | Y | 0.03 | 0.22 | 0.19 | - |
| CTCF | 1.41 | -0.75 | -2.16 | Y | -0.26 | -0.08 | 0.18 | - |
| EZR | 1.11 | 0.03 | -1.08 | Y | 1.99 | 2.17 | 0.18 | - |
| PDCD5 | 1.69 | -0.26 | -1.95 | Y | 1.09 | 1.27 | 0.18 | - |
| MRE11A | 4.25 | 2.02 | -2.23 | Y | -0.39 | -0.22 | 0.18 | - |
| EPHB1 | 0.71 | -0.50 | -1.20 | Y | 0.52 | 0.70 | 0.18 | - |
| PRR14 | 0.57 | -0.40 | -0.97 | Y | -0.37 | -0.19 | 0.17 | - |
| CPSF3 | 0.52 | -0.75 | -1.26 | Y | 0.24 | 0.41 | 0.17 | - |
| PECI | 1.98 | 0.23 | -1.75 | Y | -0.24 | -0.07 | 0.17 | - |
| WWP1 | 1.57 | -0.13 | -1.70 | Y | -0.62 | -0.45 | 0.17 | - |
| RBM12 | 1.54 | 0.03 | -1.51 | Y | 0.59 | 0.75 | 0.17 | - |
| DUSP21 | 3.01 | 0.82 | -2.19 | Y | 0.76 | 0.92 | 0.16 | - |
| PSMA7 | 1.53 | 0.14 | -1.39 | Y | -0.53 | -0.39 | 0.15 | - |
| HUMPPA | 2.00 | 1.10 | -0.91 | Y | 1.80 | 1.95 | 0.14 | - |
| COQ6 | 0.31 | -0.93 | -1.24 | Y | -0.24 | -0.10 | 0.14 | - |
| PLG | 0.53 | -0.77 | -1.30 | Y | 0.18 | 0.31 | 0.13 | - |
| BAGE3 | 0.70 | -0.37 | -1.07 | Y | -0.72 | -0.59 | 0.13 | - |
| STK31 | 0.70 | -0.78 | -1.48 | Y | -0.11 | 0.02 | 0.13 | - |
| PHF23 | 3.10 | 2.20 | -0.90 | Y | -0.51 | -0.38 | 0.13 | - |
| FHL5 | 1.91 | -0.03 | -1.94 | Y | -0.11 | 0.02 | 0.13 | - |
| EIF1AD | 3.48 | 0.75 | -2.74 | Y | 1.28 | 1.40 | 0.12 | - |
| CKMT1B | 0.64 | -0.56 | -1.20 | Y | 0.26 | 0.38 | 0.12 | - |
| RCOR3 | 1.56 | -0.02 | -1.59 | Y | -0.27 | -0.16 | 0.11 | - |
| RBPMS | 1.02 | -1.25 | -2.27 | Y | -0.67 | -0.55 | 0.11 | - |
| ARMC6 | 1.68 | -0.83 | -2.51 | Y | -0.10 | 0.00 | 0.11 | - |
| HMGA1 | 1.55 | -0.42 | -1.98 | Y | 0.51 | 0.62 | 0.11 | - |
| TBRG1 | 1.25 | -0.56 | -1.81 | Y | -0.53 | -0.43 | 0.11 | - |
| DHX36 | 0.47 | -0.48 | -0.94 | Y | 0.11 | 0.21 | 0.10 | - |
| SHOX2 | 0.64 | -0.36 | -1.00 | Y | -0.08 | 0.02 | 0.10 | - |
| DNMT3A | 1.41 | -0.33 | -1.73 | Y | -0.42 | -0.33 | 0.09 | - |
| DNM2 | 0.99 | -0.02 | -1.02 | Y | -0.17 | -0.08 | 0.09 | - |
| FGFR4 | 1.31 | -0.32 | -1.63 | Y | 0.84 | 0.93 | 0.09 | - |
| HK1 | 0.38 | -0.60 | -0.98 | Y | -0.18 | -0.10 | 0.08 | - |
| MESP1 | 1.17 | -0.82 | -1.99 | Y | -0.95 | -0.87 | 0.08 | - |
| PKNOX1 | 1.50 | -1.17 | -2.68 | Y | -0.11 | -0.03 | 0.08 | - |
| LUC7L2 | 1.76 | -0.01 | -1.77 | Y | 0.00 | 0.08 | 0.08 | - |
| TSFM | 1.28 | -0.56 | -1.85 | Y | 0.21 | 0.29 | 0.08 | - |
| ANKS1A | 1.71 | 0.73 | -0.98 | Y | -0.38 | -0.30 | 0.08 | - |
| SLC1A3 | 0.55 | -0.88 | -1.43 | Y | -0.22 | -0.15 | 0.07 | - |
| RAB15 | 0.50 | -0.45 | -0.95 | Y | -1.34 | -1.26 | 0.07 | - |
| TAF7 | 3.10 | 0.81 | -2.29 | Y | 0.15 | 0.22 | 0.07 | - |
| CELF4 | 0.30 | -0.58 | -0.88 | Y | 0.08 | 0.15 | 0.07 | - |
| UGP2 | 1.23 | -0.50 | -1.73 | Y | -0.64 | -0.58 | 0.07 | - |
| PRDM14 | 1.78 | 0.18 | -1.60 | Y | 0.35 | 0.41 | 0.06 | - |
| BCL6 | 4.67 | 1.16 | -3.51 | Y | -0.14 | -0.07 | 0.06 | - |
| RNASEH2C | 0.40 | -0.51 | -0.90 | Y | -0.10 | -0.05 | 0.06 | - |
| SIX1 | 1.31 | -0.94 | -2.24 | Y | -0.06 | -0.01 | 0.05 | - |
| RNF135 | 0.94 | -0.15 | -1.09 | Y | 0.14 | 0.19 | 0.05 | - |
| TOMM20 | 0.43 | -0.70 | -1.13 | Y | -0.27 | -0.22 | 0.05 | - |
| SLC25A13 | 0.97 | 0.01 | -0.95 | Y | -0.70 | -0.66 | 0.05 | - |
| VPS72 | 1.82 | 0.74 | -1.08 | Y | 0.02 | 0.07 | 0.05 | - |
| XRCC4 | 2.55 | -0.43 | -2.98 | Y | 0.81 | 0.85 | 0.04 | - |
| DDX6 | 1.29 | -0.50 | -1.78 | Y | 2.01 | 2.06 | 0.04 | - |
| ABCF1 | 1.63 | 0.08 | -1.55 | Y | 0.16 | 0.20 | 0.04 | - |
| STK10 | 0.64 | -0.35 | -0.99 | Y | -0.28 | -0.24 | 0.04 | - |
| POLL | 0.89 | -0.63 | -1.52 | Y | 0.26 | 0.29 | 0.03 | - |
| MEIS1 | 1.10 | -0.49 | -1.58 | Y | 0.93 | 0.96 | 0.03 | - |
| RGS14 | 1.30 | -0.33 | -1.63 | Y | 0.28 | 0.30 | 0.03 | - |
| ETF1 | 0.31 | -1.31 | -1.62 | Y | 0.13 | 0.16 | 0.02 | - |
| WARS2 | 0.74 | -0.76 | -1.50 | Y | -0.40 | -0.38 | 0.02 | - |
| HMGN5 | 1.82 | 0.42 | -1.40 | Y | -0.21 | -0.19 | 0.02 | - |
| RNF8 | 0.86 | -0.96 | -1.82 | Y | -0.01 | 0.00 | 0.01 | - |
| GRPEL2 | 1.30 | -0.09 | -1.38 | Y | -0.65 | -0.64 | 0.01 | - |
| ZNF192 | 1.93 | -0.21 | -2.14 | Y | -0.02 | -0.02 | 0.00 | - |
| NOL3 | 0.91 | -0.87 | -1.79 | Y | 0.06 | 0.06 | 0.00 | - |
| ROD1 | 1.94 | 0.84 | -1.09 | Y | 0.52 | 0.51 | 0.00 | - |
| NUP107 | 1.22 | -0.52 | -1.73 | Y | -0.40 | -0.41 | 0.00 | - |
| FUS | 0.72 | -0.63 | -1.35 | Y | 0.70 | 0.69 | -0.01 | - |
| CNOT8 | 2.47 | -1.10 | -3.57 | Y | -0.42 | -0.43 | -0.01 | - |
| TADA2A | 2.26 | -0.48 | -2.74 | Y | 0.89 | 0.87 | -0.01 | - |
| LIMK2 | 3.34 | 1.82 | -1.52 | Y | 0.40 | 0.38 | -0.02 | - |
| NCBP2 | 1.62 | -0.60 | -2.22 | Y | -0.75 | -0.76 | -0.02 | - |
| E2F4 | 0.78 | -0.14 | -0.92 | Y | -0.44 | -0.47 | -0.02 | - |
| C19orf6 | 1.27 | 0.35 | -0.92 | Y | 0.85 | 0.81 | -0.03 | - |
| ZBTB25 | 1.12 | -0.03 | -1.15 | Y | 0.24 | 0.21 | -0.03 | - |
| RAB35 | 1.87 | -0.47 | -2.34 | Y | -0.29 | -0.34 | -0.04 | - |
| ASPSCR1 | 1.83 | -0.61 | -2.44 | Y | 0.16 | 0.11 | -0.05 | - |
| SP100 | 3.31 | 0.95 | -2.36 | Y | 1.38 | 1.33 | -0.05 | - |
| ETV4 | 2.06 | 0.79 | -1.27 | Y | -0.16 | -0.21 | -0.05 | - |
| BRF2 | 1.50 | -0.56 | -2.06 | Y | 0.20 | 0.15 | -0.05 | - |
| SPG7 | 2.02 | -0.63 | -2.65 | Y | 1.07 | 1.01 | -0.06 | - |
| ARFGAP2 | 1.70 | -0.39 | -2.10 | Y | 0.37 | 0.32 | -0.06 | - |
| BTG4 | 1.58 | -0.49 | -2.07 | Y | -0.49 | -0.55 | -0.06 | - |
| COX6C | 0.93 | -0.28 | -1.21 | Y | -0.40 | -0.46 | -0.06 | - |
| CBX8 | 1.46 | 0.12 | -1.34 | Y | -0.40 | -0.46 | -0.06 | - |
| NEIL2 | 1.06 | -0.42 | -1.48 | Y | 0.33 | 0.27 | -0.06 | - |
| ZDHHC23 | 0.54 | -0.48 | -1.02 | Y | 0.21 | 0.15 | -0.06 | - |
| SLC25A11 | 0.78 | -0.24 | -1.02 | Y | 0.05 | -0.02 | -0.07 | - |
| HELLS | 1.08 | -0.68 | -1.76 | Y | -0.55 | -0.63 | -0.07 | - |
| RFPL3 | 1.14 | -0.75 | -1.89 | Y | -0.28 | -0.36 | -0.08 | - |
| AES | 1.16 | -0.02 | -1.18 | Y | -0.22 | -0.30 | -0.08 | - |
| PEG10 | 1.56 | 0.16 | -1.41 | Y | -0.53 | -0.61 | -0.08 | - |
| DCAF6 | 2.43 | 0.09 | -2.35 | Y | 0.45 | 0.37 | -0.08 | - |
| TRIM16 | 0.35 | -0.68 | -1.02 | Y | -0.31 | -0.40 | -0.09 | - |
| PYGO2 | 0.79 | -0.69 | -1.48 | Y | 0.00 | -0.09 | -0.09 | - |
| NXT2 | 0.71 | -0.57 | -1.28 | Y | -0.26 | -0.35 | -0.09 | - |
| BOLA1 | 1.49 | -0.75 | -2.24 | Y | 0.26 | 0.17 | -0.09 | - |
| SF3B1 | 1.37 | -0.71 | -2.08 | Y | -0.26 | -0.35 | -0.09 | - |
| SNRNP35 | 0.86 | -0.93 | -1.79 | Y | 0.41 | 0.32 | -0.09 | - |
| NEIL1 | 1.13 | -0.39 | -1.52 | Y | -0.33 | -0.42 | -0.09 | - |
| PRKACA | 1.12 | -0.01 | -1.14 | Y | 0.92 | 0.83 | -0.09 | - |
| MFN2 | 1.13 | -0.22 | -1.35 | Y | 0.16 | 0.07 | -0.10 | - |
| ARAP1 | 1.32 | -0.59 | -1.91 | Y | -0.22 | -0.32 | -0.10 | - |
| ZNF26 | 1.44 | -0.06 | -1.50 | Y | 0.60 | 0.50 | -0.10 | - |
| RNF137 | -0.16 | -1.19 | -1.02 | Y | -0.35 | -0.45 | -0.10 | - |
| TAGLN2 | 1.46 | -0.65 | -2.11 | Y | -0.16 | -0.26 | -0.10 | - |
| TRIM22 | 1.71 | 0.36 | -1.35 | Y | -0.27 | -0.38 | -0.11 | - |
| ARRDC4 | 1.07 | -0.31 | -1.39 | Y | -0.43 | -0.54 | -0.11 | - |
| HSD17B10 | 1.14 | -0.31 | -1.45 | Y | 0.19 | 0.08 | -0.11 | - |
| MCM6 | 1.10 | -0.41 | -1.51 | Y | 0.73 | 0.61 | -0.12 | - |
| MYCBP | 1.32 | -0.62 | -1.94 | Y | 0.03 | -0.09 | -0.12 | - |
| SMAP2 | 0.69 | -1.17 | -1.87 | Y | 0.28 | 0.15 | -0.12 | - |
| MYO3A | -0.25 | -1.30 | -1.04 | Y | -0.41 | -0.54 | -0.12 | - |
| ACOT9 | 1.33 | 0.11 | -1.21 | Y | -0.48 | -0.60 | -0.13 | - |
| PABPC1 | 1.57 | -0.40 | -1.97 | Y | 0.41 | 0.27 | -0.14 | - |
| BRD2 | 1.73 | 0.12 | -1.61 | Y | 1.82 | 1.68 | -0.14 | - |
| SLC25A16 | 0.84 | -0.53 | -1.37 | Y | -0.65 | -0.79 | -0.14 | - |
| TCEA3 | 1.08 | -0.25 | -1.33 | Y | 0.60 | 0.46 | -0.14 | - |
| RBBP8 | 1.53 | -0.18 | -1.71 | Y | -0.46 | -0.61 | -0.14 | - |
| NFATC2IP | 0.41 | -1.25 | -1.66 | Y | -0.04 | -0.18 | -0.15 | - |
| PSMC5 | 2.61 | 1.67 | -0.93 | Y | 0.36 | 0.21 | -0.15 | - |
| SPG11 | 1.55 | 0.11 | -1.44 | Y | -0.97 | -1.13 | -0.15 | - |
| UBE2I | 1.46 | -0.12 | -1.58 | Y | 0.27 | 0.11 | -0.16 | - |
| ZNF695 | 0.36 | -0.93 | -1.30 | Y | 0.54 | 0.37 | -0.17 | - |
| CPNE3 | 0.28 | -0.68 | -0.96 | Y | -0.12 | -0.29 | -0.17 | - |
| TWF2 | 1.71 | 0.37 | -1.33 | Y | -0.07 | -0.24 | -0.18 | - |
| CDK5 | 0.54 | -0.92 | -1.46 | Y | -0.30 | -0.48 | -0.18 | - |
| CRIP1 | 1.86 | -0.95 | -2.81 | Y | 0.03 | -0.14 | -0.18 | - |
| EIF4A3 | 1.93 | -0.25 | -2.18 | Y | 0.38 | 0.20 | -0.18 | - |
| POLR2J | 1.12 | -0.92 | -2.04 | Y | 0.76 | 0.58 | -0.18 | - |
| MRPL42 | 0.94 | -0.26 | -1.20 | Y | 0.05 | -0.13 | -0.19 | - |
| MRPS5 | 0.41 | -0.48 | -0.89 | Y | -0.32 | -0.51 | -0.19 | - |
| TBX3 | 1.39 | 0.11 | -1.28 | Y | -0.01 | -0.20 | -0.19 | - |
| CSNK1A1 | 1.11 | -0.24 | -1.35 | Y | 0.51 | 0.32 | -0.19 | - |
| TMED10 | 1.07 | -0.96 | -2.03 | Y | -0.32 | -0.51 | -0.19 | - |
| PLEKHA4 | 1.34 | 0.25 | -1.10 | Y | 0.06 | -0.14 | -0.19 | - |
| FBL | 1.23 | 0.15 | -1.07 | Y | 0.17 | -0.02 | -0.19 | - |
| FXYD3 | 0.99 | 0.07 | -0.92 | Y | 0.70 | 0.51 | -0.20 | - |
| HMGB2 | 1.36 | -0.91 | -2.27 | Y | 0.45 | 0.25 | -0.20 | - |
| MRPL43 | 0.42 | -0.60 | -1.02 | Y | -0.38 | -0.58 | -0.20 | - |
| SMARCC1 | 2.61 | 1.09 | -1.52 | Y | 1.33 | 1.12 | -0.21 | - |
| RNASE11 | 0.86 | -0.26 | -1.12 | Y | -0.16 | -0.38 | -0.21 | - |
| LOC729991-MEF2B | 0.87 | -0.74 | -1.61 | Y | 0.62 | 0.40 | -0.22 | - |
| SOD1 | 1.27 | -0.39 | -1.66 | Y | -0.04 | -0.26 | -0.22 | - |
| RNF110 | 1.14 | -0.45 | -1.59 | Y | -0.08 | -0.31 | -0.23 | - |
| RET | 0.92 | -1.37 | -2.29 | Y | 0.62 | 0.39 | -0.23 | - |
| ZSCAN18 | 1.12 | -0.15 | -1.27 | Y | 1.32 | 1.08 | -0.23 | - |
| DHX30 | 0.72 | -0.77 | -1.48 | Y | -0.52 | -0.76 | -0.23 | - |
| IREB2 | 1.18 | 0.03 | -1.15 | Y | -0.72 | -0.96 | -0.24 | - |
| ZCWPW1 | 3.06 | -0.73 | -3.78 | Y | 0.92 | 0.69 | -0.24 | - |
| KIAA0669 | 1.29 | 0.36 | -0.93 | Y | 0.27 | 0.03 | -0.24 | - |
| EIF2AK1 | 1.96 | 0.11 | -1.84 | Y | 0.03 | -0.21 | -0.24 | - |
| AHNAK | 0.90 | -0.11 | -1.01 | Y | 0.57 | 0.32 | -0.24 | - |
| ACSS2 | 1.12 | -0.35 | -1.47 | Y | -0.41 | -0.65 | -0.24 | - |
| CAMKV | 2.67 | -0.75 | -3.43 | Y | 0.04 | -0.21 | -0.24 | - |
| ZBTB7B | 1.15 | -0.87 | -2.03 | Y | -0.02 | -0.26 | -0.24 | - |
| RAB5B | 0.37 | -0.54 | -0.91 | Y | -0.18 | -0.42 | -0.24 | - |
| IFI6 | 1.03 | -0.11 | -1.14 | Y | 0.24 | 0.00 | -0.24 | - |
| ATPAF2 | 0.85 | -0.32 | -1.17 | Y | -0.23 | -0.48 | -0.25 | - |
| CDK5RAP1 | 1.03 | -0.81 | -1.84 | Y | 0.29 | 0.04 | -0.25 | - |
| RPA4 | 0.77 | -0.60 | -1.37 | Y | 0.01 | -0.24 | -0.26 | - |
| NPM2 | 1.82 | 0.47 | -1.34 | Y | -0.04 | -0.30 | -0.26 | - |
| PNKP | 1.46 | -0.60 | -2.07 | Y | -0.68 | -0.95 | -0.27 | - |
| PAF1 | 0.53 | -0.41 | -0.94 | Y | -0.24 | -0.51 | -0.27 | - |
| STK32A | 0.67 | -0.36 | -1.03 | Y | -0.65 | -0.93 | -0.28 | - |
| CPSF2 | 0.77 | -0.23 | -0.99 | Y | 0.15 | -0.13 | -0.28 | - |
| PEX26 | 1.24 | -0.14 | -1.38 | Y | 0.88 | 0.60 | -0.28 | - |
| HIF1AN | 0.86 | -0.13 | -0.99 | Y | -0.16 | -0.45 | -0.29 | - |
| TCF12 | 1.31 | 0.32 | -0.99 | Y | 0.32 | 0.03 | -0.29 | - |
| VGLL2 | 1.59 | -0.13 | -1.72 | Y | 0.51 | 0.22 | -0.29 | - |
| FGF18 | 0.69 | -0.43 | -1.13 | Y | 0.96 | 0.68 | -0.29 | - |
| CENPB | 2.47 | -0.27 | -2.74 | Y | 1.29 | 0.98 | -0.31 | - |
| ZNF692 | 2.17 | 0.64 | -1.54 | Y | 2.23 | 1.92 | -0.31 | - |
| EIF2AK2 | 3.26 | -0.58 | -3.85 | Y | -0.41 | -0.72 | -0.31 | - |
| ZNF593 | 1.35 | -0.02 | -1.36 | Y | 1.25 | 0.94 | -0.32 | - |
| SUB1 | 1.52 | 0.08 | -1.44 | Y | 0.06 | -0.26 | -0.32 | - |
| ID1 | 1.64 | 0.61 | -1.03 | Y | 0.79 | 0.47 | -0.32 | - |
| RPN1 | 1.38 | -0.20 | -1.57 | Y | 0.17 | -0.16 | -0.33 | - |
| RELB | 3.34 | 1.01 | -2.33 | Y | 0.78 | 0.45 | -0.33 | - |
| SMCR7L | 0.17 | -0.83 | -1.00 | Y | 0.55 | 0.21 | -0.33 | - |
| ARID3B | 0.89 | -0.09 | -0.98 | Y | 1.01 | 0.66 | -0.35 | - |
| MAGEA12 | 1.59 | -0.80 | -2.39 | Y | 0.04 | -0.31 | -0.35 | - |
| DCAF7 | 1.40 | -0.87 | -2.27 | Y | 0.89 | 0.53 | -0.36 | - |
| FLJ31413 | 0.95 | 0.03 | -0.92 | Y | 0.02 | -0.34 | -0.36 | - |
| CCBL2 | 0.47 | -0.49 | -0.96 | Y | 1.11 | 0.75 | -0.37 | - |
| APTX | 1.19 | -0.37 | -1.55 | Y | 0.30 | -0.07 | -0.37 | - |
| ATRIP | 1.25 | -0.53 | -1.78 | Y | 1.58 | 1.20 | -0.38 | - |
| SCYL1 | 1.08 | -0.30 | -1.37 | Y | 0.16 | -0.22 | -0.39 | - |
| LOC51270 | 2.82 | -1.00 | -3.81 | Y | 0.90 | 0.51 | -0.39 | - |
| NTRK2 | 0.46 | -0.75 | -1.21 | Y | 0.00 | -0.39 | -0.39 | - |
| MLLT3 | 0.47 | -0.48 | -0.94 | Y | 0.78 | 0.38 | -0.40 | - |
| ZMYM5 | 1.59 | -0.33 | -1.92 | Y | 1.91 | 1.51 | -0.40 | - |
| NRBP2 | 1.42 | 0.46 | -0.96 | Y | -0.07 | -0.48 | -0.41 | - |
| SDR39U1 | 0.17 | -0.80 | -0.97 | Y | 1.73 | 1.32 | -0.41 | - |
| CKMT2 | 1.32 | -0.79 | -2.11 | Y | 1.01 | 0.60 | -0.41 | - |
| PPIE | 0.64 | -0.90 | -1.54 | Y | -0.04 | -0.46 | -0.42 | - |
| RFC3 | 1.11 | -0.44 | -1.55 | Y | 0.54 | 0.13 | -0.42 | - |
| RALY | 1.45 | -0.16 | -1.61 | Y | 0.34 | -0.08 | -0.42 | - |
| GLYAT | 1.48 | -0.46 | -1.94 | Y | 0.15 | -0.28 | -0.42 | - |
| MARK2 | 1.44 | -0.13 | -1.57 | Y | 0.04 | -0.39 | -0.43 | - |
| STAT1 | 1.46 | 0.12 | -1.34 | Y | -0.05 | -0.48 | -0.43 | - |
| SCMH1 | 1.64 | 0.13 | -1.50 | Y | -0.53 | -0.96 | -0.44 | - |
| ZNF426 | -0.03 | -0.93 | -0.90 | Y | -0.04 | -0.48 | -0.44 | - |
| SIRT5 | 2.75 | 0.42 | -2.32 | Y | 0.03 | -0.41 | -0.44 | - |
| ZNF69 | 0.50 | -0.41 | -0.91 | Y | 0.14 | -0.30 | -0.44 | - |
| FUBP1 | 1.92 | 0.36 | -1.56 | Y | 1.20 | 0.76 | -0.44 | - |
| NDUFAF1 | 1.27 | -0.18 | -1.45 | Y | -0.24 | -0.68 | -0.44 | - |
| EWSR1 | 2.90 | 0.31 | -2.60 | Y | 0.96 | 0.51 | -0.45 | - |
| HDAC8 | 0.45 | -0.63 | -1.08 | Y | 0.24 | -0.21 | -0.45 | - |
| ATF7 | 1.06 | -0.08 | -1.13 | Y | -0.10 | -0.55 | -0.46 | - |
| MLF2 | 0.67 | -0.60 | -1.26 | Y | 0.06 | -0.40 | -0.46 | - |
| TRAP1 | 0.93 | 0.03 | -0.90 | Y | 0.07 | -0.39 | -0.46 | - |
| CHRAC1 | 1.11 | -0.67 | -1.79 | Y | 0.05 | -0.41 | -0.47 | - |
| CCM2 | 2.14 | 0.11 | -2.03 | Y | -0.11 | -0.58 | -0.47 | - |
| SETMAR | 1.40 | -0.04 | -1.45 | Y | -0.23 | -0.70 | -0.47 | - |
| DCI | 1.87 | -1.08 | -2.95 | Y | -0.07 | -0.55 | -0.48 | - |
| SURF4 | 2.60 | 0.49 | -2.11 | Y | -0.09 | -0.57 | -0.48 | - |
| MXD4 | 0.11 | -1.30 | -1.41 | Y | 0.20 | -0.28 | -0.48 | - |
| ZNF79 | 1.02 | -0.85 | -1.87 | Y | -0.13 | -0.61 | -0.48 | - |
| ZNF484 | 1.28 | -0.40 | -1.69 | Y | 0.97 | 0.48 | -0.49 | - |
| ZNF606 | 1.20 | 0.28 | -0.92 | Y | 0.88 | 0.39 | -0.49 | - |
| PAWR | 1.27 | -0.77 | -2.04 | Y | 0.25 | -0.25 | -0.49 | - |
| EPHA4 | 0.52 | -0.43 | -0.95 | Y | -0.20 | -0.69 | -0.49 | - |
| PFDN1 | 0.97 | 0.00 | -0.96 | Y | 0.43 | -0.07 | -0.50 | - |
| RARG | 0.92 | -0.09 | -1.01 | Y | 0.04 | -0.46 | -0.50 | - |
| HES4 | 0.97 | -0.52 | -1.49 | Y | -0.29 | -0.78 | -0.50 | - |
| VCY | 1.39 | 0.26 | -1.13 | Y | 0.84 | 0.34 | -0.50 | - |
| ZNF155 | 0.88 | -0.32 | -1.20 | Y | -0.18 | -0.69 | -0.50 | - |
| ZBTB6 | 1.34 | -0.37 | -1.71 | Y | 0.38 | -0.12 | -0.51 | - |
| NR1H3 | 0.66 | -0.34 | -1.00 | Y | 0.53 | 0.02 | -0.51 | - |
| RCOR2 | 1.85 | -0.26 | -2.12 | Y | 0.18 | -0.33 | -0.52 | - |
| HSBP1 | 1.98 | -0.62 | -2.60 | Y | -0.06 | -0.58 | -0.52 | - |
| PLK1S1 | 2.42 | 0.51 | -1.91 | Y | 4.79 | 4.27 | -0.52 | - |
| PRKRA | 1.94 | -0.38 | -2.32 | Y | 0.49 | -0.04 | -0.53 | - |
| NCL | 1.75 | 0.86 | -0.89 | Y | 0.50 | -0.03 | -0.54 | - |
| MLX | 3.20 | 0.22 | -2.98 | Y | 1.49 | 0.95 | -0.54 | - |
| SGK494 | 0.97 | -0.56 | -1.53 | Y | 0.99 | 0.45 | -0.54 | - |
| TRIM9 | 1.78 | -0.20 | -1.98 | Y | 1.10 | 0.55 | -0.55 | - |
| MLKL | 0.71 | -0.28 | -1.00 | Y | 0.46 | -0.09 | -0.55 | - |
| PTPMT1 | 1.65 | 0.06 | -1.59 | Y | 0.70 | 0.15 | -0.55 | - |
| MORF4L1 | 0.82 | -0.30 | -1.12 | Y | 1.10 | 0.54 | -0.56 | - |
| TCEB3B | 2.23 | -0.86 | -3.09 | Y | 0.24 | -0.32 | -0.56 | - |
| MRPS36 | 0.90 | -0.15 | -1.05 | Y | 0.48 | -0.09 | -0.57 | - |
| SFRS1 | 2.76 | 0.77 | -1.99 | Y | 1.06 | 0.49 | -0.57 | - |
| SRA1 | 1.03 | -0.69 | -1.72 | Y | 0.92 | 0.35 | -0.57 | - |
| GRHPR | 1.49 | -0.13 | -1.62 | Y | 1.24 | 0.67 | -0.57 | - |
| BRD9 | 1.40 | 0.46 | -0.94 | Y | 1.41 | 0.84 | -0.57 | - |
| NAT8B | 1.45 | 0.52 | -0.94 | Y | 0.36 | -0.21 | -0.57 | - |
| CHEK1 | 3.74 | 1.05 | -2.69 | Y | 2.22 | 1.65 | -0.57 | - |
| UQCRC2 | 1.64 | -0.97 | -2.61 | Y | 0.60 | 0.03 | -0.57 | - |
| PHF17 | 1.90 | -1.00 | -2.90 | Y | 0.70 | 0.12 | -0.58 | - |
| IL17B | 0.42 | -1.46 | -1.88 | Y | 0.30 | -0.29 | -0.59 | - |
| FAM164C | 2.08 | 0.41 | -1.67 | Y | 0.04 | -0.55 | -0.59 | - |
| NCAPG | 2.05 | -0.28 | -2.33 | Y | -0.37 | -0.96 | -0.59 | - |
| MRPS7 | 1.28 | -0.92 | -2.21 | Y | -0.02 | -0.61 | -0.59 | - |
| HEXIM2 | 2.12 | -0.29 | -2.41 | Y | -0.17 | -0.78 | -0.61 | - |
| EXOSC1 | 1.32 | 0.02 | -1.29 | Y | 0.86 | 0.25 | -0.61 | - |
| TRIM27 | 0.52 | -0.92 | -1.44 | Y | 0.52 | -0.10 | -0.61 | - |
| ENC1 | 0.94 | -0.31 | -1.25 | Y | 0.48 | -0.13 | -0.61 | - |
| PPP2R1B | 1.22 | 0.27 | -0.94 | Y | 0.65 | 0.03 | -0.63 | - |
| ID3 | 1.39 | 0.49 | -0.90 | Y | 0.62 | -0.01 | -0.63 | - |
| STAT4 | 1.26 | 0.03 | -1.24 | Y | 0.85 | 0.22 | -0.63 | - |
| FGF13 | 1.62 | -0.29 | -1.92 | Y | 0.01 | -0.63 | -0.64 | - |
| XRCC6BP1 | 1.34 | -0.38 | -1.72 | Y | 0.39 | -0.25 | -0.64 | - |
| PBF | 0.71 | -0.18 | -0.89 | Y | 0.15 | -0.49 | -0.64 | - |
| CD8A | 0.02 | -1.09 | -1.11 | Y | 0.01 | -0.63 | -0.64 | - |
| ACTG1 | 1.35 | 0.06 | -1.29 | Y | 0.22 | -0.43 | -0.64 | - |
| GPATCH1 | 0.24 | -0.99 | -1.23 | Y | 0.29 | -0.36 | -0.65 | - |
| KLHL14 | 1.90 | -0.23 | -2.13 | Y | 0.83 | 0.18 | -0.65 | - |
| GTF3C2 | 0.35 | -1.12 | -1.47 | Y | 0.10 | -0.55 | -0.66 | - |
| MAGOHB | 1.34 | 0.35 | -1.00 | Y | 0.50 | -0.16 | -0.66 | - |
| HDGFL1 | 0.85 | -0.25 | -1.10 | Y | 0.90 | 0.24 | -0.67 | - |
| SOX6 | 1.04 | -0.22 | -1.26 | Y | -0.21 | -0.88 | -0.67 | - |
| PACSIN1 | 1.91 | 0.11 | -1.80 | Y | 0.64 | -0.04 | -0.68 | - |
| MAGEB1 | 3.14 | -0.05 | -3.19 | Y | 2.73 | 2.05 | -0.68 | - |
| EIF3E | 1.12 | -0.38 | -1.49 | Y | 0.14 | -0.55 | -0.68 | - |
| ARID3A | 3.09 | 1.17 | -1.92 | Y | 0.68 | -0.01 | -0.69 | - |
| CAMKK1 | 1.29 | 0.08 | -1.21 | Y | 1.84 | 1.15 | -0.69 | - |
| METTL4 | 0.89 | -0.82 | -1.71 | Y | 0.57 | -0.12 | -0.69 | - |
| SFRS7 | 0.99 | -0.11 | -1.09 | Y | 0.77 | 0.08 | -0.69 | - |
| PRDX1 | 1.35 | -0.37 | -1.71 | Y | 0.23 | -0.47 | -0.70 | - |
| RBM8A | 2.13 | 0.99 | -1.15 | Y | 0.35 | -0.35 | -0.70 | - |
| ZNF449 | 0.74 | -0.71 | -1.44 | Y | 1.31 | 0.60 | -0.71 | - |
| CCNC | 1.17 | 0.06 | -1.12 | Y | 0.39 | -0.32 | -0.72 | - |
| TRAT1 | 0.28 | -0.84 | -1.12 | Y | 0.14 | -0.58 | -0.72 | - |
| LETM1 | 1.21 | -0.61 | -1.82 | Y | 0.82 | 0.10 | -0.72 | - |
| COX11 | 0.89 | -0.72 | -1.61 | Y | 0.30 | -0.42 | -0.72 | - |
| GTF2H5 | 0.73 | -0.25 | -0.98 | Y | 0.18 | -0.54 | -0.73 | - |
| ZXDC | 1.56 | -0.07 | -1.63 | Y | 0.17 | -0.55 | -0.73 | - |
| MYLK2 | 1.75 | -0.18 | -1.92 | Y | 0.90 | 0.18 | -0.73 | - |
| STAT6 | 0.08 | -1.10 | -1.18 | Y | 0.64 | -0.10 | -0.74 | - |
| RNF2 | 1.28 | -0.49 | -1.78 | Y | 0.32 | -0.42 | -0.74 | - |
| ZNF496 | 1.44 | -0.19 | -1.63 | Y | 0.07 | -0.68 | -0.74 | - |
| HIRIP3 | 4.58 | 0.80 | -3.77 | Y | 4.85 | 4.10 | -0.75 | - |
| POU6F1 | 0.86 | -0.50 | -1.36 | Y | 0.24 | -0.51 | -0.75 | - |
| TRIP13 | 1.49 | -0.59 | -2.07 | Y | 0.08 | -0.68 | -0.76 | - |
| NRP2 | 0.80 | -0.73 | -1.53 | Y | 0.62 | -0.14 | -0.76 | - |
| FGFR2 | 1.59 | -0.21 | -1.80 | Y | 1.02 | 0.26 | -0.76 | - |
| HAGH | 2.06 | 0.42 | -1.64 | Y | 0.23 | -0.54 | -0.77 | - |
| CSRP2 | 1.13 | 0.08 | -1.05 | Y | 0.66 | -0.12 | -0.77 | - |
| PSMC3 | 1.41 | 0.17 | -1.24 | Y | 0.61 | -0.17 | -0.78 | - |
| PRKX | 1.69 | -0.13 | -1.82 | Y | 1.12 | 0.34 | -0.78 | - |
| SLC25A19 | 1.36 | -0.38 | -1.74 | Y | -0.03 | -0.81 | -0.78 | - |
| FIS1 | 1.77 | 0.02 | -1.75 | Y | 1.09 | 0.31 | -0.78 | - |
| NFKB1 | 0.99 | 0.00 | -0.99 | Y | 1.65 | 0.86 | -0.79 | - |
| THAP1 | 1.22 | 0.06 | -1.16 | Y | 1.02 | 0.23 | -0.79 | - |
| PABPC5 | 1.57 | -0.66 | -2.22 | Y | 0.44 | -0.35 | -0.80 | - |
| DAXX | 3.68 | -0.44 | -4.12 | Y | 2.25 | 1.45 | -0.80 | - |
| E2F2 | 1.87 | -0.13 | -2.00 | Y | 0.40 | -0.41 | -0.81 | - |
| ESR1 | 1.70 | -0.14 | -1.84 | Y | -0.20 | -1.02 | -0.82 | - |
| RIOK1 | 4.34 | 1.42 | -2.91 | Y | 1.35 | 0.53 | -0.82 | - |
| USP39 | 1.58 | -0.99 | -2.57 | Y | 1.73 | 0.91 | -0.83 | - |
| SUV420H1 | 1.01 | -0.66 | -1.67 | Y | 0.80 | -0.03 | -0.83 | - |
| ARHGEF5 | 1.46 | -0.48 | -1.95 | Y | 1.14 | 0.30 | -0.84 | - |
| PRRG2 | 0.65 | -1.43 | -2.08 | Y | 0.79 | -0.07 | -0.85 | - |
| C19orf25 | 1.03 | -0.41 | -1.44 | Y | 0.64 | -0.22 | -0.86 | - |
| POLR1C | 1.38 | 0.49 | -0.88 | Y | 0.40 | -0.46 | -0.86 | - |
| ZDHHC3 | 2.48 | -0.87 | -3.34 | Y | -0.42 | -1.28 | -0.86 | - |
| ANP32A | 0.04 | -0.92 | -0.97 | Y | 1.03 | 0.17 | -0.87 | - |
| ZNF213 | 1.06 | -0.60 | -1.66 | Y | 0.50 | -0.37 | -0.87 | - |
| ANKHD1 | 2.87 | 0.74 | -2.13 | Y | 3.21 | 2.34 | -0.87 | - |
| THAP5 | 1.61 | -0.01 | -1.62 | Y | 0.49 | -0.40 | -0.88 | - |
| BCL3 | 1.96 | 0.02 | -1.94 | Y | 0.08 | -0.81 | -0.89 | - |
| RING1 | 0.59 | -0.39 | -0.98 | Y | 1.28 | 0.39 | -0.89 | - |
| PRMT8 | 0.36 | -0.76 | -1.12 | Y | 0.69 | -0.20 | -0.89 | - |
| ANXA5 | 2.03 | 0.16 | -1.87 | Y | 0.37 | -0.52 | -0.90 | - |
| DHX32 | 1.06 | -0.70 | -1.76 | Y | 0.74 | -0.16 | -0.90 | - |
| NPM3 | 3.39 | 0.29 | -3.10 | Y | 0.84 | -0.06 | -0.91 | - |
| EEF1B2 | 0.95 | -0.03 | -0.98 | Y | 0.48 | -0.43 | -0.91 | - |
| EIF2B5 | 2.64 | 1.61 | -1.02 | Y | 0.80 | -0.11 | -0.91 | - |
| ASAP3 | 4.27 | 0.62 | -3.65 | Y | 1.04 | 0.13 | -0.92 | - |
| KCNIP1 | 1.72 | -0.29 | -2.01 | Y | 0.61 | -0.31 | -0.92 | - |
| CD9 | 1.31 | -0.21 | -1.52 | Y | 1.01 | 0.07 | -0.93 | - |
| RNF113A | 2.74 | -0.46 | -3.20 | Y | 0.71 | -0.23 | -0.95 | - |
| CTBP1 | 1.71 | -0.54 | -2.25 | Y | 0.67 | -0.29 | -0.95 | - |
| ISL1 | 0.30 | -0.59 | -0.89 | Y | 1.61 | 0.65 | -0.96 | - |
| MRPL36 | 1.02 | -0.73 | -1.75 | Y | 0.14 | -0.82 | -0.96 | - |
| ARNT | 1.65 | -0.36 | -2.00 | Y | 1.56 | 0.58 | -0.98 | - |
| AK1 | 1.99 | -0.31 | -2.30 | Y | 0.96 | -0.02 | -0.98 | - |
| ABCB7 | 0.88 | -0.07 | -0.95 | Y | 0.61 | -0.38 | -0.98 | - |
| PER3 | 0.44 | -1.03 | -1.47 | Y | 0.74 | -0.26 | -0.99 | - |
| THAP6 | 0.27 | -0.96 | -1.23 | Y | 0.79 | -0.21 | -1.00 | - |
| APOBEC2 | 0.89 | -0.13 | -1.03 | Y | 1.38 | 0.37 | -1.00 | - |
| CRABP2 | 0.66 | -1.57 | -2.22 | Y | 0.06 | -0.96 | -1.02 | - |
| PMP2 | 1.26 | -0.26 | -1.52 | Y | 0.84 | -0.18 | -1.02 | - |
| LHFP | 0.67 | -0.74 | -1.41 | Y | 0.51 | -0.51 | -1.02 | - |
| HNRNPL | 0.83 | -0.51 | -1.33 | Y | 0.88 | -0.16 | -1.03 | - |
| PRDM7 | 1.35 | -0.28 | -1.63 | Y | 0.86 | -0.18 | -1.05 | - |
| DDX21 | 1.91 | -0.57 | -2.48 | Y | 1.13 | 0.06 | -1.07 | - |
| MRM1 | 1.51 | 0.54 | -0.97 | Y | 0.02 | -1.06 | -1.08 | - |
| TAPBP | 0.38 | -0.54 | -0.92 | Y | 0.44 | -0.65 | -1.09 | - |
| TIMM17B | 0.24 | -0.67 | -0.91 | Y | 0.19 | -0.91 | -1.09 | - |
| RIOK3 | 3.68 | 1.53 | -2.15 | Y | 2.69 | 1.58 | -1.11 | - |
| BNIP3 | 0.64 | -0.32 | -0.95 | Y | 0.26 | -0.87 | -1.13 | - |
| WBP11 | 1.90 | 0.29 | -1.61 | Y | 1.43 | 0.30 | -1.13 | - |
| TBX6 | 0.71 | -0.52 | -1.23 | Y | 0.22 | -0.93 | -1.15 | - |
| ZNF35 | 2.09 | 0.26 | -1.83 | Y | 0.31 | -0.87 | -1.18 | - |
| PHF8 | 0.19 | -0.75 | -0.94 | Y | 0.83 | -0.36 | -1.19 | - |
| CHERP | 1.60 | -0.33 | -1.94 | Y | 1.14 | -0.06 | -1.20 | - |
| EIF3G | 0.25 | -0.80 | -1.05 | Y | 0.52 | -0.68 | -1.20 | - |
| BCAS2 | 0.44 | -0.54 | -0.98 | Y | 0.59 | -0.62 | -1.21 | - |
| PEX16 | 1.17 | 0.09 | -1.08 | Y | 3.34 | 2.14 | -1.21 | - |
| PSD | 0.33 | -1.36 | -1.69 | Y | 1.77 | 0.54 | -1.22 | - |
| RAB18 | 0.76 | -0.79 | -1.55 | Y | 0.80 | -0.43 | -1.23 | - |
| POGK | 2.83 | 0.09 | -2.74 | Y | 0.87 | -0.35 | -1.23 | - |
| KLHL29 | 0.51 | -0.48 | -0.99 | Y | 1.12 | -0.13 | -1.24 | - |
| MRPL12 | 1.31 | 0.01 | -1.31 | Y | 1.38 | 0.14 | -1.25 | - |
| MRPL40 | 1.29 | 0.35 | -0.94 | Y | 0.30 | -0.95 | -1.26 | - |
| TRIM69 | 1.11 | -0.55 | -1.66 | Y | 1.45 | 0.19 | -1.26 | - |
| SLC38A2 | 2.14 | 0.29 | -1.85 | Y | 0.52 | -0.75 | -1.27 | - |
| XRCC2 | 1.62 | -0.36 | -1.98 | Y | 0.67 | -0.60 | -1.27 | - |
| POLR3D | 1.15 | -0.12 | -1.27 | Y | 0.56 | -0.72 | -1.28 | - |
| EIF2B2 | 1.15 | -0.42 | -1.57 | Y | 1.04 | -0.25 | -1.28 | - |
| KLHL36 | 0.64 | -0.63 | -1.28 | Y | 0.91 | -0.38 | -1.29 | - |
| MRPL21 | 0.62 | -1.25 | -1.87 | Y | 1.03 | -0.30 | -1.34 | - |
| THUMPD1 | 2.76 | 0.86 | -1.90 | Y | 1.61 | 0.27 | -1.34 | - |
| ZC3H7A | 0.66 | -0.48 | -1.14 | Y | 0.38 | -0.97 | -1.34 | - |
| HES1 | 1.21 | -0.41 | -1.62 | Y | 0.73 | -0.63 | -1.37 | - |
| ATF6 | 1.79 | 0.50 | -1.29 | Y | 1.57 | 0.19 | -1.38 | - |
| POLR2D | 0.83 | -0.33 | -1.16 | Y | 1.07 | -0.34 | -1.41 | - |
| HSPE1 | 0.69 | -0.45 | -1.13 | Y | 1.12 | -0.30 | -1.43 | - |
| TADA3 | 0.70 | -1.07 | -1.77 | Y | 0.30 | -1.14 | -1.44 | - |
| TCFL5 | 1.11 | 0.06 | -1.05 | Y | 1.40 | -0.06 | -1.46 | - |
| GCFC1 | 1.87 | 0.13 | -1.74 | Y | 0.38 | -1.10 | -1.48 | - |
| RBM22 | 0.42 | -1.12 | -1.54 | Y | 0.91 | -0.58 | -1.49 | - |
| ELK3 | 1.66 | -0.21 | -1.87 | Y | 0.53 | -0.99 | -1.52 | - |
| IOH4528 | 0.41 | -0.57 | -0.97 | Y | 0.66 | -0.86 | -1.52 | - |
| JMJD2A | 1.35 | -0.46 | -1.81 | Y | 0.44 | -1.08 | -1.52 | - |
| ACSL6 | 1.39 | 0.14 | -1.24 | Y | 1.19 | -0.34 | -1.53 | - |
| CBX7 | 1.07 | -0.40 | -1.47 | Y | 1.54 | 0.01 | -1.53 | - |
| MBP | 0.93 | -0.36 | -1.29 | Y | 1.31 | -0.23 | -1.53 | - |
| POP7 | 0.75 | -0.77 | -1.52 | Y | 0.96 | -0.57 | -1.54 | - |
| AKR1A1 | 1.15 | -0.72 | -1.87 | Y | 0.13 | -1.43 | -1.56 | - |
| ZDHHC7 | 1.33 | 0.38 | -0.95 | Y | 1.41 | -0.15 | -1.56 | - |
| PPP5C | 0.43 | -0.56 | -0.99 | Y | 0.87 | -0.74 | -1.60 | - |
| ANKRD1 | 0.55 | -0.64 | -1.20 | Y | 1.31 | -0.33 | -1.63 | - |
| C5orf24 | 1.33 | 0.32 | -1.01 | Y | 0.53 | -1.11 | -1.64 | - |
| MRPS2 | 0.97 | 0.08 | -0.90 | Y | 0.80 | -0.85 | -1.66 | - |
| SNRPE | 0.80 | -0.86 | -1.65 | Y | -0.04 | -1.72 | -1.68 | - |
| ACO1 | 0.13 | -0.97 | -1.10 | Y | 1.35 | -0.33 | -1.68 | - |
| CCDC25 | 1.41 | 0.26 | -1.15 | Y | 0.68 | -1.01 | -1.68 | - |
| MRPL20 | 0.51 | -0.46 | -0.98 | Y | 0.72 | -0.98 | -1.70 | - |
| GATM | 1.21 | -0.28 | -1.49 | Y | 1.72 | -0.03 | -1.74 | - |
| TAF6 | 1.12 | 0.23 | -0.89 | Y | 0.92 | -0.85 | -1.77 | - |
| HEAB | 1.21 | 0.03 | -1.18 | Y | 0.57 | -1.25 | -1.82 | - |
| NCAPH | 1.18 | -0.52 | -1.70 | Y | 0.71 | -1.15 | -1.86 | - |
| SFRS16 | 1.70 | -0.43 | -2.13 | Y | 1.81 | -0.10 | -1.91 | - |
| NTRK3 | 0.96 | -0.07 | -1.03 | Y | 1.50 | -0.48 | -1.97 | - |
| MRPL11 | 1.79 | 0.52 | -1.27 | Y | 1.72 | -0.28 | -2.00 | - |
| BCL2L13 | 1.49 | -0.49 | -1.98 | Y | 2.14 | 0.13 | -2.01 | - |
| GRK6 | 2.11 | -0.24 | -2.35 | Y | 1.91 | -0.12 | -2.03 | - |
| NDUFB4 | 1.04 | -0.59 | -1.63 | Y | 0.83 | -1.26 | -2.09 | - |
| COX7A2L | 1.34 | -0.30 | -1.63 | Y | 1.75 | -0.43 | -2.17 | - |
| TFPT | 0.92 | -0.21 | -1.13 | Y | 1.74 | -0.47 | -2.22 | - |
| COX6A2 | 0.27 | -0.70 | -0.98 | Y | 1.17 | -1.08 | -2.25 | - |
| RTN4IP1 | 0.94 | -0.09 | -1.02 | Y | 2.72 | 0.46 | -2.26 | - |
| ZCCHC8 | 2.08 | -0.01 | -2.09 | Y | 1.70 | -0.61 | -2.31 | - |
| TST | 1.71 | -1.17 | -2.88 | Y | 1.67 | -0.74 | -2.41 | - |
| BSG | 1.74 | -0.64 | -2.37 | Y | 2.04 | -0.39 | -2.43 | - |
| CCDC97 | 1.56 | 0.41 | -1.15 | Y | 2.25 | -0.26 | -2.51 | - |
| ZNF239 | 1.70 | -0.77 | -2.47 | Y | 1.72 | -0.80 | -2.52 | - |
| RAD54B | 2.79 | 0.03 | -2.76 | Y | 1.85 | -0.95 | -2.80 | - |
| RFX6 | 1.20 | 0.12 | -1.08 | Y | 2.41 | -0.51 | -2.92 | - |
| TAF15 | 3.73 | 0.48 | -3.25 | Y | 3.47 | -0.42 | -3.89 | - |
| MAP4K5 | 3.33 | 3.49 | 0.16 | - | 0.09 | 5.36 | 5.27 | Y |
| FRK | 2.08 | 2.92 | 0.84 | - | 0.37 | 4.91 | 4.54 | Y |
| CSNK1G1 | 3.33 | 3.92 | 0.59 | - | 1.34 | 5.55 | 4.20 | Y |
| LYN | 2.60 | 4.14 | 1.54 | - | 0.53 | 4.69 | 4.16 | Y |
| TMEM216 | 1.51 | 1.05 | -0.47 | - | -0.74 | 2.97 | 3.71 | Y |
| GYPA | 1.00 | 0.25 | -0.76 | - | -0.57 | 3.02 | 3.59 | Y |
| ZNF343 | 1.56 | 0.94 | -0.62 | - | -1.36 | 1.85 | 3.21 | Y |
| HCK | 3.23 | 2.65 | -0.58 | - | 2.50 | 5.64 | 3.14 | Y |
| PHAX | 0.96 | 1.34 | 0.37 | - | 0.43 | 3.48 | 3.05 | Y |
| C11orf13 | 0.09 | 1.16 | 1.07 | - | -0.76 | 2.18 | 2.95 | Y |
| YBX2 | 0.35 | 0.48 | 0.13 | - | -0.72 | 2.08 | 2.80 | Y |
| PTGES2 | -0.31 | -0.33 | -0.02 | - | -1.28 | 1.48 | 2.76 | Y |
| OXCT2 | -0.10 | -0.70 | -0.60 | - | -0.40 | 2.23 | 2.63 | Y |
| PDK4 | 0.70 | 0.53 | -0.17 | - | -0.79 | 1.82 | 2.61 | Y |
| DYRK1B | 1.01 | 0.82 | -0.19 | - | -0.78 | 1.80 | 2.59 | Y |
| MEIS3 | 1.55 | 1.24 | -0.30 | - | -0.11 | 2.45 | 2.56 | Y |
| CCDC127 | 1.70 | 1.08 | -0.62 | - | -0.29 | 2.18 | 2.47 | Y |
| ZNF501 | 1.67 | 0.96 | -0.71 | - | -0.06 | 2.34 | 2.40 | Y |
| PXK | 0.61 | 0.55 | -0.05 | - | -1.27 | 1.08 | 2.35 | Y |
| XRN1 | -0.20 | -0.85 | -0.65 | - | -0.93 | 1.41 | 2.35 | Y |
| MKRN2 | 0.24 | 0.92 | 0.68 | - | -0.52 | 1.80 | 2.32 | Y |
| CELF1 | 0.02 | -0.60 | -0.62 | - | 0.17 | 2.48 | 2.31 | Y |
| EXOG | 0.44 | -0.01 | -0.46 | - | -0.90 | 1.37 | 2.26 | Y |
| NAP1L2 | 0.50 | 0.89 | 0.39 | - | -0.14 | 2.12 | 2.25 | Y |
| CASQ1 | 0.82 | 0.14 | -0.68 | - | -0.29 | 1.96 | 2.25 | Y |
| OSGIN1 | -0.18 | -0.50 | -0.31 | - | -1.02 | 1.20 | 2.23 | Y |
| ZNF557 | 0.78 | 0.08 | -0.70 | - | -0.39 | 1.83 | 2.22 | Y |
| THRSP | -0.61 | -0.26 | 0.35 | - | -1.04 | 1.18 | 2.21 | Y |
| STAT5B | 1.15 | 1.14 | -0.01 | - | -0.49 | 1.72 | 2.21 | Y |
| CAMK2A | 1.63 | 1.36 | -0.27 | - | 0.76 | 2.93 | 2.18 | Y |
| C1orf115 | -0.43 | -0.26 | 0.17 | - | -1.16 | 0.98 | 2.14 | Y |
| ETV1 | 0.43 | 1.27 | 0.83 | - | -0.81 | 1.32 | 2.13 | Y |
| MKNK1 | 1.40 | 2.32 | 0.91 | - | 0.31 | 2.40 | 2.09 | Y |
| HSD11B1 | -0.15 | -1.01 | -0.86 | - | -1.30 | 0.78 | 2.09 | Y |
| POLR1B | -0.29 | 1.05 | 1.34 | - | -0.61 | 1.47 | 2.08 | Y |
| ADCK4 | -0.13 | 0.07 | 0.20 | - | -0.52 | 1.56 | 2.08 | Y |
| CREB1 | -0.11 | -0.63 | -0.52 | - | -0.61 | 1.47 | 2.07 | Y |
| NR1I3 | 0.19 | -0.24 | -0.44 | - | -0.44 | 1.64 | 2.07 | Y |
| BCAT2 | 0.63 | 0.21 | -0.42 | - | -1.19 | 0.87 | 2.06 | Y |
| CFL2 | 1.05 | 0.34 | -0.71 | - | -1.12 | 0.93 | 2.05 | Y |
| HR | 0.07 | -0.40 | -0.47 | - | -0.90 | 1.14 | 2.04 | Y |
| ELL2 | 1.57 | 1.58 | 0.01 | - | -0.28 | 1.73 | 2.01 | Y |
| VAMP4 | 0.55 | 0.30 | -0.25 | - | -0.69 | 1.31 | 2.00 | Y |
| ZNF641 | 1.07 | 0.36 | -0.71 | - | -0.92 | 1.08 | 1.99 | Y |
| PRKD2 | 0.53 | 0.41 | -0.12 | - | 0.62 | 2.61 | 1.99 | Y |
| CHEK2 | 2.16 | 3.79 | 1.63 | - | 0.11 | 2.09 | 1.98 | Y |
| FOXP4 | 0.21 | 0.14 | -0.07 | - | -0.46 | 1.49 | 1.95 | Y |
| MAGEA8 | 1.03 | 0.40 | -0.63 | - | -0.34 | 1.61 | 1.95 | Y |
| TOR1A | -0.81 | -0.77 | 0.04 | - | -1.14 | 0.81 | 1.95 | Y |
| TOMM7 | 0.35 | -0.38 | -0.73 | - | -0.91 | 1.03 | 1.93 | Y |
| CCND1 | -0.10 | 0.05 | 0.15 | - | -0.82 | 1.12 | 1.93 | Y |
| ACVR2B | 0.20 | 0.29 | 0.08 | - | -1.02 | 0.92 | 1.93 | Y |
| SENP3 | 0.46 | -0.37 | -0.83 | - | -1.01 | 0.91 | 1.92 | Y |
| ZNF330 | 1.59 | 2.00 | 0.41 | - | -1.55 | 0.35 | 1.91 | Y |
| TRIM32 | 1.64 | 1.38 | -0.26 | - | -1.20 | 0.66 | 1.86 | Y |
| XPA | 0.81 | 0.93 | 0.12 | - | 0.56 | 2.42 | 1.86 | Y |
| FAM104A | 1.06 | 0.48 | -0.58 | - | 0.16 | 2.02 | 1.86 | Y |
| RUVBL1 | 0.84 | 1.35 | 0.52 | - | -1.13 | 0.72 | 1.85 | Y |
| PABPC4 | -0.13 | -0.63 | -0.50 | - | -0.43 | 1.42 | 1.85 | Y |
| ACADL | -0.40 | 0.09 | 0.49 | - | -0.85 | 1.00 | 1.85 | Y |
| PTMA | 0.34 | 0.23 | -0.11 | - | -0.63 | 1.22 | 1.85 | Y |
| MRPL44 | 1.03 | 0.31 | -0.72 | - | 0.03 | 1.87 | 1.84 | Y |
| CRIP2 | 1.22 | 1.56 | 0.33 | - | -0.62 | 1.22 | 1.84 | Y |
| VDAC1 | 1.08 | 0.29 | -0.79 | - | -0.58 | 1.25 | 1.83 | Y |
| MBNL3 | 0.12 | 0.03 | -0.09 | - | -0.42 | 1.37 | 1.79 | Y |
| SNX7 | 1.22 | 0.71 | -0.51 | - | -0.67 | 1.12 | 1.79 | Y |
| CEP250 | 0.34 | -0.38 | -0.72 | - | -0.59 | 1.20 | 1.79 | Y |
| SLC25A15 | 0.39 | -0.21 | -0.60 | - | -0.20 | 1.59 | 1.78 | Y |
| OGG1 | -0.16 | -0.88 | -0.72 | - | -0.76 | 1.02 | 1.78 | Y |
| NEK2 | 2.22 | 1.55 | -0.67 | - | 0.85 | 2.63 | 1.78 | Y |
| TLX3 | -0.57 | -0.68 | -0.12 | - | -0.80 | 0.97 | 1.77 | Y |
| LARP7 | 1.66 | 0.93 | -0.73 | - | -0.52 | 1.23 | 1.75 | Y |
| PFKM | 0.33 | 0.51 | 0.18 | - | -0.27 | 1.46 | 1.73 | Y |
| WNT2 | -0.39 | -0.46 | -0.06 | - | -0.85 | 0.84 | 1.69 | Y |
| POLK | -0.13 | -0.13 | -0.01 | - | -0.65 | 1.03 | 1.68 | Y |
| VAMP5 | -0.79 | -0.53 | 0.25 | - | -0.38 | 1.28 | 1.67 | Y |
| CBX5 | 0.48 | 1.13 | 0.65 | - | -1.07 | 0.59 | 1.66 | Y |
| LSM7 | 0.16 | -0.21 | -0.37 | - | -0.31 | 1.34 | 1.65 | Y |
| GATA3 | 0.27 | -0.31 | -0.58 | - | -0.74 | 0.91 | 1.65 | Y |
| DR1 | -0.05 | -0.83 | -0.78 | - | -0.95 | 0.69 | 1.64 | Y |
| ACAD8 | -0.53 | 0.85 | 1.39 | - | 0.40 | 2.03 | 1.63 | Y |
| NEK11 | 0.68 | 0.15 | -0.53 | - | 0.34 | 1.97 | 1.63 | Y |
| ZNF256 | -0.36 | -0.30 | 0.07 | - | -0.74 | 0.90 | 1.63 | Y |
| FGFR1OP | 1.57 | 1.28 | -0.29 | - | 0.42 | 2.04 | 1.63 | Y |
| CALCOCO1 | 0.71 | 0.16 | -0.55 | - | 0.88 | 2.51 | 1.63 | Y |
| PRDM5 | -0.52 | -0.02 | 0.51 | - | -0.21 | 1.41 | 1.63 | Y |
| RNF40 | 0.07 | -0.14 | -0.20 | - | -0.97 | 0.65 | 1.62 | Y |
| RPP14 | 0.59 | -0.28 | -0.87 | - | -0.41 | 1.19 | 1.60 | Y |
| RECQL4 | 1.26 | 1.31 | 0.05 | - | -0.15 | 1.45 | 1.60 | Y |
| PDGFRA | 0.07 | -0.64 | -0.71 | - | -0.14 | 1.45 | 1.59 | Y |
| TRIM34 | -0.34 | -0.17 | 0.16 | - | 0.55 | 2.13 | 1.58 | Y |
| ZNF655 | 0.54 | -0.10 | -0.65 | - | -0.47 | 1.10 | 1.58 | Y |
| PIWIL1 | 0.45 | 0.99 | 0.54 | - | 0.28 | 1.84 | 1.57 | Y |
| UQCRQ | 0.02 | 0.14 | 0.13 | - | -0.09 | 1.47 | 1.57 | Y |
| BUB3 | 0.66 | 0.13 | -0.53 | - | -0.06 | 1.49 | 1.55 | Y |
| RASSF9 | 0.47 | 0.29 | -0.19 | - | -0.32 | 1.23 | 1.55 | Y |
| NTHL1 | 1.82 | 2.03 | 0.21 | - | -0.70 | 0.84 | 1.55 | Y |
| N4BP2L1 | 1.36 | 0.69 | -0.67 | - | -0.44 | 1.10 | 1.54 | Y |
| NOL4 | 0.27 | -0.27 | -0.53 | - | -0.33 | 1.19 | 1.52 | Y |
| IARS2 | 0.21 | 0.14 | -0.07 | - | -0.85 | 0.66 | 1.51 | Y |
| XPO1 | 1.07 | 2.03 | 0.96 | - | -0.65 | 0.86 | 1.50 | Y |
| RBM17 | 0.32 | -0.23 | -0.54 | - | -0.67 | 0.83 | 1.50 | Y |
| PARP11 | 0.45 | 1.15 | 0.70 | - | -0.69 | 0.81 | 1.49 | Y |
| CHD1L | 1.03 | 0.66 | -0.36 | - | -0.49 | 1.00 | 1.49 | Y |
| PPA2 | 0.52 | 0.47 | -0.05 | - | -0.74 | 0.74 | 1.48 | Y |
| RCC1 | 0.77 | 0.16 | -0.60 | - | 0.06 | 1.51 | 1.44 | Y |
| STAU2 | 0.68 | 2.83 | 2.16 | - | -0.24 | 1.20 | 1.44 | Y |
| TOX4 | 0.39 | 1.24 | 0.85 | - | -0.47 | 0.97 | 1.44 | Y |
| CIRBP | 0.36 | 0.64 | 0.28 | - | 0.59 | 2.03 | 1.43 | Y |
| C1orf74 | 0.70 | 1.60 | 0.90 | - | -1.03 | 0.40 | 1.43 | Y |
| SCYL2 | 0.20 | 1.13 | 0.93 | - | -0.29 | 1.14 | 1.43 | Y |
| CLK3 | 0.50 | 0.32 | -0.18 | - | -1.14 | 0.28 | 1.42 | Y |
| VAX2 | -1.14 | 0.16 | 1.30 | - | -0.59 | 0.83 | 1.42 | Y |
| HNRNPAB | -0.33 | -0.05 | 0.28 | - | 0.61 | 2.03 | 1.42 | Y |
| VAMP2 | 0.70 | 0.70 | 0.00 | - | -0.24 | 1.18 | 1.41 | Y |
| SSX4 | 1.45 | 1.63 | 0.18 | - | -0.07 | 1.33 | 1.40 | Y |
| GTF3C3 | -1.01 | -0.54 | 0.48 | - | 0.13 | 1.53 | 1.40 | Y |
| ALDH4A1 | -0.67 | -0.59 | 0.07 | - | 0.47 | 1.85 | 1.38 | Y |
| PDK2 | 0.77 | 0.66 | -0.11 | - | -0.23 | 1.15 | 1.37 | Y |
| CAMK4 | 0.25 | 0.34 | 0.10 | - | -1.04 | 0.34 | 1.37 | Y |
| ATP5J2 | 0.25 | -0.05 | -0.31 | - | -0.25 | 1.12 | 1.37 | Y |
| EYA1 | 0.37 | -0.33 | -0.70 | - | 0.02 | 1.38 | 1.37 | Y |
| ADRBK2 | 1.07 | 0.86 | -0.21 | - | 0.54 | 1.90 | 1.36 | Y |
| CYB5A | 0.71 | 0.50 | -0.21 | - | 0.68 | 2.03 | 1.35 | Y |
| TAB1 | 0.04 | 0.35 | 0.31 | - | -0.74 | 0.61 | 1.35 | Y |
| ALB | 1.54 | 0.94 | -0.60 | - | -0.01 | 1.33 | 1.34 | Y |
| MRPL30 | -0.93 | -0.35 | 0.58 | - | -0.05 | 1.28 | 1.33 | Y |
| TPPP | -0.15 | -0.54 | -0.40 | - | -0.40 | 0.92 | 1.32 | Y |
| ZNF550 | 0.50 | 0.22 | -0.28 | - | 0.07 | 1.38 | 1.31 | Y |
| AIPL1 | 0.15 | 0.90 | 0.76 | - | -0.51 | 0.80 | 1.31 | Y |
| RPA3 | -0.64 | -1.01 | -0.38 | - | -0.51 | 0.79 | 1.31 | Y |
| TMPO | 0.56 | -0.16 | -0.72 | - | -1.15 | 0.16 | 1.31 | Y |
| SAP18 | -0.48 | -0.56 | -0.08 | - | 0.25 | 1.55 | 1.30 | Y |
| TRIM24 | -0.07 | -0.30 | -0.23 | - | 1.37 | 2.66 | 1.29 | Y |
| ZFP36 | 1.19 | 0.37 | -0.82 | - | -0.66 | 0.62 | 1.28 | Y |
| OTUD7B | 0.36 | 0.78 | 0.42 | - | -0.60 | 0.68 | 1.28 | Y |
| POLR3F | 0.34 | 0.62 | 0.28 | - | -1.38 | -0.11 | 1.28 | Y |
| KIAA0020 | 1.41 | 1.18 | -0.23 | - | 0.05 | 1.33 | 1.27 | Y |
| EIF5A | 1.10 | 0.66 | -0.44 | - | -0.30 | 0.97 | 1.27 | Y |
| ATP5G3 | 0.61 | 0.41 | -0.20 | - | 0.04 | 1.31 | 1.27 | Y |
| IP6K2 | 0.70 | -0.13 | -0.83 | - | -0.33 | 0.93 | 1.27 | Y |
| MAGEB2 | 0.81 | 0.46 | -0.34 | - | -0.19 | 1.08 | 1.27 | Y |
| PSME1 | 0.72 | 0.00 | -0.73 | - | -0.10 | 1.16 | 1.26 | Y |
| NEUROD6 | 1.31 | 0.71 | -0.60 | - | 0.87 | 2.13 | 1.26 | Y |
| ZNF718 | 1.50 | 0.85 | -0.65 | - | -0.15 | 1.11 | 1.25 | Y |
| SETDB1 | 0.19 | 1.08 | 0.89 | - | -0.46 | 0.79 | 1.25 | Y |
| NASP | 0.32 | 0.39 | 0.07 | - | -0.57 | 0.68 | 1.25 | Y |
| HBS1L | 0.83 | 0.22 | -0.61 | - | -0.86 | 0.38 | 1.24 | Y |
| ZNF28 | -0.43 | -0.01 | 0.41 | - | -0.69 | 0.55 | 1.24 | Y |
| NCAPH2 | -0.06 | -0.63 | -0.57 | - | -0.38 | 0.86 | 1.24 | Y |
| TSC22D4 | 0.09 | -0.30 | -0.40 | - | 0.00 | 1.23 | 1.23 | Y |
| UCP3 | 0.36 | 0.17 | -0.19 | - | -0.32 | 0.92 | 1.23 | Y |
| NR1I2 | 0.56 | -0.24 | -0.80 | - | -0.27 | 0.96 | 1.23 | Y |
| DNASE1L1 | 0.03 | 1.38 | 1.36 | - | -0.09 | 1.14 | 1.23 | Y |
| RBMY1A1 | 0.02 | -0.13 | -0.15 | - | -0.92 | 0.30 | 1.23 | Y |
| HDGF | 1.39 | 1.31 | -0.08 | - | 0.20 | 1.42 | 1.23 | Y |
| TAZ | 1.66 | 0.80 | -0.86 | - | -1.10 | 0.13 | 1.22 | Y |
| NCOA2 | 0.44 | 2.10 | 1.67 | - | -1.41 | -0.19 | 1.22 | Y |
| POLR1A | 0.89 | 1.34 | 0.45 | - | -0.64 | 0.58 | 1.22 | Y |
| ZNF280B | 0.12 | -0.71 | -0.84 | - | -0.82 | 0.40 | 1.22 | Y |
| FABP4 | 1.16 | 0.37 | -0.79 | - | -0.87 | 0.34 | 1.21 | Y |
| POGZ | -0.84 | 0.17 | 1.01 | - | -0.91 | 0.29 | 1.21 | Y |
| ZNF738 | 0.70 | -0.06 | -0.76 | - | 0.28 | 1.48 | 1.20 | Y |
| BTBD12 | 0.90 | 1.80 | 0.90 | - | 0.12 | 1.32 | 1.19 | Y |
| RPS6KB1 | -0.32 | 0.66 | 0.97 | - | -0.29 | 0.90 | 1.19 | Y |
| TOMM40 | -0.58 | 0.67 | 1.25 | - | -0.47 | 0.72 | 1.19 | Y |
| PAIP1 | 1.00 | 0.20 | -0.80 | - | -0.38 | 0.81 | 1.19 | Y |
| ERCC3 | 0.40 | 0.53 | 0.13 | - | -0.57 | 0.62 | 1.19 | Y |
| NUAK1 | 0.46 | -0.11 | -0.57 | - | -0.25 | 0.93 | 1.18 | Y |
| EXOSC3 | 0.86 | 1.53 | 0.67 | - | -0.31 | 0.87 | 1.18 | Y |
| ZNF295 | 1.36 | 0.81 | -0.55 | - | 0.31 | 1.48 | 1.18 | Y |
| C6orf115 | 1.87 | 1.12 | -0.74 | - | 0.00 | 1.17 | 1.17 | Y |
| NBPF3 | -0.19 | 0.12 | 0.31 | - | 0.52 | 1.69 | 1.17 | Y |
| HEYL | 0.05 | -0.76 | -0.81 | - | -0.38 | 0.78 | 1.16 | Y |
| SUPT5H | -0.50 | -0.83 | -0.33 | - | -0.46 | 0.70 | 1.16 | Y |
| SRXN1 | 1.24 | 0.70 | -0.54 | - | 0.44 | 1.60 | 1.16 | Y |
| STK24 | 0.92 | 0.68 | -0.24 | - | -0.58 | 0.58 | 1.16 | Y |
| SURF1 | 0.34 | -0.03 | -0.37 | - | -0.63 | 0.53 | 1.16 | Y |
| ALPK1 | 2.50 | 4.08 | 1.58 | - | 4.43 | 5.59 | 1.16 | Y |
| FLJ14299 | 0.05 | -0.26 | -0.31 | - | -0.71 | 0.44 | 1.15 | Y |
| TGFBR2 | 1.86 | 1.72 | -0.14 | - | -0.33 | 0.82 | 1.14 | Y |
| TEAD4 | -0.83 | -0.17 | 0.66 | - | -0.99 | 0.15 | 1.14 | Y |
| ZNF649 | 0.56 | 0.28 | -0.28 | - | -1.40 | -0.26 | 1.14 | Y |
| KCNIP3 | 0.09 | -0.53 | -0.62 | - | -1.21 | -0.08 | 1.14 | Y |
| HIP14 | 1.01 | 0.27 | -0.74 | - | -0.35 | 0.79 | 1.14 | Y |
| RPP25 | -0.64 | 0.09 | 0.73 | - | -0.80 | 0.34 | 1.14 | Y |
| XRCC3 | 0.88 | 2.05 | 1.17 | - | -0.61 | 0.52 | 1.12 | Y |
| HZF12 | -0.30 | 0.96 | 1.25 | - | -0.53 | 0.59 | 1.12 | Y |
| CSRP1 | -0.07 | 0.52 | 0.59 | - | -0.53 | 0.59 | 1.12 | Y |
| CNBP | 0.01 | -0.41 | -0.42 | - | -0.35 | 0.77 | 1.12 | Y |
| ZNF584 | -0.77 | -1.01 | -0.24 | - | -1.71 | -0.59 | 1.12 | Y |
| ACADM | 1.79 | 0.99 | -0.79 | - | -0.59 | 0.52 | 1.11 | Y |
| SMYD2 | -0.88 | -0.82 | 0.06 | - | -0.43 | 0.66 | 1.10 | Y |
| ALDH3B1 | -0.36 | 1.87 | 2.23 | - | -0.47 | 0.63 | 1.10 | Y |
| EXOSC5 | 0.65 | 0.73 | 0.08 | - | -0.26 | 0.82 | 1.08 | Y |
| PDS5B | -0.45 | 0.06 | 0.51 | - | 0.06 | 1.14 | 1.08 | Y |
| ALKBH1 | 0.86 | 0.85 | -0.02 | - | -0.93 | 0.14 | 1.07 | Y |
| HMGCS2 | 0.78 | -0.02 | -0.80 | - | 0.40 | 1.47 | 1.07 | Y |
| DIDO1 | 0.51 | 0.45 | -0.06 | - | -0.36 | 0.72 | 1.07 | Y |
| RNF14 | 0.32 | -0.15 | -0.47 | - | -0.98 | 0.09 | 1.07 | Y |
| AWP1 | 0.83 | 1.28 | 0.44 | - | -0.21 | 0.85 | 1.06 | Y |
| LYZ | 0.07 | 0.32 | 0.25 | - | -0.51 | 0.55 | 1.06 | Y |
| CLK1 | 3.42 | 2.95 | -0.48 | - | 0.03 | 1.09 | 1.06 | Y |
| ZNF8 | 0.84 | 1.95 | 1.11 | - | 0.50 | 1.56 | 1.06 | Y |
| NDUFA8 | 0.73 | 0.49 | -0.23 | - | -0.39 | 0.65 | 1.05 | Y |
| SPEG | 1.02 | 0.37 | -0.65 | - | 0.11 | 1.15 | 1.05 | Y |
| SNRK | 0.45 | 0.10 | -0.35 | - | -0.62 | 0.42 | 1.04 | Y |
| ADCK2 | -0.62 | -0.38 | 0.25 | - | -0.53 | 0.51 | 1.04 | Y |
| AURKC | 0.01 | -0.62 | -0.64 | - | -0.16 | 0.87 | 1.03 | Y |
| PRKCZ | 0.38 | 0.12 | -0.26 | - | -0.23 | 0.80 | 1.03 | Y |
| MYOG | -0.27 | 0.16 | 0.43 | - | 0.47 | 1.50 | 1.02 | Y |
| PPP2R1A | 0.31 | 0.04 | -0.27 | - | -0.62 | 0.40 | 1.02 | Y |
| ZCCHC14 | 0.40 | 0.42 | 0.02 | - | 0.11 | 1.13 | 1.02 | Y |
| SUCLG1 | 0.64 | 1.46 | 0.81 | - | 0.39 | 1.41 | 1.02 | Y |
| ZBED3 | 0.13 | 0.95 | 0.82 | - | -0.76 | 0.25 | 1.02 | Y |
| ZKSCAN3 | -0.36 | -0.59 | -0.23 | - | -0.51 | 0.51 | 1.02 | Y |
| RBM38 | 0.13 | 0.17 | 0.04 | - | -0.75 | 0.26 | 1.01 | Y |
| TIMM9 | -0.07 | -0.23 | -0.16 | - | -0.78 | 0.23 | 1.01 | Y |
| PTGER3 | -0.33 | 0.52 | 0.85 | - | -0.55 | 0.46 | 1.01 | Y |
| ARNTL2 | 0.13 | 0.16 | 0.04 | - | -0.70 | 0.31 | 1.01 | Y |
| UTP18 | 0.62 | 0.60 | -0.03 | - | -1.00 | 0.01 | 1.00 | Y |
| ZNF581 | 0.35 | 0.72 | 0.37 | - | 0.12 | 1.12 | 1.00 | Y |
| CTSD | 0.04 | -0.22 | -0.26 | - | -0.80 | 0.20 | 1.00 | Y |
| ASF1A | 0.16 | 0.05 | -0.12 | - | -0.06 | 0.94 | 1.00 | Y |
| SCO2 | -0.16 | -0.03 | 0.13 | - | 0.77 | 1.76 | 1.00 | Y |
| ZNF688 | 0.51 | 0.85 | 0.34 | - | 0.30 | 1.30 | 0.99 | Y |
| XPO5 | 0.02 | 0.37 | 0.36 | - | -0.15 | 0.84 | 0.99 | Y |
| ASCL2 | -0.23 | 1.24 | 1.47 | - | -0.22 | 0.77 | 0.99 | Y |
| LARP1B | -0.30 | 0.55 | 0.85 | - | -0.49 | 0.49 | 0.98 | Y |
| MRPL41 | 1.64 | 1.01 | -0.63 | - | -0.85 | 0.14 | 0.98 | Y |
| MTHFD2L | 0.22 | 1.05 | 0.83 | - | -1.27 | -0.29 | 0.98 | Y |
| TBX2 | 0.31 | 0.83 | 0.52 | - | -0.49 | 0.49 | 0.98 | Y |
| HSF2 | 1.17 | 0.32 | -0.85 | - | 0.39 | 1.36 | 0.97 | Y |
| AGAP2 | 0.67 | -0.17 | -0.84 | - | 0.28 | 1.25 | 0.96 | Y |
| CD86 | 0.64 | -0.08 | -0.72 | - | -0.17 | 0.79 | 0.96 | Y |
| DDX42 | 1.35 | 0.56 | -0.79 | - | -1.06 | -0.10 | 0.96 | Y |
| TIMM17A | -0.30 | -0.49 | -0.19 | - | -1.22 | -0.27 | 0.96 | Y |
| UQCRC1 | 0.21 | -0.53 | -0.74 | - | -0.55 | 0.41 | 0.95 | Y |
| GRRP1 | 0.34 | 0.65 | 0.30 | - | -0.72 | 0.23 | 0.95 | Y |
| MRPL22 | -0.74 | 0.09 | 0.83 | - | -0.03 | 0.91 | 0.94 | Y |
| MAFK | 0.95 | 1.05 | 0.10 | - | -0.81 | 0.13 | 0.94 | Y |
| TRMT11 | -0.93 | 0.84 | 1.77 | - | 0.55 | 1.49 | 0.94 | Y |
| POLD1 | 0.64 | 0.03 | -0.61 | - | -0.04 | 0.89 | 0.93 | Y |
| HSPA9 | -0.40 | 0.33 | 0.72 | - | -0.99 | -0.06 | 0.93 | Y |
| ZNF607 | -0.02 | 0.44 | 0.46 | - | -0.19 | 0.74 | 0.93 | Y |
| HMGN3 | 0.02 | -0.80 | -0.82 | - | -0.61 | 0.31 | 0.92 | Y |
| SHOX | 0.69 | 0.05 | -0.64 | - | -0.93 | -0.01 | 0.92 | Y |
| CEBPB | 0.21 | 0.21 | -0.01 | - | 0.19 | 1.11 | 0.92 | Y |
| PPP2R3B | 0.64 | 0.66 | 0.02 | - | -0.34 | 0.57 | 0.91 | Y |
| SSX2 | 0.68 | -0.04 | -0.72 | - | 0.50 | 1.41 | 0.91 | Y |
| FMR1 | -0.08 | -0.70 | -0.63 | - | -0.64 | 0.27 | 0.91 | Y |
| ZC3H3 | -0.45 | 1.64 | 2.09 | - | -0.72 | 0.19 | 0.91 | Y |
| NFIC | 0.59 | 1.22 | 0.63 | - | -1.12 | -0.21 | 0.91 | Y |
| POLD4 | 1.13 | 1.74 | 0.61 | - | -0.34 | 0.56 | 0.90 | Y |
| ZNF625 | -0.34 | -0.87 | -0.53 | - | -0.16 | 0.74 | 0.90 | Y |
| GNB4 | 0.24 | 0.00 | -0.23 | - | -0.36 | 0.54 | 0.90 | Y |
| PPDPF | -0.03 | 0.70 | 0.73 | - | -0.65 | 0.24 | 0.89 | Y |
| NR1D1 | 1.22 | 0.68 | -0.53 | - | 1.97 | 2.86 | 0.89 | Y |
| ZNF167 | 0.16 | 0.95 | 0.80 | - | -0.54 | 0.35 | 0.89 | Y |
| MRPL4 | 0.35 | -0.29 | -0.64 | - | -0.17 | 0.72 | 0.89 | Y |
| CEBPZ | 0.01 | -0.33 | -0.35 | - | -1.27 | -0.39 | 0.88 | Y |
| RFX4 | 0.25 | 0.10 | -0.15 | - | 0.16 | 1.04 | 0.88 | Y |
| ABAT | 0.43 | -0.01 | -0.44 | - | 1.03 | 1.90 | 0.87 | Y |
| SLC25A27 | 0.29 | 0.56 | 0.27 | - | -0.20 | 0.66 | 0.87 | Y |
| HAND1 | 1.24 | 0.48 | -0.76 | - | -0.38 | 0.48 | 0.86 | Y |
| CHURC1 | 0.56 | -0.13 | -0.68 | - | 0.25 | 1.10 | 0.85 | Y |
| SP140 | -0.02 | -0.80 | -0.77 | - | -0.53 | 0.32 | 0.85 | Y |
| PRKAA2 | -0.45 | -0.31 | 0.14 | - | -0.43 | 0.42 | 0.85 | Y |
| TGFBI | 1.15 | 0.69 | -0.45 | - | 0.10 | 0.94 | 0.84 | Y |
| MDM4 | 2.18 | 1.45 | -0.73 | - | 0.91 | 1.75 | 0.84 | Y |
| TAF9B | 0.89 | 1.58 | 0.70 | - | 0.26 | 1.09 | 0.84 | Y |
| ZNF765 | 1.00 | 0.61 | -0.39 | - | -0.96 | -0.13 | 0.83 | Y |
| LYPLAL1 | 0.20 | 0.82 | 0.62 | - | -0.97 | -0.14 | 0.83 | Y |
| STK11 | 0.97 | 0.35 | -0.62 | - | 0.69 | 1.52 | 0.83 | Y |
| IRF4 | 0.60 | 0.62 | 0.01 | - | -1.29 | -0.46 | 0.83 | Y |
| ZFP91 | 0.28 | -0.18 | -0.47 | - | -0.24 | 0.59 | 0.83 | Y |
| ACN9 | 0.23 | 0.08 | -0.15 | - | -1.02 | -0.19 | 0.82 | Y |
| PNMA2 | 1.11 | 0.61 | -0.50 | - | -0.74 | 0.09 | 0.82 | Y |
| ZFP90 | 0.63 | 0.33 | -0.29 | - | -0.41 | 0.40 | 0.81 | Y |
| MPP1 | -0.04 | -0.64 | -0.59 | - | 0.54 | 1.34 | 0.81 | Y |
| ARIH2 | -0.27 | 1.31 | 1.58 | - | -0.13 | 0.68 | 0.81 | Y |
| RFXANK | -0.04 | 0.10 | 0.14 | - | -0.73 | 0.08 | 0.80 | Y |
| NFATC4 | -0.01 | 0.20 | 0.20 | - | -0.67 | 0.13 | 0.80 | Y |
| CSNK1D | 0.44 | 1.30 | 0.86 | - | 0.02 | 0.82 | 0.80 | Y |
| MAPKAPK5 | 0.21 | 0.74 | 0.53 | - | 0.08 | 0.88 | 0.80 | Y |
| CYorf15A | -0.71 | -0.05 | 0.66 | - | -0.27 | 0.52 | 0.80 | Y |
| TIMM10 | 1.28 | 1.04 | -0.23 | - | -1.21 | -0.41 | 0.80 | Y |
| CBFA2T3 | 2.73 | 2.43 | -0.30 | - | 1.28 | 2.07 | 0.79 | Y |
| BRF1 | 1.11 | 0.90 | -0.20 | - | -0.63 | 0.16 | 0.79 | Y |
| ZBTB9 | 0.37 | 0.09 | -0.28 | - | 0.35 | 1.14 | 0.78 | Y |
| PMPCA | -0.92 | -1.02 | -0.10 | - | -0.53 | 0.25 | 0.78 | Y |
| ALDH2 | -0.69 | -0.88 | -0.19 | - | -1.18 | -0.40 | 0.78 | Y |
| WWTR1 | 0.61 | 0.40 | -0.22 | - | -0.23 | 0.55 | 0.78 | Y |
| PDHA1 | 0.64 | 0.03 | -0.60 | - | 0.07 | 0.85 | 0.78 | Y |
| NFYC | 0.28 | 0.46 | 0.18 | - | 1.06 | 1.83 | 0.78 | Y |
| RNF122 | 0.10 | -0.10 | -0.19 | - | -0.23 | 0.54 | 0.77 | Y |
| MRPL9 | 1.06 | 0.87 | -0.20 | - | -0.47 | 0.30 | 0.77 | Y |
| IRAK3 | 0.40 | 0.34 | -0.06 | - | -0.58 | 0.19 | 0.77 | Y |
| SNRPN | -0.14 | -0.19 | -0.05 | - | 1.77 | 2.53 | 0.77 | Y |
| CPEB4 | -0.63 | 0.32 | 0.96 | - | 0.40 | 1.17 | 0.77 | Y |
| CSDC2 | 1.03 | 0.68 | -0.36 | - | -0.44 | 0.32 | 0.77 | Y |
| NAP1L5 | -0.44 | 0.10 | 0.53 | - | -0.50 | 0.26 | 0.76 | Y |
| NIPSNAP3B | 0.69 | 0.71 | 0.02 | - | 0.02 | 0.78 | 0.76 | Y |
| HIBADH | 0.77 | 1.24 | 0.48 | - | 0.29 | 1.04 | 0.76 | Y |
| ZFAND3 | 0.34 | 0.02 | -0.31 | - | -0.85 | -0.10 | 0.75 | Y |
| AIMP1 | 0.20 | 0.96 | 0.76 | - | -0.66 | 0.09 | 0.75 | Y |
| ATM | 0.53 | -0.20 | -0.73 | - | -0.09 | 0.65 | 0.74 | Y |
| ZNF268 | 0.22 | -0.38 | -0.60 | - | -0.70 | 0.04 | 0.74 | Y |
| TIAL1 | 1.69 | 3.20 | 1.50 | - | 0.72 | 1.45 | 0.74 | Y |
| HLCS | 0.77 | 0.82 | 0.05 | - | 0.18 | 0.91 | 0.73 | Y |
| BMPR1A | -0.29 | 0.14 | 0.42 | - | -0.34 | 0.39 | 0.73 | Y |
| PMF1 | 0.31 | -0.20 | -0.51 | - | -0.28 | 0.45 | 0.73 | Y |
| EBF4 | -0.48 | 0.01 | 0.49 | - | -0.51 | 0.22 | 0.72 | Y |
| ESRRA | 1.38 | 0.69 | -0.69 | - | 0.04 | 0.77 | 0.72 | Y |
| DNAJC19 | 0.80 | 0.28 | -0.52 | - | 0.58 | 1.30 | 0.72 | Y |
| CPLX2 | -0.85 | -0.37 | 0.48 | - | -0.40 | 0.32 | 0.72 | Y |
| FOXP2 | 0.77 | 0.35 | -0.42 | - | 0.14 | 0.85 | 0.72 | Y |
| REXO2 | 1.01 | 0.68 | -0.33 | - | -0.42 | 0.29 | 0.71 | Y |
| NDUFV2 | -0.06 | 0.53 | 0.59 | - | -0.33 | 0.37 | 0.70 | Y |
| CSDA | -0.18 | -0.53 | -0.34 | - | -0.34 | 0.36 | 0.70 | Y |
| ATP5A1 | 0.29 | 0.75 | 0.46 | - | -0.64 | 0.06 | 0.70 | Y |
| BACH1 | -0.16 | 0.33 | 0.50 | - | -0.62 | 0.07 | 0.70 | Y |
| HMBOX1 | -0.64 | 0.91 | 1.55 | - | -0.79 | -0.09 | 0.70 | Y |
| ITK | 0.92 | 0.42 | -0.50 | - | 0.13 | 0.82 | 0.69 | Y |
| PPP3CB | 1.36 | 1.70 | 0.34 | - | 0.17 | 0.86 | 0.69 | Y |
| NUCB1 | 1.67 | 0.91 | -0.75 | - | -0.66 | 0.02 | 0.69 | Y |
| ATRX | -0.01 | -0.36 | -0.35 | - | -0.40 | 0.29 | 0.68 | Y |
| AIMP2 | 0.88 | 0.44 | -0.44 | - | 0.44 | 1.12 | 0.68 | Y |
| ZDHHC13 | -0.10 | 0.02 | 0.12 | - | -1.18 | -0.50 | 0.68 | Y |
| NRP1 | 0.59 | 0.27 | -0.32 | - | -0.75 | -0.07 | 0.68 | Y |
| HBG2 | -0.51 | -0.74 | -0.23 | - | -0.45 | 0.23 | 0.68 | Y |
| EIF4G3 | -0.56 | -0.17 | 0.39 | - | 0.08 | 0.75 | 0.67 | Y |
| PAX8 | 0.21 | -0.40 | -0.61 | - | -0.60 | 0.07 | 0.67 | Y |
| NEK7 | 0.97 | 0.53 | -0.43 | - | -0.33 | 0.34 | 0.67 | Y |
| ZNF34 | 0.42 | 0.25 | -0.18 | - | -0.03 | 0.64 | 0.67 | Y |
| PICK1 | 0.72 | 1.38 | 0.66 | - | -0.26 | 0.40 | 0.67 | Y |
| PCBP2 | -0.88 | -0.95 | -0.07 | - | -0.78 | -0.11 | 0.66 | Y |
| ZNF683 | 0.65 | 1.19 | 0.54 | - | -1.33 | -0.67 | 0.66 | Y |
| SLC25A22 | 0.15 | 1.09 | 0.94 | - | -0.34 | 0.32 | 0.66 | Y |
| NEK8 | 0.57 | -0.29 | -0.86 | - | -1.23 | -0.57 | 0.66 | Y |
| LARP6 | 1.68 | 0.82 | -0.86 | - | 0.35 | 1.00 | 0.66 | Y |
| TEAD3 | 0.28 | 0.80 | 0.52 | - | -0.20 | 0.46 | 0.66 | Y |
| SNRPB | 0.39 | 0.19 | -0.20 | - | -0.56 | 0.09 | 0.65 | Y |
| RBM39 | 0.72 | 0.79 | 0.07 | - | -0.62 | 0.03 | 0.65 | Y |
| TAF13 | 0.15 | -0.11 | -0.26 | - | -0.69 | -0.04 | 0.65 | Y |
| PPIH | 1.87 | 1.03 | -0.83 | - | -0.84 | -0.19 | 0.65 | Y |
| CDK18 | 0.42 | -0.43 | -0.85 | - | 0.56 | 1.20 | 0.64 | Y |
| OTX1 | 0.11 | -0.39 | -0.51 | - | -0.56 | 0.09 | 0.64 | Y |
| MED20 | 0.57 | 0.28 | -0.29 | - | -0.26 | 0.38 | 0.64 | Y |
| ZNF75A | 0.43 | 0.94 | 0.51 | - | -0.51 | 0.13 | 0.64 | Y |
| C14orf2 | 0.28 | 0.64 | 0.36 | - | -0.85 | -0.21 | 0.63 | Y |
| ERCC8 | 0.51 | -0.22 | -0.73 | - | 0.47 | 1.10 | 0.63 | Y |
| PRDX3 | -1.02 | -0.77 | 0.25 | - | -0.92 | -0.29 | 0.62 | Y |
| HMGB1 | 1.45 | 0.59 | -0.87 | - | -1.00 | -0.39 | 0.62 | Y |
| C1orf183 | 0.38 | -0.27 | -0.65 | - | 0.09 | 0.71 | 0.62 | Y |
| ZNF81 | -0.09 | -0.20 | -0.11 | - | 0.03 | 0.65 | 0.62 | Y |
| HDAC3 | 0.64 | -0.14 | -0.78 | - | 0.01 | 0.63 | 0.62 | Y |
| PCCB | -0.46 | -0.16 | 0.29 | - | -0.64 | -0.03 | 0.61 | Y |
| NR2E1 | 0.44 | 0.80 | 0.36 | - | -0.80 | -0.19 | 0.61 | Y |
| MYCL1 | 0.61 | -0.02 | -0.63 | - | -0.70 | -0.09 | 0.61 | Y |
| IRF9 | 0.06 | 0.57 | 0.51 | - | -0.35 | 0.26 | 0.61 | Y |
| CDK15 | -1.20 | 0.56 | 1.76 | - | -0.62 | -0.01 | 0.61 | Y |
| NT5C3 | -0.11 | -0.75 | -0.64 | - | -0.24 | 0.36 | 0.60 | Y |
| ZNF808 | 0.16 | -0.04 | -0.21 | - | -0.96 | -0.36 | 0.60 | Y |
| GTF2F2 | -0.08 | -0.21 | -0.13 | - | 0.57 | 1.18 | 0.60 | Y |
| PRMT6 | 0.29 | -0.28 | -0.57 | - | -0.43 | 0.17 | 0.60 | Y |
| TNFAIP3 | -0.39 | 0.64 | 1.03 | - | -0.49 | 0.10 | 0.60 | Y |
| ACTL9 | -0.05 | 0.15 | 0.20 | - | -0.62 | -0.02 | 0.60 | Y |
| TARDBP | 0.02 | -0.07 | -0.08 | - | -0.41 | 0.19 | 0.60 | Y |
| RDH13 | -0.05 | -0.55 | -0.51 | - | -0.61 | -0.02 | 0.60 | Y |
| G6PD | 0.23 | -0.58 | -0.81 | - | -0.57 | 0.03 | 0.59 | Y |
| HIPK4 | 0.22 | 0.81 | 0.59 | - | -0.97 | -0.37 | 0.59 | Y |
| ZSCAN20 | -0.48 | -0.21 | 0.27 | - | -0.05 | 0.54 | 0.59 | Y |
| PGBD1 | 0.06 | -0.20 | -0.26 | - | 0.42 | 1.01 | 0.59 | Y |
| GATA4 | -0.29 | -0.80 | -0.51 | - | -0.69 | -0.10 | 0.59 | Y |
| ARFGAP1 | 1.11 | 0.71 | -0.40 | - | 0.62 | 1.21 | 0.59 | Y |
| STRADB | 0.38 | -0.14 | -0.52 | - | -0.24 | 0.35 | 0.59 | Y |
| PTRF | 0.93 | 1.81 | 0.88 | - | -0.34 | 0.24 | 0.58 | Y |
| COX4I2 | 1.08 | 0.22 | -0.86 | - | -0.45 | 0.13 | 0.58 | Y |
| EHD4 | 1.69 | 0.95 | -0.74 | - | -0.60 | -0.02 | 0.58 | Y |
| FLJ20323 | 1.13 | 0.98 | -0.15 | - | -0.71 | -0.13 | 0.57 | Y |
| HCCS | 0.65 | -0.07 | -0.72 | - | 0.22 | 0.79 | 0.57 | Y |
| ACVRL1 | 0.71 | 0.28 | -0.43 | - | -0.88 | -0.31 | 0.57 | Y |
| FLJ23233 | -0.04 | -0.10 | -0.06 | - | -0.46 | 0.11 | 0.57 | Y |
| ASF1B | 0.32 | 0.10 | -0.23 | - | -0.94 | -0.37 | 0.57 | Y |
| RNF144A | 0.23 | -0.56 | -0.79 | - | -0.67 | -0.10 | 0.57 | Y |
| GOLGA5 | -0.49 | -0.35 | 0.13 | - | -0.23 | 0.33 | 0.57 | Y |
| GAR1 | -0.60 | -0.73 | -0.13 | - | -0.60 | -0.03 | 0.57 | Y |
| MTHFD2 | 1.00 | 1.44 | 0.44 | - | -0.61 | -0.05 | 0.56 | Y |
| EDN1 | -0.16 | -0.64 | -0.48 | - | -0.95 | -0.39 | 0.56 | Y |
| HNF4A | -0.25 | -0.83 | -0.58 | - | -0.35 | 0.21 | 0.56 | Y |
| PCBP4 | 0.20 | 0.71 | 0.50 | - | 0.83 | 1.39 | 0.56 | Y |
| ZNF274 | 0.20 | -0.03 | -0.23 | - | 0.18 | 0.74 | 0.56 | Y |
| CSRP3 | 0.14 | 0.79 | 0.66 | - | -0.67 | -0.12 | 0.56 | Y |
| POLA2 | 1.27 | 0.46 | -0.81 | - | -0.13 | 0.42 | 0.56 | Y |
| TSSK3 | 1.43 | 0.82 | -0.61 | - | 0.36 | 0.92 | 0.56 | Y |
| MUS81 | -0.20 | -0.05 | 0.16 | - | -0.68 | -0.13 | 0.55 | Y |
| KLF11 | -0.04 | 1.41 | 1.44 | - | -0.09 | 0.47 | 0.55 | Y |
| PEX3 | 0.68 | 0.12 | -0.56 | - | -0.30 | 0.25 | 0.55 | Y |
| LBX2 | -0.32 | -0.44 | -0.11 | - | -0.32 | 0.22 | 0.55 | Y |
| CBL | 1.23 | 0.60 | -0.62 | - | 0.85 | 1.40 | 0.55 | Y |
| CALCOCO2 | 1.90 | 1.43 | -0.47 | - | 0.38 | 0.92 | 0.54 | Y |
| ZNF160 | 0.08 | 0.80 | 0.72 | - | -0.59 | -0.05 | 0.54 | Y |
| VAMP8 | 0.77 | 0.43 | -0.34 | - | -0.19 | 0.35 | 0.54 | Y |
| ZNF281 | -0.43 | 0.59 | 1.02 | - | 0.08 | 0.62 | 0.54 | Y |
| EPHA7 | 0.49 | -0.26 | -0.75 | - | -0.79 | -0.26 | 0.53 | Y |
| NDUFA5 | 0.81 | 0.43 | -0.38 | - | 0.51 | 1.04 | 0.53 | Y |
| GOT2 | 0.16 | -0.21 | -0.37 | - | -0.47 | 0.05 | 0.52 | Y |
| MYL9 | 0.84 | 0.22 | -0.62 | - | 0.57 | 1.08 | 0.51 | Y |
| SLC25A20 | -0.91 | 1.07 | 1.99 | - | -0.32 | 0.19 | 0.50 | Y |
| FEV | 0.37 | 1.39 | 1.02 | - | -0.44 | 0.07 | 0.50 | Y |
| RBM3 | -0.13 | -0.05 | 0.08 | - | -0.94 | -0.44 | 0.50 | Y |
| ZCCHC2 | 1.61 | 0.77 | -0.84 | - | 0.12 | 0.62 | 0.50 | Y |
| EEF1A2 | 0.74 | 0.60 | -0.15 | - | -0.59 | -0.09 | 0.50 | Y |
| CAMK2B | 1.04 | 0.41 | -0.63 | - | -0.55 | -0.05 | 0.50 | Y |
| TXNL2 | 1.06 | 1.91 | 0.85 | - | 0.30 | 0.80 | 0.50 | Y |
| CSK | -0.58 | 0.06 | 0.64 | - | -0.67 | -0.17 | 0.50 | Y |
| BTBD3 | 1.10 | 1.20 | 0.11 | - | -0.87 | -0.37 | 0.50 | Y |
| LAS1L | 0.01 | -0.16 | -0.17 | - | -0.75 | -0.25 | 0.50 | Y |
| SLC30A9 | -0.24 | -0.43 | -0.18 | - | -0.28 | 0.22 | 0.50 | Y |
| APOA1 | -0.68 | 0.14 | 0.82 | - | 0.50 | 0.99 | 0.49 | Y |
| SALL2 | 0.17 | 0.08 | -0.09 | - | -0.51 | -0.01 | 0.49 | Y |
| SNAPC3 | 0.65 | -0.11 | -0.76 | - | -0.94 | -0.45 | 0.49 | Y |
| SSX9 | 0.36 | 0.06 | -0.30 | - | -0.78 | -0.29 | 0.49 | Y |
| CAMK1 | 1.15 | 1.11 | -0.04 | - | -1.29 | -0.80 | 0.49 | Y |
| ACAD9 | 0.45 | -0.08 | -0.52 | - | -0.80 | -0.32 | 0.49 | Y |
| GINS2 | 0.74 | -0.07 | -0.82 | - | -1.06 | -0.57 | 0.48 | Y |
| ZNF576 | 1.54 | 1.55 | 0.01 | - | 0.77 | 1.25 | 0.48 | Y |
| SLC9A6 | -0.68 | -1.53 | -0.85 | - | 0.04 | 0.52 | 0.48 | Y |
| ETFB | 0.30 | -0.55 | -0.86 | - | 0.17 | 0.65 | 0.48 | Y |
| JAZF1 | 0.37 | 0.27 | -0.10 | - | 0.90 | 1.38 | 0.48 | Y |
| UBE2C | 0.51 | 0.59 | 0.08 | - | -0.45 | 0.03 | 0.48 | Y |
| GDUOK | 0.48 | 1.22 | 0.75 | - | -0.78 | -0.30 | 0.48 | Y |
| PCK2 | 0.66 | 0.40 | -0.25 | - | -0.48 | 0.00 | 0.48 | Y |
| DUSP26 | 1.42 | 0.57 | -0.85 | - | 0.85 | 1.33 | 0.48 | Y |
| RELA | 0.10 | -0.28 | -0.38 | - | -0.25 | 0.23 | 0.48 | Y |
| NIP7 | -0.93 | 0.76 | 1.69 | - | -0.87 | -0.39 | 0.47 | Y |
| NKRF | -0.47 | 1.61 | 2.08 | - | -0.47 | 0.00 | 0.47 | Y |
| SLC18A1 | 0.03 | -0.22 | -0.26 | - | -0.71 | -0.24 | 0.47 | Y |
| OLIG1 | -0.16 | -0.08 | 0.08 | - | -1.28 | -0.82 | 0.47 | Y |
| BMP2K | 0.34 | -0.11 | -0.46 | - | 0.04 | 0.51 | 0.47 | Y |
| MSI2 | 0.24 | -0.61 | -0.85 | - | -0.25 | 0.21 | 0.47 | Y |
| T | -0.75 | 0.64 | 1.39 | - | -0.25 | 0.21 | 0.46 | Y |
| PGM1 | -0.82 | 0.26 | 1.08 | - | 0.28 | 0.74 | 0.46 | Y |
| RNF141 | 0.71 | 0.30 | -0.41 | - | 0.30 | 0.76 | 0.46 | Y |
| CCT4 | 0.69 | 0.44 | -0.25 | - | 0.62 | 1.08 | 0.46 | Y |
| SSBP4 | 0.32 | 1.32 | 1.00 | - | -0.30 | 0.15 | 0.46 | Y |
| RRAGC | 0.73 | 0.22 | -0.51 | - | 0.60 | 1.06 | 0.46 | Y |
| CREG | -0.26 | -0.55 | -0.28 | - | -0.54 | -0.08 | 0.46 | Y |
| SIRT2 | 0.89 | 0.77 | -0.13 | - | -0.32 | 0.14 | 0.46 | Y |
| GTF2IRD2 | -0.47 | -0.39 | 0.07 | - | -0.26 | 0.19 | 0.46 | Y |
| ULK3 | 1.42 | 0.97 | -0.45 | - | -0.53 | -0.08 | 0.45 | Y |
| GNG12 | -0.88 | -0.39 | 0.49 | - | -0.33 | 0.12 | 0.45 | Y |
| HMG20B | 0.20 | -0.21 | -0.41 | - | -0.49 | -0.05 | 0.45 | Y |
| SSPN | 0.88 | 0.10 | -0.77 | - | -0.19 | 0.26 | 0.44 | Y |
| RPP40 | -0.46 | -0.88 | -0.42 | - | 0.41 | 0.86 | 0.44 | Y |
| LSM1 | -0.54 | -0.29 | 0.25 | - | -0.87 | -0.42 | 0.44 | Y |
