# Supplementary material for: Profiling the Dynamics of a Human Phosphorylome Reveals New Components in HGF/c-Met Signaling
Source: PLoS One. 2013 Sep 2;8(9):e72671. doi: 10.1371/journal.pone.0072671 (PMC3759380; doi:10.1371/journal.pone.0072671)
Supplement: Table S3 — List of 88 protein hits in pathway analysis ( Figs. 4b and c ). The KEGG pathways were listed here only if their enrichment scores were larger than 3 and p-values based on hypergeometric model were less than 0.05 after false discovery rate (FDR) multiple-test correction. (DOC) [file pone.0072671.s006.doc]

**Supplemental Table 3 | List of 88 protein hits in pathway analysis (Figs. 4b and c).** The KEGG pathways were listed here only if their enrichment scores were larger than 3 and p-values based on hypergeometric model were less than 0.05 after false discovery rate (FDR) multiple-test correction.

| **KEGG Pathway** | **Enrichment score** | **P-value** | **Protein hits (in Fig. 4b) involved in the pathway** | | | | |
| --- | --- | --- | --- | --- | --- | --- | --- |
| Cell cycle | 4.30 | 7.83E-04 | E2F3 | GADD45A | MCM2 | MCM7 | PCNA |
|  |  |  | RBL2 | SMAD4 | YWHAE | YWHAH |  |
| Pathways in cancer | 3.19 | 7.83E-04 | ARAF | BCR | CRK | E2F3 | HIF1A |
|  |  |  | MAX | MITF | NCOA3 | NFKBIA | RAD51 |
|  |  |  | RXRA | SMAD4 |  | | |
| MAPK signaling pathway | 3.19 | 2.08E-03 | CRK | DDIT3 | GADD45A | HSPA1L | MAPKAPK3 |
|  |  |  | MAX | NR4A1 | PRKACB | RPS6KA2 | RPS6KA5 |
| Neurotrophin signaling pathway | 4.01 | 2.57E-03 | CAMK2D | CRK | NFKBIA | PRKCD | RPS6KA5 |
|  |  |  | YWHAE | YWHAH |  | | |
| Chronic myeloid leukemia | 4.53 | 2.57E-03 | ARAF | BCR | CRK | E2F3 | NFKBIA |
|  |  |  | SMAD4 |  | | | |
| DNA replication | 3.58 | 1.16E-02 | MCM2 | MCM7 | PCNA | POLE | PRIM1 |
